# Aglodorols A–J, undescribed terpenoids with multidimensional neuroprotective activities from *Aglaia odorata* Lour.

**DOI:** 10.1007/s13659-025-00563-2

**Published:** 2026-01-09

**Authors:** Meng Ding, Yue-Han Wang, Chen-Hao Liu, Wang-Xiao Tan, Li-Ming Hu, Ke-Wu Zeng, Peng-Fei Tu, Yong Jiang

**Affiliations:** State Key Laboratory of Natural and Biomimetic Drugs, School of Pharmaceutical Sciences, Peking University, Beijing, 100191 PR China

**Keywords:** *Aglaia odorata* Lour., Diterpenoids, Triterpenoids, Anti-neuroinflammation, Anti-ferroptosis, Neuroprotective activity

## Abstract

Three unprecedented triterpenoids (**1**–**2**, **8**), seven novel diterpenoids (**13**–**16**, **19**–**21**), and 12 known compounds (**3**–**7**, **9**–**12**, **17**–**18**, **22**) were isolated from the tender branches and leaves of *Aglaia odorata* Lour. Structural elucidation was achieved through integrated spectroscopic analysis, quantum chemical calculations (NMR/ECD), and single-crystal X-ray diffraction. All isolates were evaluated for neuroprotective effects. Compounds **3**, **9**–**11**, **13**, **17**, and **18** showed significant protective effects against oxygen-glucose deprivation/reperfusion (OGD/R)-mediated nerve injury in PC12 cells at 10 µM, while compounds **3** and **19** exhibited potent anti-excitotoxicity activity in the L-glutamate-induced HT22 cells at 20 µM. Strikingly, triterpenoids** 1**,** 2**, and **11** displayed remarkable activity against RSL3-induced PC12 cell death with EC_50_ values ranging from 1.16 to 1.74 μM. Compound **22** exhibited the most significant inhibitory activity among the isolates against nitric oxide (NO) release in lipopolysaccharide (LPS)-activated BV2 cells with an IC_50_ value of 22.41 µM.

**Graphical Abstract:**

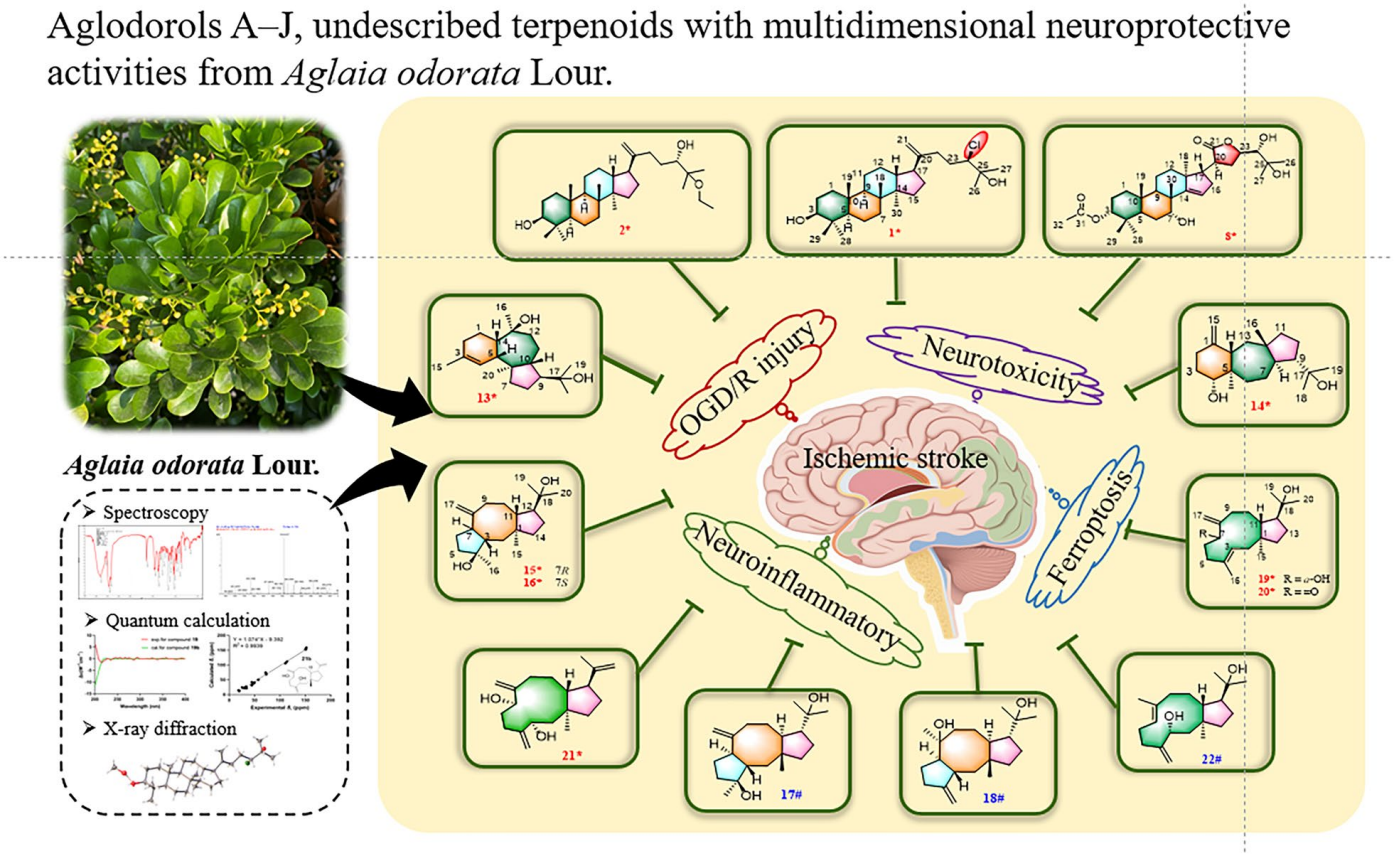

**Supplementary Information:**

The online version contains supplementary material available at 10.1007/s13659-025-00563-2.

## Introduction

The genus *Aglaia*, the largest genus of the family Meliaceae, contains approximately 150 species and primarily distributed across tropical and subtropical Asia [1, 2]. The genus holds significant agricultural and industrial importance, with selected species serving as regionally valuable fruit crops and premium timber sources [2, 3].

*Aglaia odorata* Lour., widely planted as a greening tree in China, has been systematically documented in Chinese medical compendia for its properties in activating blood circulation, resolving stasis, subduing swelling, and relieving pain [4]. Additionally, the flowers of *Aglaia odorata* are brewed into a floral tea, which is traditionally consumed to alleviate alcohol intoxication and cleanse the lungs. Contemporary studies have substantiated its broad bioactivity profile encompassing antiproliferative, antifungal, antiviral, and insecticidal effects [1]. Notably, recent investigations revealed its promising neuroprotective potential. Wang et al. demonstrated the cerebroprotective efficacy of *A. odorata* extract using both an in vivo middle cerebral artery occlusion model and an in vitro oxygen-glucose deprivation/reperfusion (OGD/R) model [4]. Yin et al. further investigated the anti-neuroinflammatory activity of the extract and isolates of *A. odorata* using the lipopolysaccharide (LPS)-induced BV-2 cell model and evaluated the therapeutic effect of rocaglaol on neuroinflammation in mice [5].

Studies have shown that the pathophysiological cascade following ischemic stroke involves intricate interplay between neuronal autotoxicity and peripheral damage mechanisms. This complex process includes excitatory amino acid surges, oxidative stress amplification, dysregulated inflammatory responses, and programmed cell death pathways [6]. To adequately investigate the neuroprotective effects of *A. odorata*, the phytochemical exploration of *A. odorata* was performed to give the isolation and structural elucidation of 22 terpenoids, including 10 previously unreported compounds aglodorols A–J (**1**–**2**, **8**, **13**–**16**, **19**–**21**), and 12 known compounds (**3**–**7**, **9**–**12**, **17**–**18**, **22**). The neuroprotective activities of these isolates were evaluated by a comprehensive multi-model screening system including OGD/R model, the glutamate-induced neurotoxicity model, the LPS-induced neuroinflammation model, and the ferroptosis model.

## Results and discussion

### Structural elucidation of compounds

**Fig. 1:**
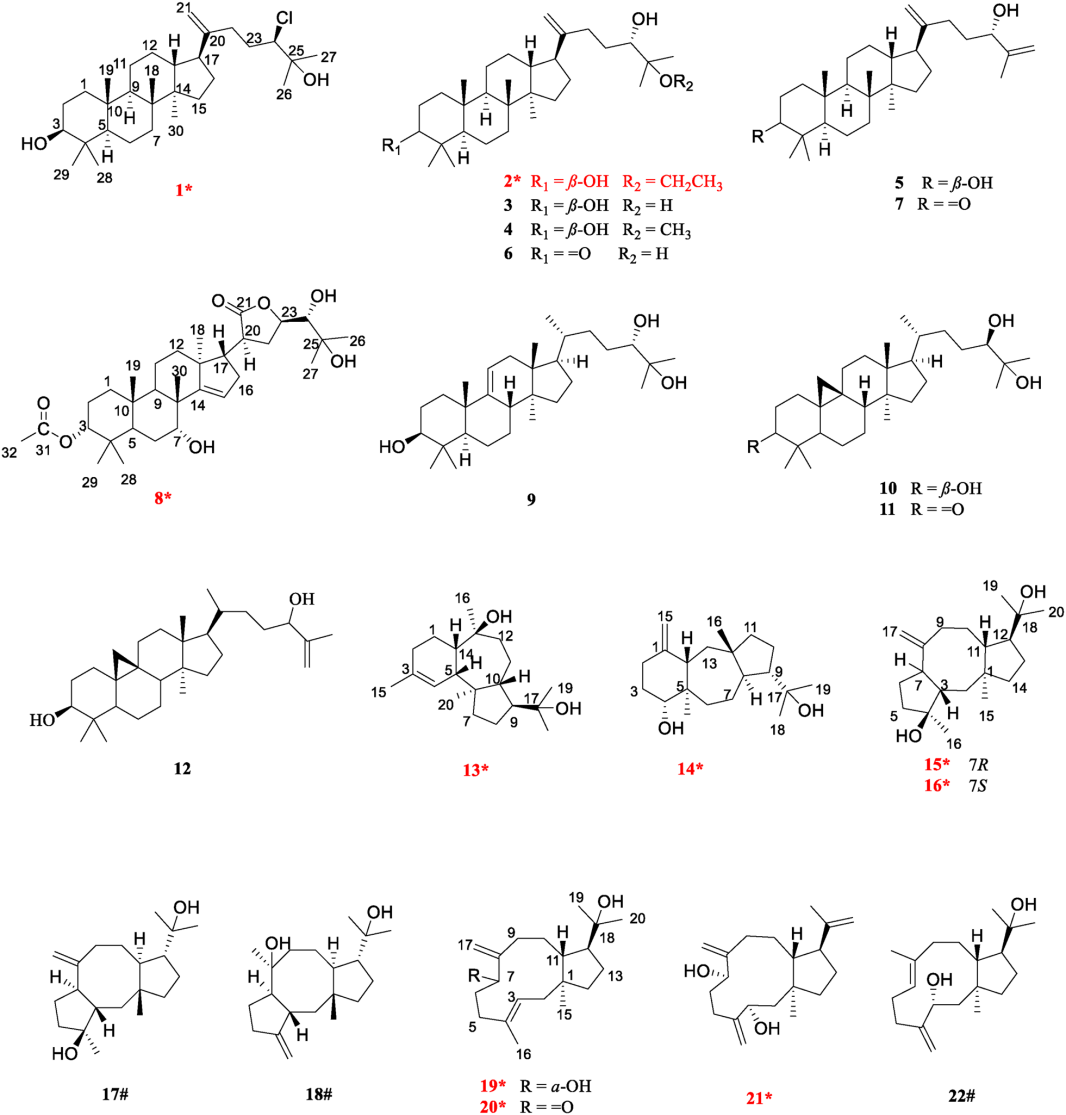
Structures of compounds **1**–**22** (new compounds were marked with *, and known compounds featuring first-established stereochemical configurations with #)

A total of 22 secondary metabolites were isolated, including 10 new compounds (**1**–**2**, **8**, **13**–**16**, and **19**–**21**) along with 12 known compounds (**3**–**7**, **9**–**12**, **17**–**18**, and **22**) (Fig. 1). Notably, compound **1** is a rare chlorine-containing dammarane-type triterpene, and compound **8** is the first reported apotirucallane-type triterpenoid containing a five-membered lactone ring in the side chain. In addition, dolastane-type diterpenes (**13**–**14**) and fusicoccane-type diterpenes (**15**–**18**), believed to be found only in sea creatures [7], have now been reported in higher plants for the first time. The remaining 12 known compounds were identified as 3,24,25‐trihydroxy‐dammar‐20‐ene (**3**) [8], 25-methoxy-5*α*-dammar-20-en-3*β*,24-diol (**4**) [9], dammara-20,25-dien-3*β*,24*α*-diol (**5**) [10], 24,25-dihydroxy-dammar-20-en-3-one (**6**) [8], 24-hydroxydammara-20,25-dien-3-one (**7**) [9], (24*S*)-5*α*-lanost-9(11)-ene-3*β*,24,25-triol (**9**) [11], (24*R*)-cycloartane-3*β*,24,25-triol (**10**) [12], (24*R*)-9,19-cyclolanost-3-one-24,25-diol (**11**) [13], 3*β*-hydroxy-25-methylenecycloartan-24-ol (**12**) [14], barbifusicoccin A (**17**) [15], barbifusicoccin B (**18**) [15], and (1*R*, 3*S*, 7*E*, 11*S*, 12*R*)-dolabella-4(16),7-dien-3,18-diol (**22**) [16] by comparison of spectroscopic data with literature. In addition, the absolute configurations of the known compounds **17**, **18**, and **22** were determined through ECD calculations.

Aglodorol A (**1**) was obtained as colorless massive crystals, mp 167–168 °C, whose molecular formula was deduced to be with a chlorine atom as C_30_H_51_O_2_Cl, from the relative abundance ratio of 3:1 for [M + HCOO]^−^ ion at *m*/*z* 523.3557 (calcd for C_31_H_52_O_4_Cl, 523.3560) and *m*/*z* 525.3554 [M + HCOO + 2]^−^ in the HRESIMS spectrum. The IR spectrum shows stretching bands of hydroxy group (3320 cm^− 1^), chlorine substitution (806 cm^− 1^), and double bond (1643 cm^− 1^). The UV spectrum exhibits maximum absorption bands at 203 nm, suggesting the presence of double bond.

**Fig. 2:**
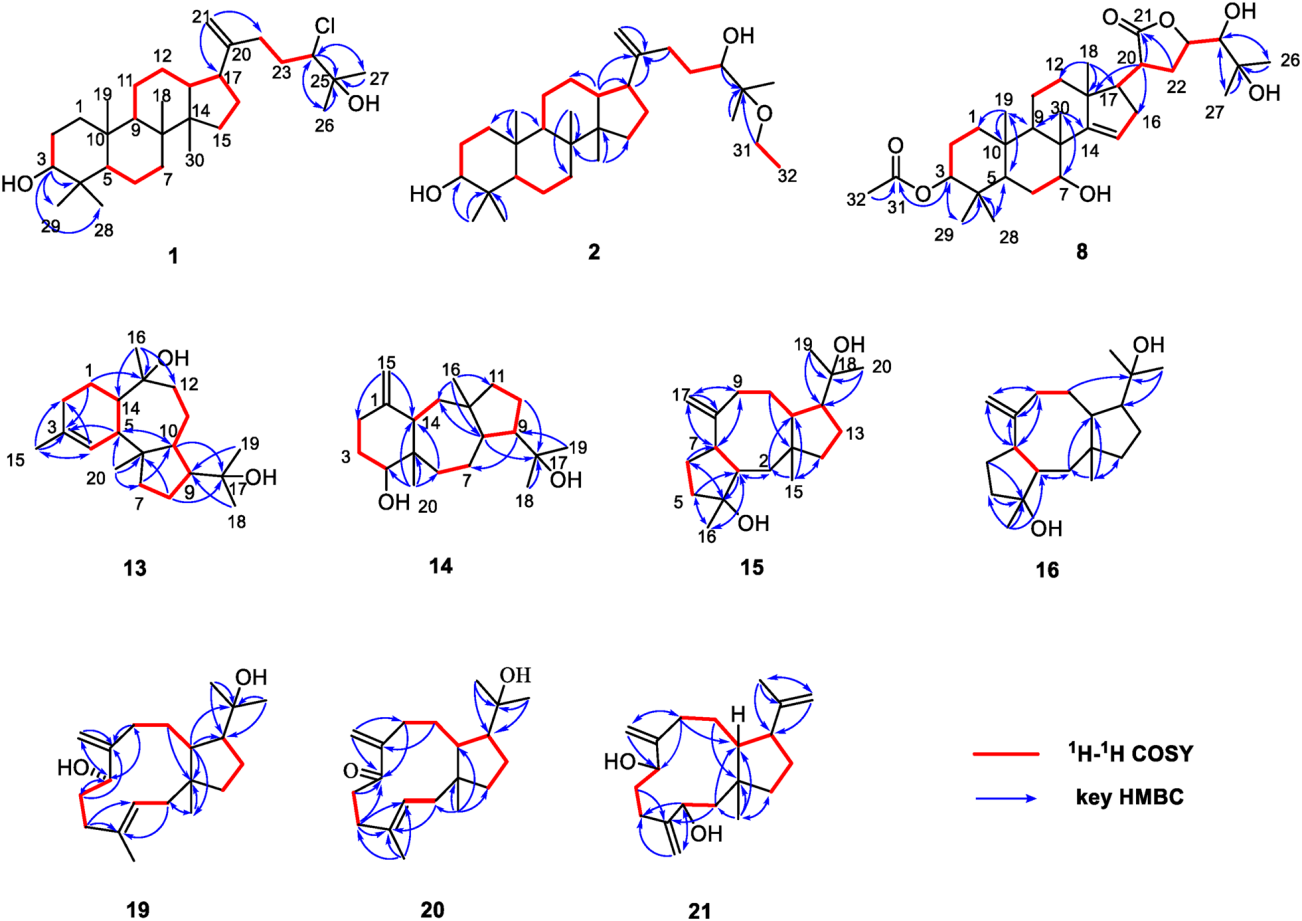
^1^H^1^H COSY and key HMBC correlations of compounds **1**–**2**, **8**, **13**–**16**, and **19**–**21**

The ^1^H NMR data present seven methyl singlet [*δ*_H_ 1.31 (H_3_-26), 1.29 (H_3_-27), 0.98 (H_3_-18), 0.97 (H_3_-28), 0.87 (H_3_-30), 0.84 (H_3_-19), and 0.77 (H_3_-29)] and two terminal olefinic methylene [*δ*_H_ 4.78 and 4.70 (s, H_2_-21)] protons (Table 1). The analysis of ^13^C NMR and HSQC spectra of compound **1** reveals 30 carbon signals, including seven methyl, 11 methylene, six methine, and six quaternary carbons, which are very similar to those of the reported compound **3** [8]. Given the molecular formula of **1**, it was deduced that one of the three hydroxy groups in **3** is substituted by a chlorine atom. Detailed comparison of the NMR data shows that the most significant difference in chemical shifts between **1** (*δ*_C_ 74.0) and **3** (*δ*_C_ 78.3) is at C-24, suggesting that the chlorine atom may be substituted at C-24. The ^1^H–^1^H COSY correlations (H-22a/H-23a and H-23b/H-24) and the HMBC correlations (from H-24 to C-22/C-23/C-25/C-26/C-27, from H_3_-26 to C-24/C-25/C-27, and from H_3_-27 to C-24/C-25/C-26) determine the structure of the side chains (Fig. 2). Therefore, the planar structure of **1** was established. Considering the relative scarcity of naturally occurring chlorinated phytochemicals, the possibility of **1** being an artifact was rigorously investigated. A freshly prepared methanol extract of the title plant was immediately analyzed using UPLC/Qtrap-MS/MS in multiple reaction monitoring (MRM) mode. The metabolite **1** was unambiguously detected in this initial extract (Fig. S11), which effectively rules out its formation during long-term storage or the subsequent isolation procedures. Thus, we propose **1** as an enzyme-catalyzed halogenated product, with the responsible halogenase originating either from the plant’s own biosynthetic machinery [17] or from endophytic bacteria associated with the plant host [18].

**Table 1:** ^1^H (500 MHz) and ^13^C (125 MHz) NMR data of compounds **1**, **2**, and **8** (*δ* in ppm, *J* in Hz) in Chloroform-*d*_1_

No.	1		2		8	
	*δ*_H_ (*J* in Hz)	*δ*_C_, type	*δ*_H_ (*J* in Hz)	*δ*_C_, type	*δ*_H_ (*J* in Hz)	*δ*_C_, type
1*α*	0.95, m	39.2, CH_2_	0.97, m	39.1, CH_2_	1.36, m	33.3, CH_2_
1*β*	1.71, m		1.71, m			
2*α*	1.59, m	27.6, CH_2_	1.57, m	27.4, CH_2_	1.59, m	22.87, CH_2_
2*β*	1.62, m		1.62, m		1.88, m	
3	3.20, dd (11.4, 4.8)	79.1, CH	3.20, dd (11.5, 4.9)	79.0, CH	4.65, m	78.2, CH
4		39.1, C		39.0, C		36.2, C
5	0.73, d (11.6)	56.0, CH	0.73, dd (11.9, 2.2)	55.8, CH	1.95, dd (11.4, 4.2)	42.1, CH
6*α*	1.54, m	18.4, CH_2_	1.53, m	18.3, CH_2_	1.73, m	23.7, CH_2_
6*β*	1.42, m		1.44, m			
7*α*	1.57, m	35.5, CH_2_	1.56, m	35.4, CH_2_	3.92, t (2.9)	72.4, CH
7*β*	1.23, m		1.28, m			
8		40.6, C		40.4, C		44.6, C
9	1.28, m	51.1, CH	1.30, m	50.9, CH	2.00, dd (11.4, 8.2)	41.7, CH
10		37.3, C		37.2, C		37.7, C
11*α*	1.51, m	21.5, CH_2_	1.51, m	21.3, CH_2_	1.71, m	16.3, CH_2_
11*β*			1.19, m		1.50, m	
12*α*	1.05, m	25.2, CH_2_	1.05, m	25.0, CH_2_	2.12, m	32.2, CH_2_
12*β*	1.55, m		1.56, m			
13	1.69, m	45.0, CH	1.67, m	45.5, CH		47.0 C
14		49.6, C		49.4, C		161.6, C
15*α*	1.11, m	31.4, CH_2_	1.10, m	31.4, CH_2_	5.49, d (2.9)	119.4, CH
15*β*	1.61, m		1.60, m			
16*α*	1.93, m	29.0, CH_2_	1.90, m	29.0, CH_2_	2.30, m	33.2, CH_2_
16*β*	1.39, m		1.44, m			
17	2.16, m	47.4, CH	2.20, m	48.2, CH	2.29, m	54.1, CH
18	0.98, s	15.8, CH_3_	0.97, s	15.6, CH_3_	1.06, s	20.3, CH_3_
19	0.84, s	16.4, CH_3_	0.84, s	16.2, CH_3_	0.90, s	15.3, CH_3_
20		151.4, C		153.0, C	2.76, m	39.7, CH
21a	4.78, s	108.2, CH_2_	4.71, s	107.5, CH_2_		177.9, C = O
21b	4.70, s		4.76, s			
22a	2.08, m	31.8, CH_2_	2.00, m	31.2, CH_2_	2.24, m	29.8, CH_2_
22b	2.33, m		2.30, m		2.41, q (11.8)	
23a	1.72, m	31.7, CH_2_	1.48, m	29.8, CH_2_	4.62, m	77.6, CH_2_
23b	2.01, m		1.52, m			
24	3.86, d (11.3)	74.0, CH	3.46, m	76.4, CH	3.29, d (1.3)	76.1, CH
25		72.9, C		77.3, C		72.7, C
26	1.31, s	26.7, CH_3_	1.11, s	19.4, CH_3_	1.34, s	26.8, CH_3_
27	1.29, s	25.2, CH_3_	1.14, s	21.5, CH_3_	1.29, s	26.7, CH_3_
28	0.97, s	28.2, CH_3_	0.98, s	28.0, CH_3_	0.85, s	27.7, CH_3_
29	0.77, s	15.5, CH_3_	0.77, s	15.4, CH_3_	0.89, s	21.9, CH_3_
30	0.87, s	16.1, CH_3_	0.87, s	15.9, CH_3_	1.07, s	28.2, CH_3_
31			3.43, m	56.4, CH_2_		171.2, C
32			1.16, t (7.1)	16.1, CH_3_	2.07, s	21.7, CH_3_

**Fig. 3:**
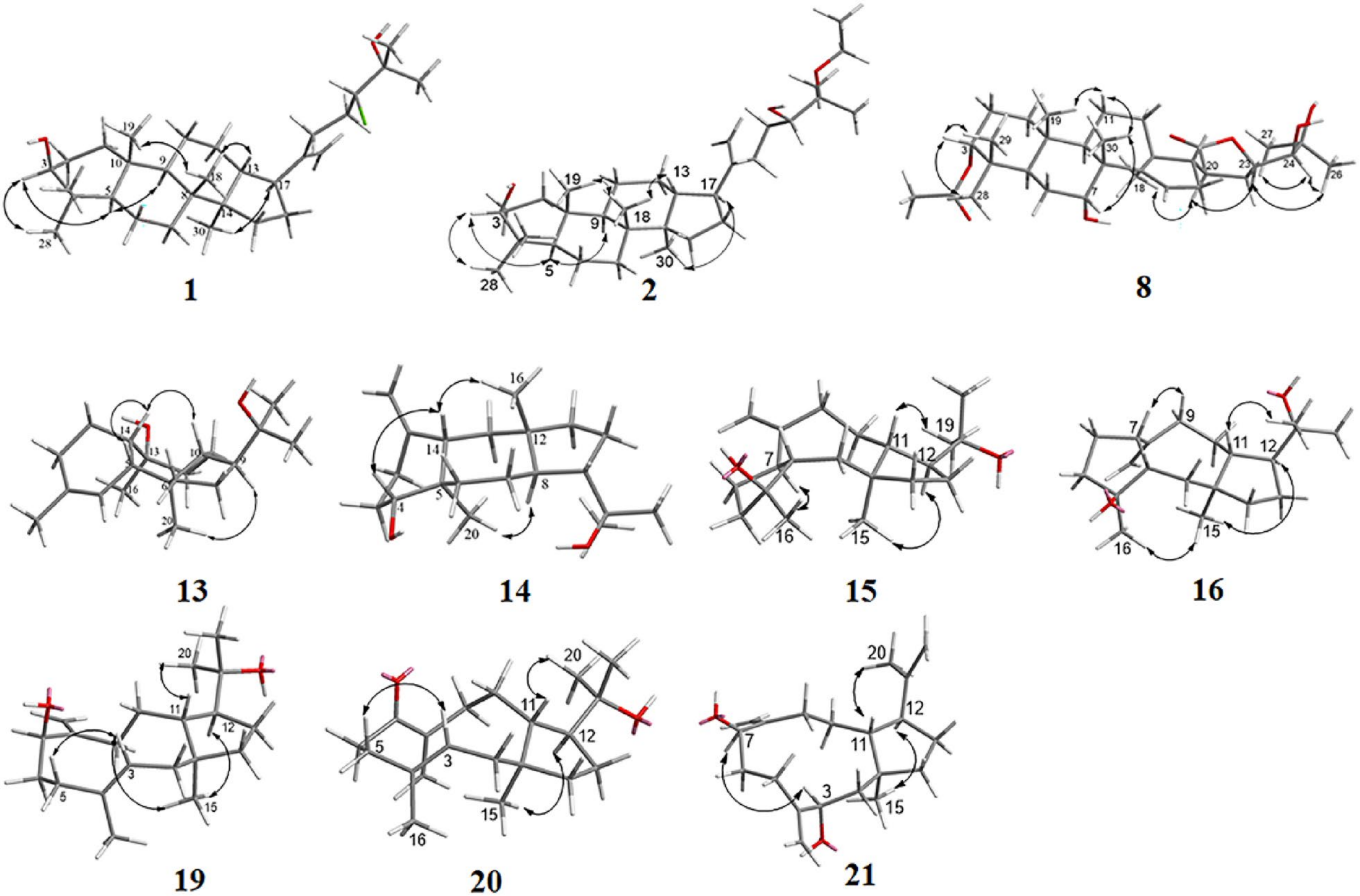
Key NOESY correlations of compounds **1**–**2**, **8**, **13**–**16**, and **19**–**21**

**Fig. 4:**
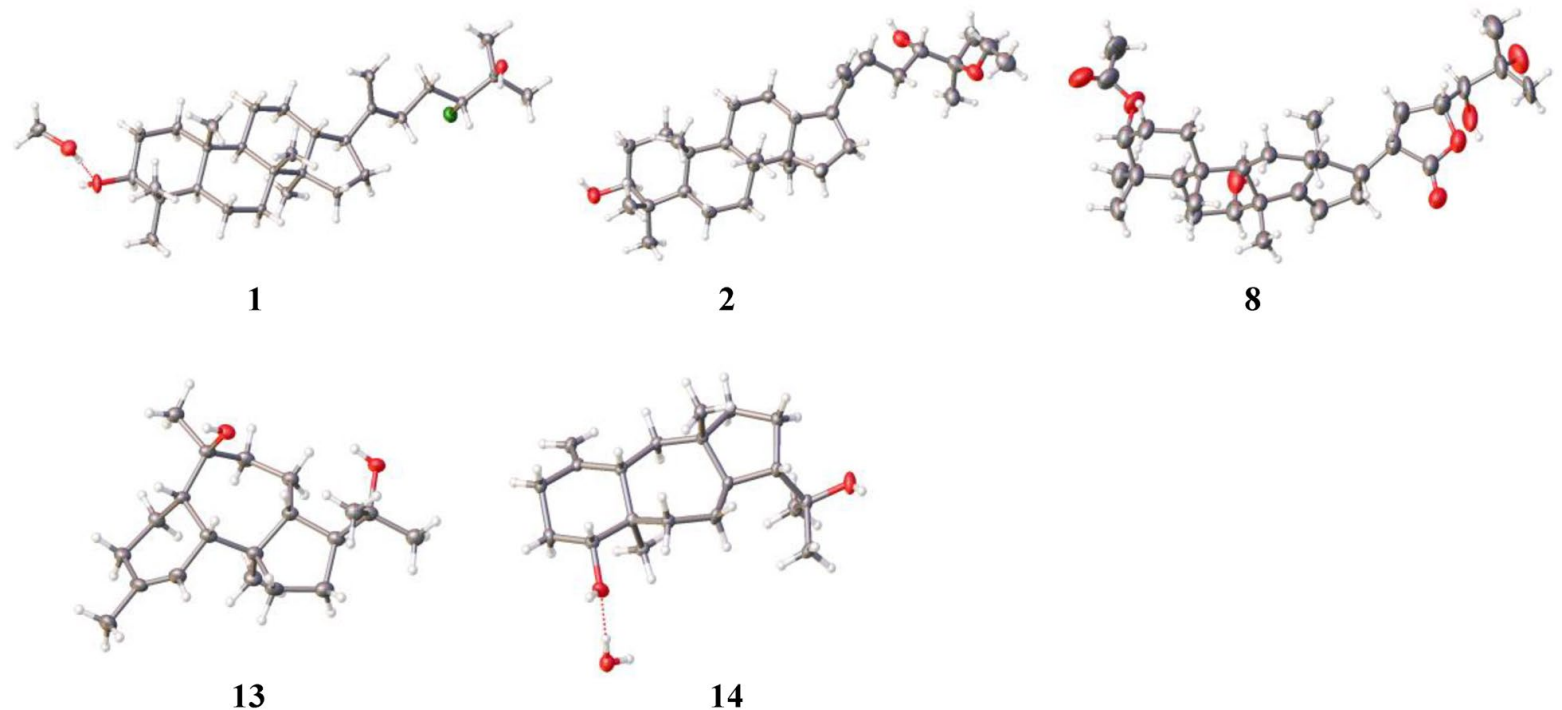
ORTEP drawings of compounds **1**, **2**, **8**, **13**, and **14**

The NOESY correlations of H-3 with H-5, H-5 with H-9, H-17 with H_3_-30, and H_3_-18 with H_3_-19/H-13 suggest that** 1** is with a common dammarane-type triterpene skeleton. These correlations also support a *β*-orientation of the hydroxy group at C-3 (Fig. 3). Subsequently, the absolute configuration of **1** was determined by X-ray crystal diffraction as 3*S*, 5*R*, 8*R*, 9*R*, 10*R*, 13*R*, 14*R*, 17*S*, 24*R* (Fig. 4). Thus, the structure of compound **1** was assigned as shown in Fig. 1.

Aglodorol B (**2**) was isolated as colorless needle crystals, mp 165–167 °C, with a molecular formula of C_32_H_56_O_3_ based on HRESIMS data (*m*/*z* 489.4301 [M + H]^+^, calcd for C_32_H_57_O_3_, 489.4308). The IR spectrum indicates the presence of hydroxy group (3413 cm^− 1^) and double bond (1638 cm^− 1^).

The ^1^H NMR data present seven methyl singlet [*δ*_H_ 1.14 (H_3_-27), 1.11 (H_3_-26), 0.98 (H_3_-28), 0.97 (H_3_-18), 0.87 (H_3_-30), 0.84 (H_3_-19), and 0.77 (H_3_-29)], a group of ethyloxyl [*δ*_H_ 1.16 (3H, t, *J* = 7.1 Hz, H_3_-32), 3.34 (2H, m, H_2_-31)], and two terminal olefinic methylene [*δ*_H_ 4.76 and 4.71 (s, H_2_-21)] protons (Table 1). The analysis of ^13^C NMR and HSQC spectra of compound **2** shows 32 carbon signals, including eight methyl, 12 methylene, six methine, and six quaternary carbons. The ^1^H and ^13^C NMR data of **2** are comparable to those of the reported compound **4** [9], except that the OCH_3_ at C-25 is replaced by the OCH_2_CH_3_ in **2**, which is confirmed by the ^1^H–^1^H COSY correlations of H_2_-31/H_3_-32 and the HMBC correlations from H_2_-31 to C-25 (Fig. 2). Thus, the planar structure of **2** was established.

Similarly, the NOESY correlations of H-3 with H-5, H-5 with H-9, H-17 with H_3_-30, H_3_-18 with H_3_-19, and H-13 with H_3_-18, which are consistent with those of the dammarane-type triterpene skeleton, supported the *β*-orientation of the hydroxy group at C-3 (Fig. 3). Subsequently, the absolute structure of **2** was determined as 3*S*, 5*R*, 8*R*, 9*R*, 10*R*, 13*R*, 14*R*, 17*S*, 24*S* by a single crystal X-ray diffraction experiment (Fig. 4). Considering the use of ethanol in the extraction procedure and the possibility of transetherification of **4** to **2** during ethanol extraction, we performed a rapid extraction of plant material using methanol and analyzed it by UPLC-Qtrap-MS/MS (Fig. S22). However, there is no detectable signal for compound **2** in the extract. This result provides evidence that compound **2** may be naturally present in the plant at a level below the detectable level. However, this result could not exclude the possibility that compound** 2** may be formed from the methoxy-substituted analog of compound **4** via an acid-catalyzed transetherification during ethanol extraction.

Aglodorol C (**8**) was obtained as colorless needle crystals, mp 241–242 °C. Its molecular formula is C_32_H_50_O_7_ based on HRESIMS data (*m*/*z* 591.3533 [M + HCOO]^−^, calcd for C_33_H_51_O_9_, 591.3528), requiring eight degrees of unsaturation. The IR spectrum shows signals of hydroxy group (3456 cm^− 1^) and ester carbonyl group (1724, 1767 cm^− 1^).

The ^1^H NMR spectrum shows eight methyl singlets [*δ*_H_ 2.07 (H_3_-32), 1.34 (H_3_-26), 1.29 (H_3_-27), 1.07 (H_3_-30), 1.06 (H_3_-18), 0.90 (H_3_-19), 0.89 (H_3_-29), and 0.85 (H_3_-28)], one olefinic proton [*δ*_H_ 5.49 (1H, d, *J* = 2.9 Hz, H-15)], and four oxygenated methine protons [*δ*_H_ 4.65 (1H, m, H-3), 4.62 (1H, m, H-23), 3.92 (1H, t, *J* = 2.9 Hz, H-7), and 3.29 (1H, d, *J* = 1.3 Hz, H-24)] (Table 1). The ^13^C NMR spectrum of compound **8** shows 32 carbon signals, which are further classified by HSQC experiments as eight methyl, seven methylene, nine methine, and eight quaternary carbons. According to the chemical shifts, two olefinic carbons [*δ*_C_ 161.6 (C-14), 119.4 (C-15)], five oxygenated carbons [*δ*_C_ 78.2 (C-3), 77.6 (C-23), 76.1 (C-24), 72.7 (C-25), 72.4 (C-7)], and two ester carbonyl carbons [*δ*_C_ 171.2 (C-31), 177.9 (C-21)] could be determined. The NMR data of **8** are similar to those of agladoral A, an apotirucallane-type triterpene isolated from this genus, except that one group of the acetoxyl protons in agladoral A has disappeared and a carbonyl carbon (*δ*_C_ 177.9) in **8** substitutes a hydroxyl carbon (*δ*_C_ 97.1) in agladoral A [19]. The HMBC correlations from H-3 to C-31 and from H_3_-32 to C-31 indicate that the acetoxy group is located at C-3 (Fig. 2). The ^1^H–^1^H COSY correlations of H-17/H-20/H_2_-22/H-23/H-24, as well as the HMBC correlations from H-20 and H_2_-22 to C-21, from H_2_-22 to C-20/C-23, and from H-20 to C-17/C-13/C-16 confirm the presence of a five-membered lactone ring linked at C-17 (Fig. 2).

Similar to agladoral A, the NOESY correlations of H-3 with H_3_-28/H_3_-29, H-7 with H_3_-30, and H_3_-30/H_3_-19 with H-11*β* support the *β*-orientation of H-3 and H-7 (Fig. 3). Moreover, the NOESY correlations of H-17 with H-12a, H_3_-18 with H-20, and H-20 with H-23 illustrate the *α*-orientation of H-20 and H-23 in **8** (Fig. 3). The small coupling constant (*J*_23, 24_ = 1.27 Hz) indicates that they are in a *gauche* relationship [20], indicating that H-24 is *β*-oriented. Due to a suboptimal Flack parameter of 0.13(15) obtained from the single crystal X-ray diffraction experiment, only the relative configuration of compound **8** could be determined (Fig. 4 and Table S1). Consequently, the absolute configuration was subsequently determined as 3*S*, 5*R*, 7*R*, 8*R*, 9*R*, 10*S*, 13*S*, 17*S*, 20*S*, 23*R*, 24*S* by the ECD calculation (Fig. 8).

Aglodorol D (**13**), obtained as colorless massive crystals, mp 152–153 °C, has a molecular formula of C_20_H_33_O_2_ with four indices of hydrogen deficiency based on the HRESIMS (*m*/*z* 305.2480 [M − H]^−^, calcd for 305.2481) and supported by ^13^C NMR data. The IR spectrum shows the absorptions at 3326 cm^− 1^ for hydroxy group and 1705 cm^− 1^ for double bond.

The ^1^H NMR data present five methyl singlets [*δ*_H_ 1.68 (H_3_-15), 1.27 (H_3_-16), 1.19 (H_3_-18), 1.17 (H_3_-19), 0.83 (H_3_-20)] (Table 2). The analysis of ^13^C NMR and HSQC spectra of compound **13** reveals 20 carbon signals, including five methyl, six methylene, five methine, and four quaternary carbons. Among them, two olefinic (*δ*_C_ 134.2, 124.7) and two oxygenated (*δ*_C_ 76.8, 74.2) carbons are easily recognized. The alkene bond accounts for one of the four degrees of unsaturation, and the left three ones indicate that **13** contains a tricyclic system. The ^1^H–^1^H COSY correlations of H_2_-2/H_2_-1/H-14/H-5/H-4, and the HMBC correlations from H-4 to C-2/C-5/C-14/C-15, from H_2_-1 to C-3/C-5/C-14, from H-5 to C-3, and from H_3_-15 to C-2/C-3/C-4 suggest the presence of a six membered ring A and a methyl is linked to C-3 (Fig. 2). Another spin system, H_2_-7/H_2_-8/H-9/H-10, is elucidated by analysis of the ^1^H–^1^H COSY spectrum, and the HMBC correlations from H-10 to C-6/C-9, from H-7a to C-9/C-10, and from H_2_-8 to C-6/C-10 construct a five membered ring B (Fig. 2). Besides, the ^1^H–^1^H COSY correlations of H-10/H-11a/H-12a and the HMBC correlations from H-12b and H-14 to C-13 define the seven-membered ring C (Fig. 2). The HMBC correlations of H_2_-1 with C-13, H-14 with C-1/C-13, and H-5 with C-3/C-4/C-6/C-10 indicate that C-5 and C-14 are shared by the six- and seven-membered rings. While, the HMBC correlations of H-9 with C-11, H-10 with C-5, H-7 with C-5/C-6, and H_3_-20 with C-5/C-6/C-7 indicate that C-6 and C-10 are shared by five- and seven-membered rings (Fig. 2). Hereby, **13** was deduced to have a 6/7/5 membered ring system. The presence of the 2-hydroxypropan-2-yl is indicated by the HMBC correlations of H_3_-18 and H_3_-19 with C-9/C-17 (*δ*_C_ 74.2), and its position is fixed on C-9 of the five-membered ring. The HMBC correlations of H_3_-16 [*δ*_H_ 1.27 (3H, s)] with C-12, C-13, and C-14, and of H_3_-20 with C-5, C-6, C-7, and C-10 deduce the positions of two aliphatic methyl groups at C-13 and C-6, respectively. At the same time, in combination of the chemical shift of C-13 (*δ*_C_ 76.8) and the formula weight, a hydroxy is determined to be located at C-13. The planar structure of **13** was thus established.

**Table 2:** ^1^H (500 MHz) and ^13^C (125 MHz) NMR data of compounds **13**, **14**, **15**, **16**, **19**, **20**, and **21** (*δ* in ppm, *J* in Hz) in Chloroform-*d*_1_

No.	13		14		15		16		19		20		21	
	*δ*_H_ (*J* in Hz)	*δ*_C_, type	*δ*_H_ (*J* in Hz)	*δ*_C_, type	*δ*_H_ (*J* in Hz)	*δ*_C_, type	*δ*_H_ (*J* in Hz)	*δ*_C_, type	*δ*_H_ (*J* in Hz)	*δ*_C_, type	*δ*_H_ (*J* in Hz)	*δ*_C_, type	*δ*_H_ (*J* in Hz)	*δ*_C_, type
1	1.53, m	22.4, CH_2_		150.3, C		43.6, C		44.0, C		46.8, C		45.9, C		44.4, C
2*α*	1.98, m	31.4, CH_2_	2.08, m	35.8, CH_2_	1.39, m	39.4, CH_2_	1.39, m	35.7, CH_2_	2.09, m	42.5, CH_2_	1.98, dd (13.6, 11.9)	42.4, CH_2_	1.72, m	49.5, CH_2_
2*β*			2.29, m		1.79, m				1.75, dd (14.5, 2.9)		1.68, dd (13.6, 3.9)			
3*α*		134.2, C	1.84, m	32.3, CH_2_	1.47, m	51.7, CH	1.76, m	49.5, CH	5.31, dd (11.5, 2.9)	123.9, CH	5.07, dd (11.9, 3.9)	124.7, CH	4.37, t (6.0)	72.8, CH
3*β*			1.47, m											
4	5.39, d (5.3)	124.7, CH	3.32, dd (11.8, 4.4)	77.9, CH		80.7, C		82.9, C		135.0, C		132.6, C		151.9, C
5	2.23, m	47.4, CH		44.7, C	1.80, m	38.9, CH_2_	1.65–1.67, m	38.5, CH_2_	2.15, m	37.4, CH_2_	2.23–2.25 m	39.7, CH_2_	2.25, m/2.06, m	25.2, CH_2_
6*α*		50.9, C	1.57, m	35.3, CH_2_	1.86, m	24.7, CH_2_	1.68, m	28.8, CH_2_	2.21, m	34.1, CH_2_	3.40, m	34.0, CH_2_	2.04, m	32.8, CH_2_
6*β*			2.12, m		1.64, m		1.74, m		2.11, m		1.96, m		1.93, m	
7*α*	1.56, m	42.3, CH_2_	2.02, m	29.0, CH_2_	2.04, m	51.7, CH	3.32, q (8.9)	49.3, CH	3.87, dd (8.2, 2.5)	75.2, CH		206.3, C	4.24, dd (8.3, 3.4)	71.8, CH
7*β*	1.35, m													
8	1.37, m	25.5, CH_2_	1.65, m	47.9, CH		152.5, C		152.8, C		153.4, C		150.1, C		150.9, C
9*α*	1.63, m	56.8, CH	1.78, m	57.9, CH	1.96, m	32.8, CH_2_	2.26, m	31.6, CH_2_	2.01, m	34.7, CH_2_	2.49 (m),	28.6, CH_2_	2.26, m	34.0, CH_2_
9*β*					2.34, m		2.02, m		1.90, m		2.35 (m)		2.09, m	
10a	2.24, m	47.5, CH	1.86, m	26.0, CH_2_	1.53, m	32.1, CH_2_	1.48, m	32.4, CH_2_	1.50, m	29.2, CH_2_	1.21 (m)	31.3, CH_2_	1.29, m	30.2, CH_2_
10b			1.44, m		1.97, m		1.89, m		1.84, m		1.39 (m)		1.71, m	
11*α*	1.19, m	28.2, CH_2_	1.34, m	44.2, CH_2_	1.64, m	41.5, CH	1.64, m	42.0, CH	1.42, m	43.8, CH	1.28 (m)	42.2, CH	1.59, m	47.9, CH
11*β*	2.11, m													
12*α*	1.82, m	31.7, CH_2_		45.7, C	1.75, m	53.4, CH	1.74, m	53.4, CH	1.67, m	59.4, CH	1.52 (m)	59.5, CH	2.45, m	57.2, CH
12*β*	1.59, m													
13a		76.8, C	1.74, m	43.6, CH_2_	1.74, m	25.1, CH_2_	1.75, m	25.2, CH_2_	1.28, m	27.1, CH_2_	1.26 (m)	27.4, CH_2_	1.38, m	28.8, CH_2_
13b			1.49, m		1.34, m		1.38, m		1.69, m		1.62 (m)		1.86, m	
14*α*	1.66, m	51.8, CH	1.93, m	46.9, CH	1.16, m	40.0, CH_2_	1.10, m	38.5, CH_2_	1.42, m	44.2, CH_2_	1.29 (m)	43.7, CH_2_	1.41, m	44.3, CH_2_
14*β*					1.42, m		1.51, m						1.49, m	
15a	1.68, s	23.9, CH_3_	4.79, s	107.9, CH_2_	0.93, s	23.2, CH_3_	0.87, s	23.2, CH_3_	1.02, s	21.8, CH_3_	0.83 (s)	21.9, CH_3_	0.93, s	18.4, CH_3_
15b			4.61, s											
16	1.27, s	34.2, CH_3_	1.00, s	18.6, CH_3_	1.13, s	24.4, CH_3_	1.26, s	24.9, CH_3_	1.59, s	16.4, CH_3_	1.60(s)	15.9, CH_3_	5.14, s/4.94, s	110.7, CH_2_
17a		74.2, C		73.9, C	4.95, br. s	109.1, CH_2_	4.94, s	113.4, CH_2_	5.18, s	108.3, CH_2_	6.01 (s),	126.0, CH_2_	5.22, s	113.6, CH_2_
17b					4.84, br. s		4.82, s		5.13, s		5.88 (s)		4.96, s	
18	1.19, s	29.3, CH_3_	1.18, s	29.1, CH_3_		74.6, C		74.4, C		74.0, C		71.6, C		150.2, C
19a	1.17, s	26.8, CH_3_	1.18, s	27.2, CH_3_	1.14, s	27.2, CH_3_	1.16, s	27.7, CH_3_	1.19, s	25.2, CH_3_	1.00 (s)	25.7, CH_3_	4.78, s	110.6, CH_2_
19b													4.66, s	
20	0.83, s	16.9, CH_3_	0.75, s	9.5, CH_3_	1.15, s	29.1, CH_3_	1.17, s	28.9, CH_3_	1.25, s	31.6, CH_3_	1.12 (s)	31.8, CH_3_	1.74, s	19.3, CH_3_
18–OH											4.15 (s)			

The relative configuration of **13** was determined by the NOESY spectrum (Fig. 3). The NOESY correlations of H-14 with H-5/H-10 suggest that these protons are on the same side of the molecule, while the correlations of H_3_-20 with H-9/H_3_-16 suggest that they are on the other side of the molecule. Therefore, a *cis*-fusion between the six- and seven-membered rings and a *trans*-fusion between the seven- and five-membered rings were deduced. Subsequent single crystal X-ray diffraction analysis assigned the absolute configuration of **13** as 5*R*, 6*R*, 9*S*, 10*R*, 13*S*, 14*S* (Fig. 4).

Aglodorol E (**14**) was obtained as colorless needle crystals, mp 145–146 °C. Its molecular formula was deduced as C_20_H_34_O_2_ based on the HRESIMS (*m*/*z* 305.2480, [M − H]^−^, calcd for 305.2481) and ^13^C NMR data with the presence of four hydrogen-deficiency indices. The IR absorption bands at 3385 cm^− 1^ and 1641 cm^− 1^ reveal the presence of hydroxy group and double bond.

The ^1^H NMR data include four methyl singlets [*δ*_H_ 1.18 (H_3_-18 and H_3_-19), 1.00 (H_3_-16), 0.75 (H_3_-20)], and a pair of terminal olefinic methylene signals [*δ*_H_ 4.79 (1H, s), 4.61 (1H, s)] (Table 2). The analysis of ^13^C NMR data and HSQC spectrum of compound **14** reveals 20 carbon signals, including four methyl, eight methylene, four methine, and four quaternary carbons. Among them, two olefinic carbons (*δ*_C_ 150.3, 107.9) and two oxygenated carbons (*δ*_C_ 77.9, 73.9) are particularly obvious. Similar to **13**, compound **14** should also contain a tricyclic system, since the alkene bond accounts for only one of the four degrees of unsaturation. The ^1^H–^1^H COSY correlations of H_2_-2/H_2_-3/H-4 and the HMBC correlations from H_2_-15 to C-14 and C-2, from H-4 to C-3 and C-5, and from H-14 to C-5 correspond to a six membered ring and an exomethylene group is defined at C-1 and C-15 (Fig. 2). The COSY cross peaks of H-8/H-9//H_2_-10/H_2_-11, and the HMBC correlations from H_2_-11 to C-9/C-10/C-12, from H-8 to C-11/C-12, and from H_3_-16 to C-8/C-11/C-12 define the presence of a five membered ring. In addition, the COSY correlations of H-13a/H-14 and H-6a/H_2_-7/H-8, along with the HMBC correlations of H-8 and H-14 with C-13, and H-6 with C-5/C-14 confirm the existence of a seven membered ring (Fig. 2). The connections of the six membered ring with the seven membered ring, and the seven membered ring with the five membered ring are established by the HMBC correlations of H_3_-20 with C-4/C-5/C-6/C-14, H_3_-16 with C-8/C-11/C-12/C-13, H-4 with C-6, H-9 with C-7, and H-11 with C-13. This led to the determination of the planar structure of **14**.

The relative configuration of **14** was established through NOESY analysis: key correlations of H-14 with H-4/H_3_-16 confirmed that they are on the same side of the fused ring system, tentatively on the *β*-orientation, whereas the observed NOESY interaction of H-8 with H_3_-20 positions them on the opposing *α*-orientation (Fig. 3). The absolute configuration of **14** was further determined by X-ray crystal diffraction to be 4*R*, 5*R*, 8*R*, 9*S*, 12*S*, 14*S* (Fig. 4).

Aglodorol F (**15**) was obtained as colorless solid. Its molecular formula was determined to be C_20_H_34_O_2_ by HRESIMS (*m*/*z* 351.2539 [M + HCOO]^−^, calcd. for C_21_H_35_O_4_, 351.2530). The IR absorption bands at 3405 cm^− 1^ and 1635 cm^− 1^ indicate the presence of hydroxy group and double bond.

The ^1^H NMR data of **15** reveal two distinct terminal olefinic protons [*δ*_H_ 4.95 (1H, br. s), 4.84 (1H, br. s), H-17] and four methyl groups [*δ*_H_ 1.15 (H_3_-20), 1.14 (H_3_-19), 1.13 (H_3_-16), 0.93 (H_3_-15)] (Table 2). The ^13^C NMR and HSQC spectra resolve 20 carbon resonances attributable to two olefinic [*δ*_C_ 152.5 (C-8), 109.1 (C-17)], four methyls [*δ*_C_ 29.1 (C-20), 27.2 (C-19), 24.4 (C-16), 23.2 (C-15)], seven methylene [*δ*_C_ 40.0 (C-14), 39.4 (C-2), 38.9 (C-5), 32.8 (C-9), 32.1 (C-10), 25.1 (C-13), 24.7 (C-6)], four common methine [*δ*_C_ 53.4 (C-12), 51.7 (C-3 and C-7), 41.5 (C-11)], two oxygenated quaternary [*δ*_C_ 80.7 (C-4), 74.6 (C-18)], and one quaternary [*δ*_C_ 43.6 (C-1)] carbons. The olefinic moiety accounts for one of four degrees of unsaturation, so there may be a tricyclic skeleton in the structure of **15**, which is further confirmed by the HMBC and ^1^H–^1^H COSY correlations (Fig. 2). The ^1^H–^1^H COSY correlations of H_2_-5/H_2_-6, H-3/H-7 and the HMBC correlations from H-3 to C-4/C-16, from H_3_-16 to C-3/C-5, from H-5 to C-3/C-4/C-7/C-16, from H-6 to C-4/C-5/C-7, and from H-7 to C-3 correspond to a five membered ring with a methyl group at C-4. The ^1^H–^1^H COSY correlations of H_2_-9/H_2_-10/H-11 and the HMBC correlations from H_2_-2 to C-1/C-3/C-11, from H-7 to C-3/C-8/C-9, from H_2_-17 to C-7/C-8/C-9, from H_2_-9 to C-7/C-8/C-10/C-11/C-17, from H_2_-10 to C-1/C-9/C-11, and from H-11 to C-10 define the presence of an eight membered ring and confirm that the terminal double bond is at the C-8 and C-17. In addition, the ^1^H–^1^H COSY correlations (H-11/H-12/H_2_-13/H_2_-14) and the HMBC correlations (from H-12 to C-1/C-11/C-14, from H_2_-13 to C-1/C-11/C-14, and from H_2_-14 to C-11) confirm the existence of another five membered ring. The 2-hydroxypropan-2-yl is at C-12 as evidenced by the HMBC correlations from H_3_-20 and H_3_-19 to C-12/C-18. The above tricyclic skeleton is subsequently established through the following key HMBC correlations: H-3 to C-2/C-4/C-5, H_3_-15 to C-1/C-2/C-11/C-14, and H_2_-2 to C-14. Thus, the planar structure of compound **15** was determined. It is interesting to note that **15** has the same planar structure as compound **17** [21], but their NMR spectra exhibited significant discrepancies. Notable chemical shift variations (Δ*δ* = 2–6 ppm) for the eight membered ring carbons and C-12/15 may originate from alterations in the chiral configurations at specific positions (C-1, C-3, C-7, C-11, and C-12).

**Fig. 5:**
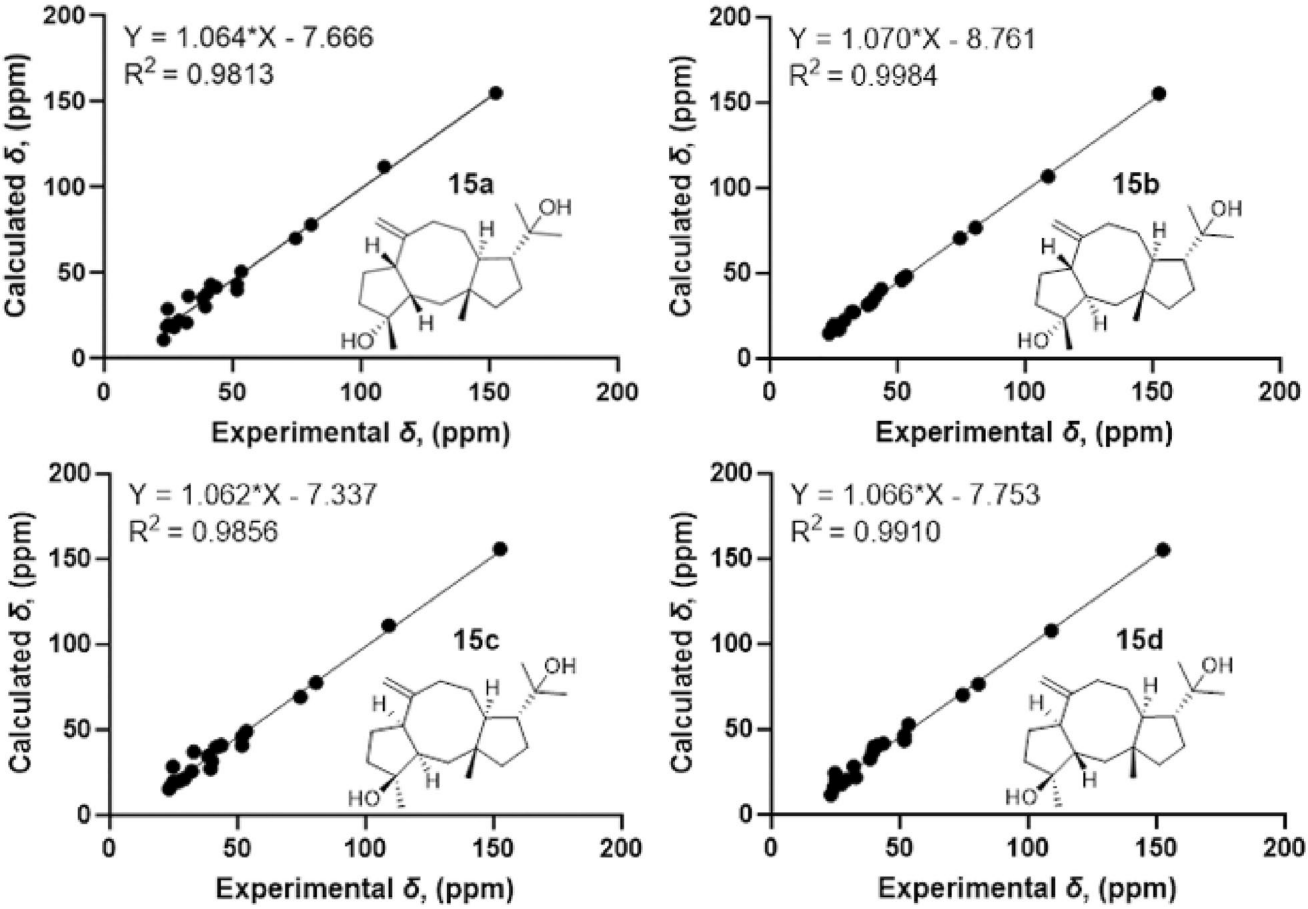
^13^C-NMR calculations of compound **15**

Consistent with compound **17**, the NOESY correlations (Fig. 3) of H-12/H_3_-15 and H-11/H_3_-19 identified the 1R*, 11S*, 12R*-configurations [21]. The NOESY correlation between H_3_-16 and H-7 indicates their cofacial orientation, and on the basis of above, their relative configurations may be as 4R*, 7S* or 4S*, 7R*. However, the configuration at C-3 could not be determined due to the absence of valuable NOESY correlations observed. To establish the relative configuration of compound **15**, the quantum chemical calculation of NMR parameters of four epimers **15a** (1R*, 3S*, 4R*, 7S*, 11S*, 12R*), **15b** (1R*, 3R*, 4R*, 7S*, 11S*, 12R*), **15c** (1R*, 3R*, 4S*, 7R*, 11S*, 12R*), and **15d** (1R*, 3S*, 4S*, 7R*, 11S*, 12R*) were performed, and the result showed that the configuration of **15b** is more consistent with the experimental value of **15** (Fig. 5). Furthermore, the absolute configuration of 1*S*, 3*S*, 4*S*, 7*R*, 11*R*, 12*S* was suggested by the ECD calculation (Fig. 8).

Aglodorol G (**16**) was obtained as colorless solid. Its molecular formula was determined as C_20_H_34_O_2_ based on HRESIMS data (*m*/*z* 351.2536 [M + HCOO]^−^, calcd. for C_21_H_35_O_4_, 351.2541), which is the same as that of compound **15**. The IR absorption band at 3389 cm^− 1^ indicates the presence of hydroxy group.

The ^1^H NMR data of **16** reveal two distinct terminal olefinic protons [*δ*_H_ 4.94 (1H, br. s), 4.82 (1H, br. s)] and four methyl groups [*δ*_H_ 1.26 (H_3_-16), 1.17 (H_3_-20), 1.16 (H_3_-19), 0.87 (H_3_-15)] (Table 2). The ^13^C NMR and HSQC spectra resolve 20 carbon resonances attributable to two olefinic [*δ*_C_ 152.8 (C-8), 113.4 (C-17)], four methyl [*δ*_C_ 28.9 (C-20), 27.7 (C-19), 24.9 (C-16), 23.2 (C-15)], seven methylene [*δ*_C_ 38.5 (C-14 and C-5), 35.7 (C-2), 32.4 (C-10), 31.6 (C-9), 28.8 (C-6), 25.2 (C-13)], four methine [*δ*_C_ 53.4 (C-12), 49.5 (C-3), 49.3 (C-7), 42.0 (C-11)], two oxygenated quaternary [*δ*_C_ 82.9 (C-4), 74.4 (C-18)], and one quaternary [*δ*_C_ 44.0 (C-1)] carbons. Compound **16** was deduced to be an isomer of compound **15** because of their very similar 1D NMR, HMBC and COSY data (Table 2 and Fig. 2).

**Fig. 6:**
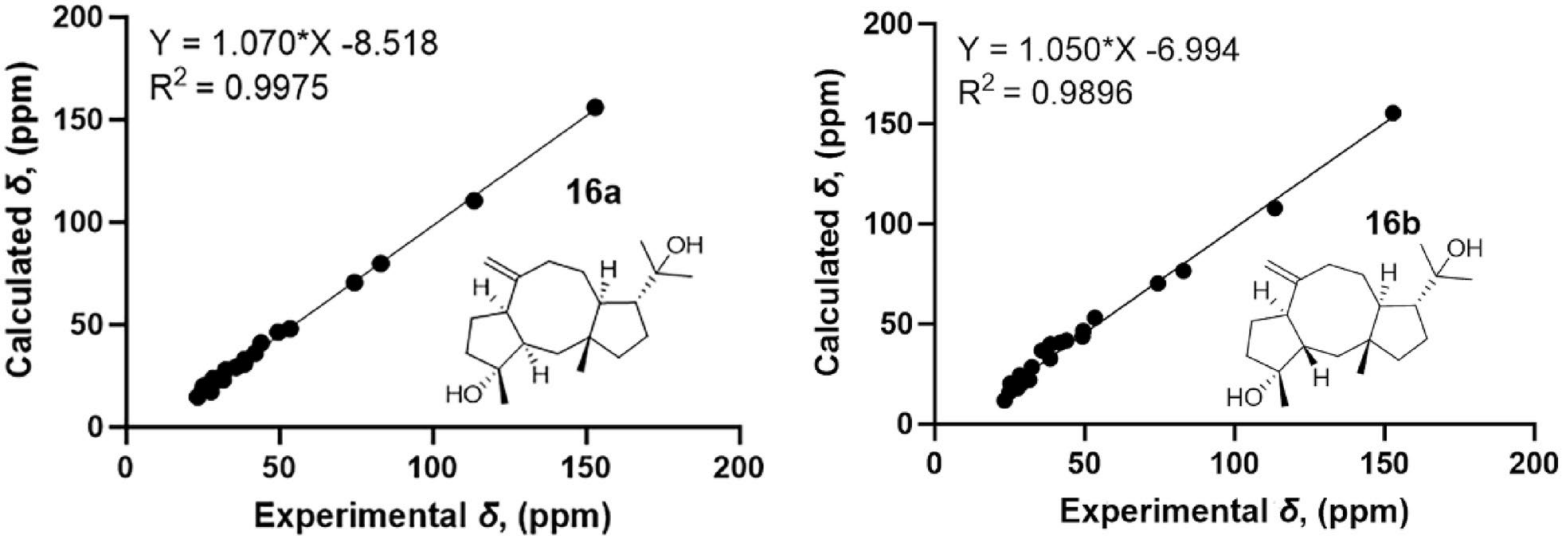
^13^C-NMR calculations of compound **16**

The NOESY correlations of H_3_-15 with H-12/H_3_-16/H-9a indicate that they are in the same direction in space, tentatively assigned as the *β*-orientation, while the NOESY correlations of H-11 with H_3_-19 and H-7 with H-9b suggest that they are in the other direction of the ring system (Fig. 3). Since the configuration at C-3 could not be deduced from the NOESY correlations, the quantum chemical calculations of two epimers **16a** (1R*, 3R*, 4R*, 7R*, 11S*, 12R*) and **16b** (1R*, 3S*, 4R*, 7R*, 11S*, 12R*) were performed. The results showed that the configuration of **16a** is more consistent with the experimental data (Fig. 6). Furthermore, the absolute configuration of 1*S*, 3*S*, 4*S*, 7*S*, 11*R,* 12*S* was suggested by the ECD calculation (Fig. 8).

Aglodorol H (**19**) was obtained as colorless oil with a molecular formula of C_20_H_34_O_2_ as evidenced by HRESIMS (*m*/*z* 305.2487 [M − H]^−^, calcd. for C_20_H_33_O_2_, 305.2486). The IR absorption bands at 3425 cm^− 1^ and 1663 cm^− 1^ reveal the presence of hydroxy group and double bond.

The ^1^H NMR data of **19** reveal three olefinic protons [*δ*_H_ 5.18 (1H, br.s), 5.13 (1H, br. s), 5.31 (1H, dd, *J* = 11.5, 2.9 Hz)], four methyl groups [*δ*_H_ 1.59 (H_3_-16), 1.25 (H_3_-20), 1.19 (H_3_-19), 1.02 (H_3_-15)], and one oxygenated methine proton [*δ*_H_ 3.87 (1H, dd, *J* = 8.2, 2.5 Hz, H-7)] (Table 2). The ^13^C NMR and HSQC spectra resolve 20 carbon resonances attributable to four olefinic [*δ*_C_ 153.4 (C-8), 135.0 (C-4), 123.9 (C-3), 108.3 (C-17)], four methyl [*δ*_C_ 31.6 (C-20), 25.2 (C-19), 21.8 (C-15), 16.4 (C-16)], seven common methylene [*δ*_C_ 44.2 (C-14), 42.5 (C-2), 37.4 (C-5), 34.7 (C-9), 34.1 (C-6), 29.2 (C-10), 27.1 (C-13)], one oxygenated methine [*δ*_C_ 75.2 (C-7)], two methine [*δ*_C_ 59.4 (C-12), 43.8 (C-11)], one oxygenated quaternary [*δ*_C_ 74.0 (C-18)], and one quaternary [*δ*_C_ 46.8 (C-1)] carbons. The two olefinic bonds account for two of the four degrees of unsaturation, so there may be a bicyclic backbone in the structure of **19**. The ^1^H–^1^H COSY correlations (H_2_-2/H-3, H_2_-5/H_2_-6/H-7 and H_2_-9/H_2_-10/H-11) and the HMBC correlations (from H_2_-2 to C-1/C-3/C-4, from H-3 to C-2, from H_2_-5 to C-3/C-4/C-16, from H_2_-6 to C-7/C-8/C-16/C-17, from H-7 to C-6/C-9/C-17, from H_2_-9 to C-8, and from H_2_-10 to C-1) correspond to an eleven membered ring with a methyl group at C-4 and a terminal double bond at C-8 (Fig. 2). In addition, the ^1^H–^1^H COSY correlations (H-12/H_2_-13/H_2_-14) and the HMBC correlations (from H-11 to C-1/C-12/C-13) confirm the existence of a five membered ring. The 2-hydroxypropan-2-yl is at C-12 as evidenced by the HMBC correlations from H_3_-20 and H_3_-19 to C-12/C-18 and from H-11 to C-18. The above bicyclic skeleton is deduced to be connected via C-1 and C-11 as evidenced by the key HMBC correlations of H_2_-2 to C-14 and H_3_-15 to C-1/C-2 (Fig. 2).

**Fig. 7:**
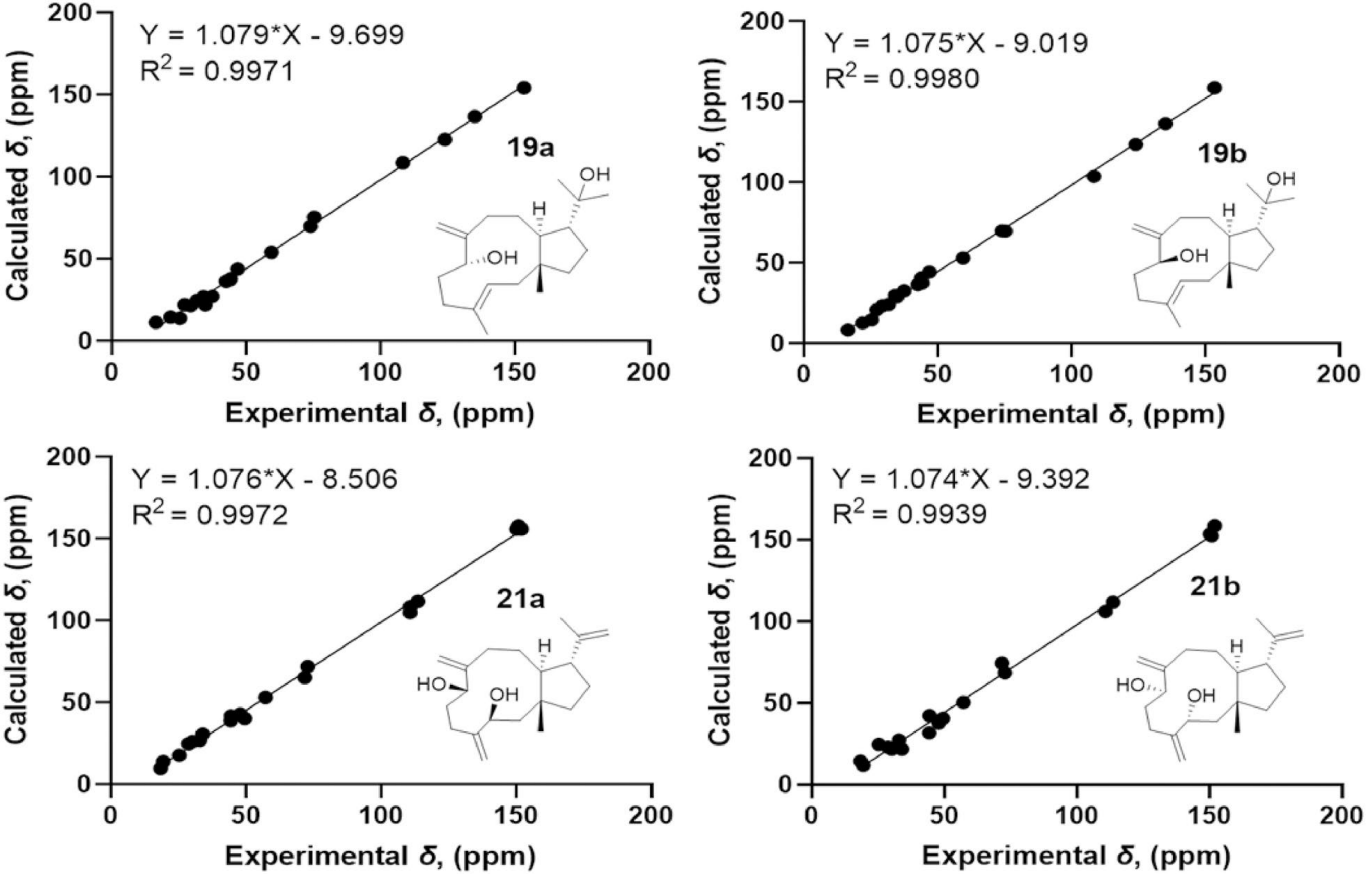
^13^C-NMR calculations of compounds **19** and **21**

**Fig. 8:**
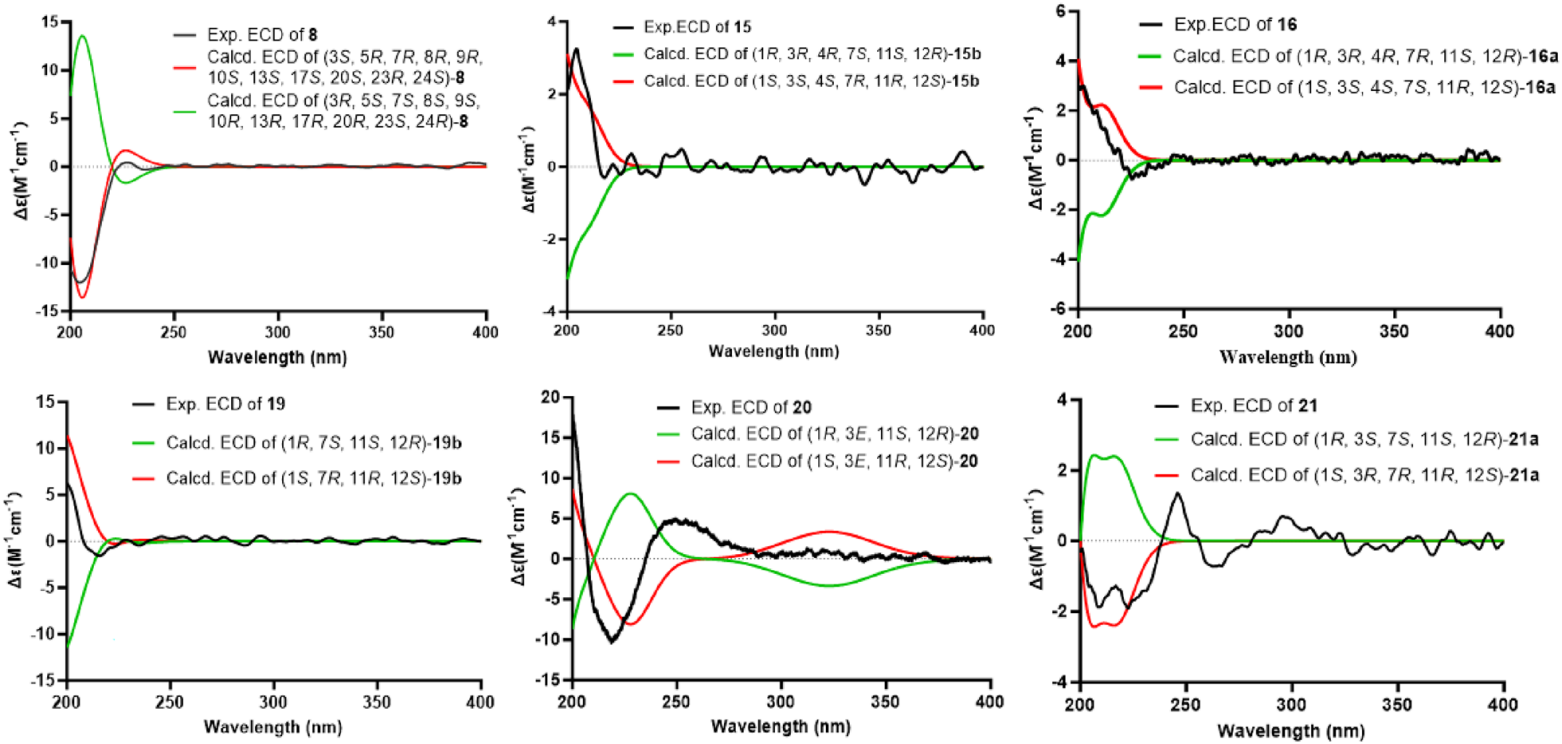
The experimental and calculated ECD spectra of compounds **8**, **15**–**16**, and **19**–**21**

The NOESY correlations of H_3_-15 with H-12 suggest that they are on the same side of the molecule, while the correlations of H-11 with H_3_-18 suggest that they are on the other side of the molecule (Fig. 3). The *E*-configuration of the Δ^3^ double bond is deduced by the NOESY correlations of H-3 with H_2_-5 [16]. In order to establish the relative configuration of 7-OH, the quantum chemical calculations were carried out on **19** (Fig. 7), and the (1R*, 3*E*, 7S*, 11S*, 12R*)-configuration (**19b**) was determined. Furthermore, the ECD calculation suggests the absolute configuration of **19** as 1*S*, 3*E*, 7*R*, 11*R*, 12*S* (Fig. 8).

Aglodorol I (**20**) was obtained as light-yellow oil with a molecular formula of C_20_H_32_O_2_ and five degrees of unsaturation based on HRESIMS (*m*/*z* 303.2327 [M − H]^−^, calcd. for C_20_H_31_O_2_, 303.2324). The 1D and 2D NMR spectra suggest that the structure of **20** is very similar to that of compound **19**. The only difference is that the hydroxy group at C-7 (*δ*_C_ 75.2) in **19** is oxidized to a carbonyl group (*δ*_C_ 206.3) in **20** (Table 2). The absolute configuration of **20** was determined as 1*S*, 3*E*, 11*R*, 12*S* by the ECD calculation (Fig. 8).

Aglodorol J (**21**) was obtained as colorless oil with a molecular formula of C_20_H_32_O_2_ and five degrees of unsaturation based on the HRESIMS (*m*/*z* 303.2325 [M − H]^−^, calcd. for C_20_H_31_O_2_, 303.2330). The IR spectrum shows stretching bands of hydroxy group (3396 cm^− 1^) and double bond (1640 cm^− 1^).

The ^1^H NMR data of **21** present three pairs of terminal double bond protons [*δ*_H_ 5.22 (1H, s, H-17), 5.14 (1H, s, H-16), 4.96 (1H, s, H-17), 4.94 (1H, s, H-16), 4.78 (1H, s, H-19), 4.66 (1H, s, H-19)], two methyl singlets [*δ*_H_ 1.74 (H_3_-20), 0.93 (H_3_-15)], and two oxygenated methine protons [*δ*_H_ 4.37 (1H, t, *J* = 6.0 Hz, H-3), 4.24 (1H, dd, *J* = 8.3, 3.4 Hz, H-7)] (Table 2). The ^13^C NMR and HSQC spectra resolve 20 carbon resonances attributable to six olefinic [*δ*_C_ 151.9 (C-4), 150.9 (C-8), 150.2 (C-18), 113.6 (C-17), 110.7 (C-16), 110.6 (C-19)], two methyl [*δ*_C_ 19.3 (C-20), 18.4 (C-15)], seven methylene [*δ*_C_ 49.5 (C-2), 44.3 (C-14), 34.0 (C-9), 32.8 (C-6), 30.2 (C-10), 28.8 (C-13), 25.2 (C-5)], two oxygenated methine [*δ*_C_ 72.8 (C-3), 71.8 (C-7)], two methine [*δ*_C_ 57.2 (C-12), 47.9 (C-11)], and one quaternary [*δ*_C_ 44.4 (C-1)] carbons. The olefinic moieties account for three degrees of unsaturation, so there should be a bicyclic backbone in the structure of **21**. The ^1^H–^1^H COSY correlations (H_2_-2/H-3, H_2_-5/H_2_-6/H-7, H_2_-9/H_2_-10/H-11/H-12/H_2_-13/H_2_-14) and the HMBC correlations of H-3 with C-2/C-4/C-5/C-16, H_2_-16 with C-3/C-5, H_2_-17 with C-7/C-8/C-9, H_2_-9 with C-8/C-7/C-11, H_2_-10 with C-11/C-1, H_3_-15 with C-1/C-2/C-11/C-14 and H_2_-14 with C-11/C-12/C-15 indicate the bicyclic skeleton of **21** as shown in Fig. 2. An isopropenyl group is located at C-12, confirmed by the HMBC correlations of CH_3_-20/H_2_-19 with C-12. The planar structure of **21** is consistent with that of dolabella-4(16), 8(17), 18(19)-triene-3,7-diol [5]. However, the differences of their NMR data indicate the differences in their stereo structures.

The relative configuration of **21** was determined as 1R*, 3S*, 7S*, 11S*, 12R* (**21a**) by the NOESY correlations (H_3_-15 with H-12, H-3 with H-7, and H-11 with H_3_-20) (Fig. 3) and the quantum chemical calculations (Fig. 7). Further, the absolute configuration of 1*S*, 3*R*, 7*R*, 11*R*, 12*S* was determined by comparison of the experimental ECD and calculated ECD curves (Fig. 8).

Compounds **17**, **18**, and **22** have only been reported with relative configurations previously; here, we determined their absolute configurations by comparison of calculated and experimental ECD results (Fig. S98).

### Investigation neuroprotective activity

The crude extract and several secondary metabolites of *A. odorata* have successively been reported to demonstrate neuroprotective properties in models of cerebral ischaemia–reperfusion injury and neuroinflammation [4, 5]. Thus, we evaluated the isolated compounds in four neurodegeneration cell models.

**Fig. 9:**
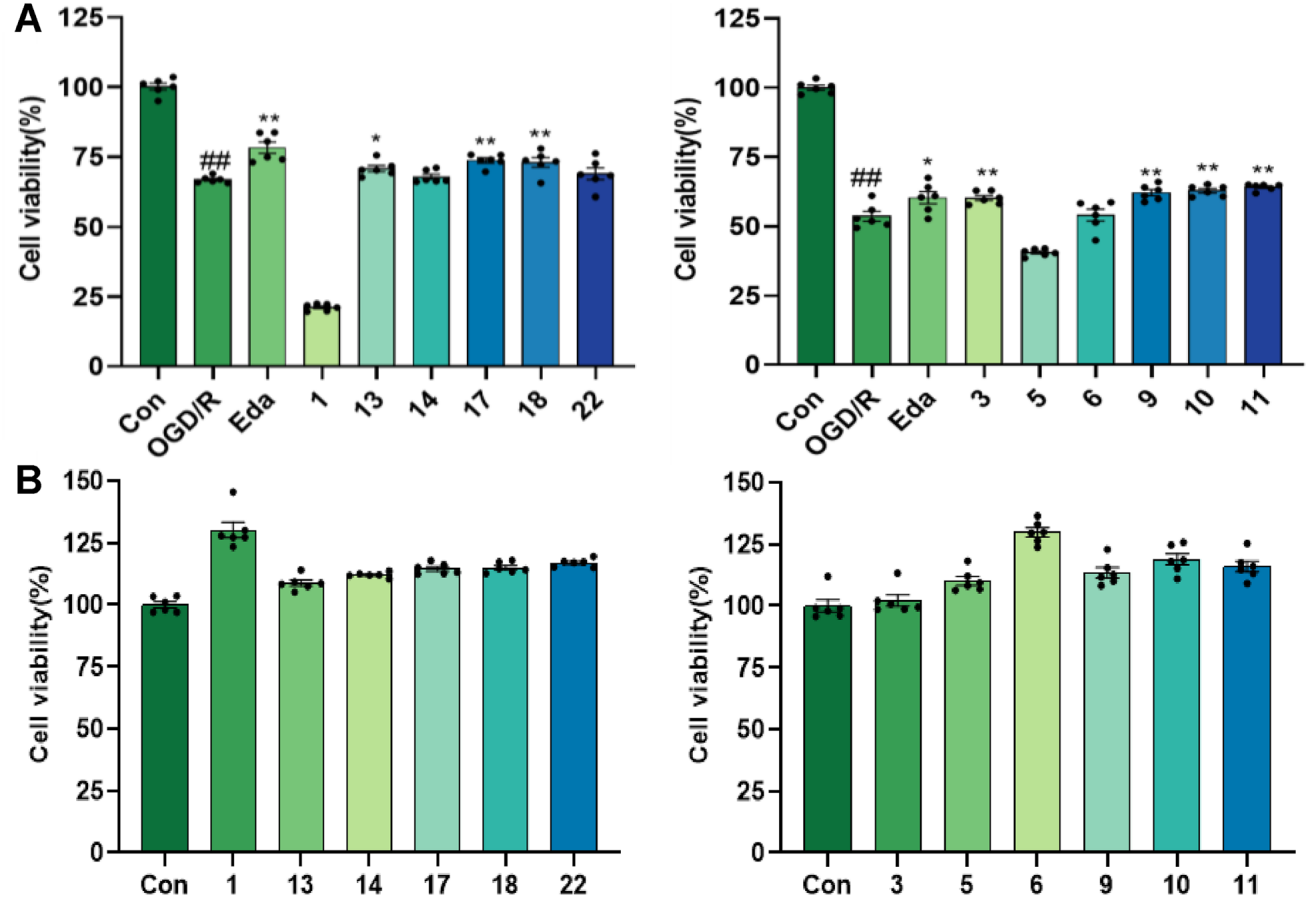
The protective activity of the isolates at 10 µM concentration on OGD/R-mediated nerve injury in PC12 cells. **A** The cell viability of PC12 cells by OGD/R-mediated nerve injury with the treatment of the isolates; **B** Toxicity test of compounds on PC12 cells. Eda (16 μM) was used as positive control; ##*p* < 0.01 compared with control group; *p < 0.05; **p < 0.01 compared with OGD/R group (*n* = 6)

**Fig. 10:**
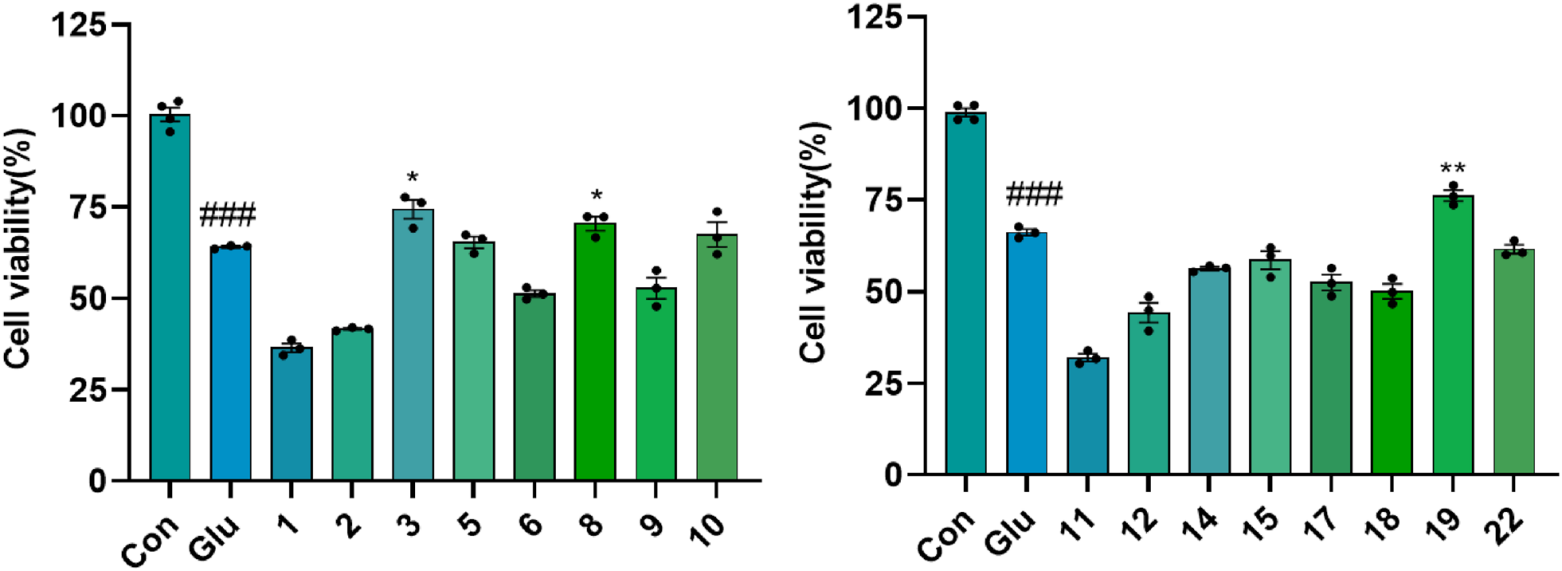
The anti-excitotoxicity activity of the isolates in the L-glutamate-induced HT22 cells at a concentration of 20 µM. ###*p* < 0.001 compared with control group; *p < 0.05, **p < 0.01 compared with Glu group (*n* = 3)

The results showed that compounds **3**,** 9**–**11**, **13**, and **17**–**18** exhibited significant neuroprotective effects against OGD/R-mediated nerve injury in PC12 cells at 10 µM concentration (*P* < 0.05) (Fig. 9). Compounds **3**, **8**, and **19** showed significant protective effects against L-glutamate-induced excitatory damage at a concentration of 20 µM (Fig. 10). In both models, the *ortho*-dihydroxy moiety may serve as a key pharmacophore for triterpenoid-mediated neuroprotection. Notably, at non-cytotoxic concentrations, compound **3** conferred protection against OGD/R injury, whereas structural analogs **1** and **5** exacerbated OGD/R-induced damage in PC12 cells. This observation suggests that *chloro*-substitution modification at C24 or reductive modification at C25 may potentiate damage mechanisms underlying OGD/R.

**Fig. 11:**
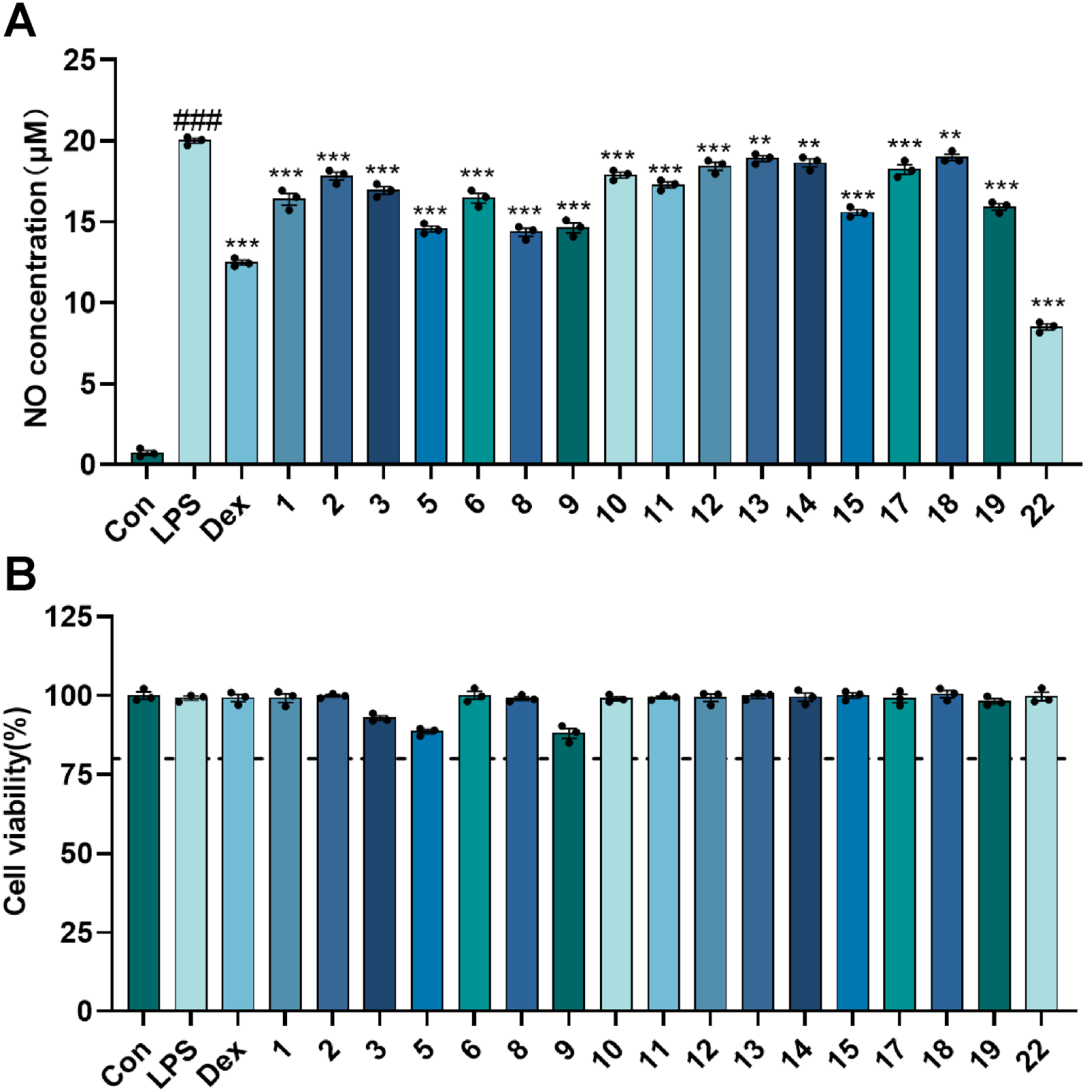
The inhibitory activity of the isolates at a concentration of 20 μM on LPS-induced NO production in BV-2 cells. **A** The concentration of NO produced by LPS-induced BV2 cells with the treatment of the isolates; **B** Cell viability of BV-2 cells with the treatment of the isolates. Dex (10 μM) was used as positive control; ###*p* < 0.001 compared with control group; **p < 0.01, ***p < 0.001 compared with LPS group (*n* = 3)

**Fig. 12:**
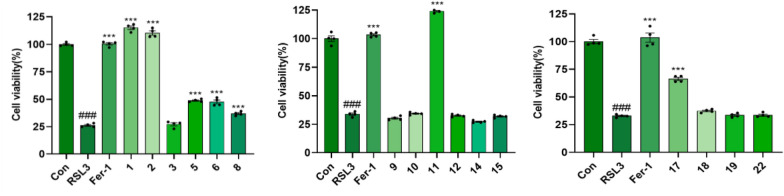
The anti-ferroptosis activity of the isolates at 10 µM concentration in RSL3-induced PC12 cell death. Fer-1 (10 μM) was used as positive control; ###*p* < 0.001 compared with control group; ***p < 0.001 compared with RSL3 group (*n* = 3)

In the LPS-induced neuroinflammation model, all tested compounds significantly suppressed NO production at a concentration of 20 µM (Fig. 11), with compound **22** demonstrating the highest potency (IC_50_ = 22.41 ± 0.32 µM) (Table 3). Compounds **1**–**2**, **5**–**6**, **8**, **11**, and **17** possessed remarkably significant anti-ferroptosis activity at 10 µM concentration (*P* < 0.001, Fig. 12), with the EC_50_ values of compounds **1**, **2**, **11**, and **17** ranging from 1.16 to 9.45 µM (Table 3). In ferroptosis models, the terminal *ortho*-dihydroxy moiety is dispensable for triterpenoid activity. Strikingly, compounds **10** (inactive) and **11** (highly active), differing only at C-3, establish that the oxidation state at C-3 is critical for the biological activity of cycloartane-type triterpenoids.

**Table 3:** Various activity data of active compounds

Com.	IC_50_ (μM) ^*a*^	EC_50_ (µM) ^*a*^
	NO inhibition	Anti-ferroptosis
**1**	NT^*b*^	1.74 ± 0.11
**2**	NT^*b*^	1.36 ± 0.10
**5**	> 50	NT^*b*^
**8**	> 50	NT^*b*^
**9**	> 50	NT^*b*^
**11**	NT^*b*^	1.16 ± 0.02
**17**	NT^*b*^	9.45 ± 0.39
**22**	22.41 ± 0.32	NT^*b*^
**Dex**^***c***^	6.78 ± 0.13	–
**Fer-1**^***d***^	–	0.0059 ± 0.00038

^*a*^ IC_50_ and EC_50_ values are presented as mean ± SD (*n* = 3)

^*b*^ NT, not tested

^*c*^ Dex, Dexamethasone, positive control for LPS-induced NO production in BV2 cells

^*d*^ Fer-1, Ferrostatin-1, positive control for RSL3-induced terroptotic in PC12 cells

## Conclusion

In summary, the phytochemical investigation of *A. odorata* led to the successful isolation and identification of 22 terpenoids, including three new triterpenoids, seven novel diterpenoids, alongside 12 known compounds. Notably, diterpenoids are very rare in the genus *Aglaia*, particularly dolastane- and fusicoccane-type derivatives, which were previously documented predominantly in marine organisms. In four mechanistically distinct models of neuronal cell injury, triterpenoids showed superior neuroprotective activity compared to diterpenoids. This study not only expands the chemical diversity of terpenoids in the genus *Aglaia*, but also provides a mechanistic explanation for the neuroprotective activity of *A. odorata* against ischemic stroke through a multi-model evaluation system. These findings further provide a scientific basis for exploring this plant in anti-ischemic stroke drug discovery.

## Experimental section

### General experimental materials

UV spectra were recorded on a Shimadzu UV-2450 spectrophotometer (Shimadzu Co., Tokyo, Japan). Nicolet NEXUS-470 FTIR spectrometer (MA, USA) was used to record IR spectra. The NMR spectra were performed on an INOVA-500 NMR spectrometer (Varian Co., Palo Alto, CA, USA), using methanol-*d*_4_ and chloroform-*d*_1_ as solvents, and the chemical shifts were referenced to the solvent residual peak. HRESIMS data were performed on a Waters Xevo G2 Q-TOF MS (Waters Co., Milford, MA, UK) or an Orbitrap Exploris 240 (ThermoFisher). Optical rotations were measured using a Rudolph Autopol III automatic polarimeter (Rudolph Research, Fairfield, New jersey, USA). The experimental ECD spectra were obtained on a J-810 spectrophotometer (JASCO, Japan). HPLC was carried out on an Agilent 1260 series instrument with a Thermo ScientificTM AccucoreTM C18 5 μm column (250 × 9.4 mm). Single crystal diffraction data were measured on a XtaLAB Synergy R, HyPix diffractometer (Rigaku, Japan). Silica gel (100–200 mesh and 200–300 mesh, Qingdao Marine Chemical, Co., Ltd., P.R. China) and Sephadex LH-20 (18–110 μm, Pharmacia, Sweden) and ODS (50 μM, YMC, Japan) were used for open column chromatography (CC). TLC analyses were carried out on the precoated silica gel GF 254 plates (Qingdao Marine Chemical Co., Ltd., P.R. China).

Mouse BV-2 microglial cells, PC12 cells, and HT22 cells were sourced from Peking Union Medical College (Beijing, China). BV-2 cells and HT22 cells were maintained in high glucose Dulbecco’s Modified Eagle Medium (Gibico, GrandIsland, USA), while PC12 cells were maintained in RPMI 1640 medium (Gibico, GrandIsland, USA), both supplemented with 10% fetal bovine serum (ABW, Shanghai, China), penicillin and streptomycin (100 U/mL and 100U/mL). All cell lines were kept at 37 °C in a 5% CO_2_ in air humidified environment.

Methylthiazolyldiphenyl-tetrazolium bromide (MTT) was from Solarbio (Beijing, China). LPS was purchased from Sigma (St. Louis, MO, USA). Ferrostatin-1 (Fer-1) was supplied by MedChem Express (Monmouth Junction, NJ, USA). L-glutamate was offered by Shanghai Yuanye Biotechnology Co., Ltd. (Shanghai, China).

### Plant material

The tender branches and leaves of *Aglaia odorata* Lour. were collected in Zhaoqing, Guangdong Province, China, in August 2020. And they were identified by one of the authors, Prof. PengFei Tu. The voucher specimen (No. 20200820) was deposited in Herbarium of the Modern Research Center for Traditional Chinese Medicine, Peking University.

### Extraction and isolation

The air-dried and milled tender branches and leaves of *A. odorata* (6.8 kg) were extracted successively with 95% and 50% ethanol (3 × 60.0 L) under reflux condition. The filtrates were combined and evaporated under reduced pressure to obtain a crude extract (1.5 kg), which was suspended in water, and extracted sequentially with petroleum ether (PE), ethyl acetate (EA), and *n*-butanol. After concentration, the PE extract (260.0 g), EA extract (200.0 g), and *n*-butanol extract (250.0 g) were obtained.

The partial PE extract (80.0 g) was fractionated by silica gel CC (PE–acetone, 20:1 to 0:1, *v*/*v*) to yield ten fractions (PE1–PE10). PE4 (2.8 g) was separated by ODS CC (MeOH − H_2_O, 30:70 to 100:0, *v*/*v*) to obtain four fractions (PE4A–PE4F). PE4F (807.0 mg) was subjected to silica gel CC with a gradient elution of PE/acetone (20:1 to 0:1, *v*/*v*), to afford three fractions (PE4F1–PE4F3). PE4F3 (428.0 mg) was further purified by semi-preparative HPLC (MeCN–H_2_O, 75:25,* v*/*v*, 3 mL/min) to afford **1** (48.0 mg, *t*_R_ 20.0 min) and **5** (50.0 mg, *t*_R_ 26.1 min). PE5 (989.0 mg) was divided into four fractions (PE5A–PE5D) by a Sephadex LH-20 CC (CH_2_Cl_2_–MeOH, 1:1, *v*/*v*). PE5B (195.0 mg) and PE5C (358.0 mg) were chromatographed on an ODS column with a gradient of MeOH/H_2_O (from 50:50 to 100:0, *v*/*v*) to obtain five subfractions, PE5B1–PE5B5 and PE5C1–PE5C5, respectively. PE5B5 (27.0 mg) was further purified by semi-preparative HPLC (MeCN–H_2_O, 50:50, *v*/*v*, 3 mL/min) to yield **18** (3.0 mg, *t*_R_ 25.0 min), **17** (2.0 mg, *t*_R_ 23.0 min), and **22** (2.0 mg, *t*_R_ 39.1 min). PE5C5 (102.0 mg) was then purified by semi-preparative HPLC (MeCN–H_2_O, 85:15,* v*/*v*, 3 mL/min) to yield **11** (18.0 mg, *t*_R_ 16.0 min). PE6 (5.4 g) was similarly separated on a Sephadex LH-20 (CH_2_Cl_2_–MeOH, 1:1, *v*/*v*) to yield three fractions (PE6A–6C). PE6B (3.0 g) was chromatographed on an ODS column (MeOH − H_2_O, 3:7 to 10:0, *v*/*v*) to obtain six subfractions (PE6B1−PE6B6). PE6B5 (2.3 g) yielded a large amount of precipitate (0.71 g), which was purified via semi-preparative HPLC (MeCN–H_2_O, 85:15, 3 mL/min) to obtain compounds **3** (105.0 mg, *t*_R_ 25.0 min) and **6** (10.0 mg, *t*_R_ 29.0 min). PE6B4 (630.0 mg) and the supernatant of PE6B5 (1.6 g) were further separated by ODS CC (MeOH−H_2_O, 50:50 to 100:0, *v*/*v*), both obtaining five sub fractions, PE6B4A−PE6B4E and PE6B5A−PE6B5E. Compound **13** (5.0 mg, *t*_R_ 38.0 min) was obtained from PE6B4E (50.0 mg) by semi-preparative HPLC (MeCN–H_2_O, 52:48, 3 mL/min). Compound **14** (2.0 mg, *t*_R_ 41.3 min) was obtained from PE6B4D (261.0 mg) by semi-preparative HPLC (MeCN–H_2_O, 45:55, *v*/*v*, 3 mL/min). Compound **9** (2.0 mg, *t*_R_ 22.0 min) and **10** (23.0 mg, *t*_R_ 28.0 min) were purified from PE6B5B (69.0 mg) by semi-preparative HPLC (MeCN–H_2_O, 65:35, *v*/*v*, 3 mL/min).

The EA extract (200.0 g) was fractionated by silica gel CC (PE–EA, 99:1 to 0:1, *v*/*v*) to yield ten fractions (EA1–EA10). EA3 (18.0 g), EA4 (32.0 g), and EA5 (48.0 g) were separated by MCI-GEL CHP20P CC and eluted with a stepwise gradient of MeOH/H_2_O (5:95, 30:70, 50:50, 70:30, 90:10 and 100:0, *v*/*v*) to yield four fractions (EA3A–EA3D), five fractions (EA4A–EA4E), and seven fractions (EA5A–EA5G), respectively. EA3A was subjected to a silica gel column eluted with PE/CH_2_Cl_2_ (1:5, 1:7, 1:10 and 1:20, *v/v*) to obtain four fractions (EA3A1–EA3A4). EA3A4 was further fractionated by ODS CC (MeOH–H_2_O, 30:70 to 100:0, *v*/*v*) to yield four fractions (EA3A4A–EA3A4D). EA3A4D was finally purified by semi-preparative HPLC (MeCN–H_2_O, 55:45, *v*/*v*, 3 mL/min) to yield **20** (12.0 mg, *t*_R_ 23.5 min). EA3C was purified by semi-preparative HPLC (MeCN–H_2_O, 78:22,* v*/*v*, 3 mL/min) to afford** 4** (6.0 mg, *t*_R_ 30.7 min) and **7** (9.0 mg, *t*_R_ 36.1 min). EA4D was chromatographed on ODS column (MeOH–H_2_O, 30:70, 50:50, 70:30, 100:0, *v*/*v*) to obtain 14 fractions (EA4D1–EA4D14). EA4D14 was purified by semi-preparative HPLC (MeCN–H_2_O, 88:12, *v*/*v*, 3 mL/min) to yield **2** (13.0 mg, *t*_R_ 23.7 min). EA5E was processed by a Sephadex LH-20 column (CH_2_Cl_2_–MeOH, 1:1, *v*/*v*) to obtain nine subfractions (EA5E1–EA5E9). Compound **12** (3.0 mg, *t*_R_ 25.3 min) was obtained from EA5E7 by semi-preparative HPLC (MeCN/H_2_O, 90:10,* v*/*v*, 3 mL/min). EA6 (800.0 mg) and PE7 (750.0 mg) were subjected to ODS CC with a gradient of MeOH/H_2_O (from 5:95 to 100:0, *v*/*v*), both obtaining ten subfractions, EA6A–EA6J and EA7A–EA7J. Compound **21** (1.3 mg, *t*_R_ 15.2 min) was obtained from EA6H by semi-preparative HPLC (MeCN–H_2_O, 60:40, *v*/*v,* 3 mL/min). Compounds **15** (9.5 mg, *t*_R_ 20.7 min), **16** (2.0 mg, *t*_R_ 22.2 min), and **19** (5.5 mg, *t*_R_ 25.1 min) were purified from EA6I by semi-preparative HPLC (MeCN–H_2_O, 65:35, *v*/*v,* 3 mL/min). EA7H was purified by semi-preparative HPLC (MeCN–H_2_O, 60:40, *v*/*v*,3 mL/min) to yield **8** (10.9 mg, *t*_R_ 20.1 min).

**Aglodorol A (1)**: colorless massive crystals; mp 167–168 °C; $[\alpha]^{20}_{\text{D}}$ + 60.0 (*c* 0.10, MeOH); UV (MeOH) *λ*_max_ (log*ε*) 203 nm; IR (KBr) *ν*_max_ 3320, 2944, 2869, 1643, 1466, 1386, 1377, 1306, 1148, 1044, 1031, 878, 806, 687 cm^− 1^; ^1^H NMR and ^13^C NMR, see Table 1; HRESIMS* m*/*z* 523.3557 [M + HCOO]^−^ (calcd. for C_31_H_52_O_4_Cl, 523.3554).

**Aglodorol B (2)**, colorless needle crystals; mp 165–167 °C; $[\alpha]^{20}_{\text{D}}$ + 30 (*c* 0.10, MeOH); UV (MeOH) *λ*_max_ (log*ε*) 201 nm; IR (KBr) *ν*_max_ 3141, 2942, 2871, 1638, 1436, 1405, 1309, 1146, 1114, 1045, 985, 952, 886, 697, 666 cm^− 1^; ^1^H NMR and ^13^C NMR, see Table 1; HRESIMS *m*/*z* 489.4301 [M + H]^+^ (calcd. for C_32_H_57_O_3_, 489.4308).

**Aglodorol C (8)**, colorless needle crystals; mp 241–242 °C; $[\alpha]^{25}_{\text{D}}$ − 90.0 (*c* 0.10, MeOH); UV (MeOH) *λ*_max_ (log*ε*) 201 nm; IR (KBr) *ν*_max_ 3455, 2938, 2872, 1767, 1724, 1461, 1449, 1374, 1249, 1166, 1098, 1034, 979 cm^− 1^; ^1^H NMR and ^13^C NMR, see Table 1; HRESIMS *m*/*z* 591.3533 [M + HCOO]^−^ (calcd. for C_33_H_51_O_9_, 591.3528).

**Aglodorol D (13)**, colorless massive crystals; mp 152–153 °C; $[\alpha]^{20}_{\text{D}}$ − 10.0 (*c* 0.10, MeOH); UV (MeOH) *λ*_max_ (log*ε*) 201 nm; IR (KBr) *ν*_max_ 3326, 2960, 1705, 1467, 1382, 1238, 1168, 1127, 914, 676 cm^− 1^; ^1^H NMR and ^13^C NMR data, see Table 2; HRESIMS* m*/*z* 305.2480 [M − H]^−^ (calcd. for C_20_H_33_O_2_, 305.2481).

**Aglodorol E (14)***,* colorless needle crystals; mp 145–146 °C; $[\alpha]^{20}_{\text{D}}$ + 60.0 (*c* 0.10, MeOH); UV (MeOH)* λ*_max_ (log*ε*) 193 nm; IR (KBr) *ν*_max_ 3385, 2933, 2858, 1706, 1640, 1441, 1380, 1172, 1134, 1027, 1009, 937, 887, 665 cm^− 1^; ^1^H NMR and ^13^C NMR data, see Table 2; HRESIMS* m*/*z* 305.2480 [M − H]^−^ (calcd. for C_20_H_33_O_2_, 305.2481).

**Aglodorol F (15)**, colorless solid; $[\alpha]^{25}_{\text{D}}$ + 50 (*c* 0.10, MeOH); UV (MeOH) *λ*_max_ (log*ε*) 201 nm; IR (KBr) *ν*_max_ 3405, 2959, 2869, 1717, 1635, 1469, 1450, 1377, 1303, 1126, 937, 885 cm^− 1^; ^1^H NMR and ^13^C NMR, see Table 2; HRESIMS *m*/*z* 351.2539 [M + HCOO]^−^ (calcd. for C_21_H_35_O_4_, 351.2530).

**Aglodorol G (16)**, colorless solid; $[\alpha]^{20}_{\text{D}}$ + 40 (*c* 0.20, MeOH); UV (MeOH) *λ*_max_ (log*ε*) 201 nm; IR (KBr) *ν*_max_ 3089, 2963, 2932, 2872, 1455, 1375 cm^− 1^; ^1^H NMR and ^13^C NMR, see Table 2; HRESIMS *m*/*z* 351.2536 [M + HCOO]^−^ (calcd. for C_21_H_35_O_4_, 351.2541).

**Aglodorol H (19)**, colorless oil; $[\alpha]^{25}_{\text{D}}$ + 10 (*c* 0.10, MeOH); UV (MeOH) *λ*_max_ (log*ε*) 201 nm; IR (KBr) *ν*_max_ 3425, 2938, 2874, 1710, 1663, 1452, 1375, 1172, 1042, 935 cm^− 1^; ^1^H NMR and ^13^C NMR, see Table 2; HRESIMS *m*/*z* 305.2487 [M − H]^−^ (calcd. for C_20_H_33_O_2_, 305.2486).

**Aglodorol I (20)**, light yellow oil; $[\alpha]^{20}_{\text{D}}$ + 100 (*c* 0.10, MeOH); UV (MeOH) *λ*_max_ (log*ε*) 195 nm; IR (KBr) *ν*_max_ 3500, 2933, 1665, 1619, 1443, 1379, 1183, 1088 cm^− 1^; ^1^H NMR and ^13^C NMR, see Table 1; HRESIMS *m*/*z* 303.2327 [M − H]^−^ (calcd. for C_20_H_31_O_2_, 303.2324).

**Aglodorol J (21)**, colorless oil; $[\alpha]^{25}_{\text{D}}$ − 20 (*c* 0.10, MeOH); UV (MeOH) *λ*_max_ (log*ε*) 201 nm; IR (KBr) *ν*_max_ 3396, 3078, 2932, 1640, 1613, 1510, 1454, 1377, 1255, 1148 cm^− 1^; ^1^H NMR and ^13^C NMR, see Table 2; HRESIMS *m*/*z* 303.2325 [M − H]^−^ (calcd. for C_20_H_31_O_2_, 303.2330).

### Crystallographic data and X-ray structure analysis of compounds

Compounds **1**, **2**, **8**, and **13** were obtained as colorless crystals in CHCl_3_, and compound **14** was obtained as colorless crystals in MeOH/H_2_O (1:1, *v*/*v*). The crystallographic data for the structures of these compounds have been deposited in the Cambridge Crystallographic Data Centre (deposition Nos.: CCDC 2441755, 2441756, 2441759, 2092476 and 2092472). These data can be obtained free of charge via www.ccdc.cam.ac.uk/data_request/cif and Table S1.

### Quantum chemistry calculations

The conformation searches of compounds were performed in Spartan 14 with MMFF force field. The primary conformations were then optimized by using DFT method at the B3LYP/6-311G(d) level. The theoretical calculation of NMR was conducted by GIAO method at the mPW1PW91/6-311+G (d, p) in chloroform with PCM model [5]. The calculated NMR data of these conformers were then averaged on the basis of Boltzmann distribution and their relative Gibbs free energies. Subsequent linear regression analysis between experimental and theoretical NMR values yielded a determination coefficient (*R*^2^) [22]. The ECD calculations were performed at B3LYP/6-311+G(d) level in methanol (PCM model). The calculation ECD curves were fitted in SpecDis v1.71 program with 0.3 eV as the half-bandwidth [23]. All theoretical calculations were implemented in Gaussian 16 [24].

### OGD/R model in PC 12 cells

PC 12 cells were plated in 96-well microplates at a density of 7 × 10^3^ cells/well and cultured for 24 h. Following medium removal, the cells underwent gentle PBS washing before exposure to 50 μL EBSS solution containing isolates (10 μM) under anaerobic conditions. After 6 h, each well was replenished with 150 μL complete medium maintaining equivalent drug concentrations for additional 18 h normoxic incubation. The cells in control group were maintained under normoxic conditions throughout the experimental timeline [4]. The cell viability was evaluated by MTT assay, with edaravone (Eda, 16 μM) serving as the positive control.

### LPS-induced neuroinflammatory model in BV-2 cells

BV-2 cells (5 × 10^4^ cells/well) were seeded in 48-well plates and cultured overnight. Subsequently, the cells were incubated with LPS (1 μg/mL) and the tested compounds (20 μM) for 24 h. 50 μL of conditioned medium from each well was collected, mixed with Griess reagent (1:1, *v*/*v*) and monitored at 540 nm to calculate NO release [5]. Concurrent cytotoxicity assessment was performed via MTT assay. Dexamethasone (Dex, 10 μM) served as the positive drug.

### L-glutamate-induced neurotoxicity model in HT22 cells

HT22 cells were plated in 96-well plates at a density of 5 × 10^3^ cells per well and cultured overnight. Following 6 h pre-incubation with the isolates (10 μM), cells were subjected to co-treatment with maintained equivalent compound concentrations and L-glutamic acid (12 mM) in fresh complete medium for 24 h [25]. Cellular viability was quantified via MTT assay.

### RSL3-induced ferroptosis in PC12 cells

PC12 cells were seeded in 96-well microplates at a density of 7 × 10^3^ cells/well and cultured for 24 h. Cells were subsequently exposed to ferroptosis inducer RSL3 (0.4 μM) co-administered with the isolates (10 μM) for 4 h [26]. Cell viability was assessed by MTT assay. The ferroptosis inhibitor Fer-1 (10 μM) served as positive control.

### Statistical analysis

All results were validated by at least three independent experiments and at least three parallel groups were set up in each experiment. The values were expressed as means ± standard deviation (SD). Statistical analyses were conducted using GraphPad Prism 9.0 software and comparisons were made by independent* t*-tests or one-way analysis of variance (ANOVA). A *P*-value of < 0.05 was considered statistically significant throughout the study.

## Supplementary Information

Additional file 1. X-ray crystallographic data of compounds **1**, **2**, **8**, **13**, and **14**; HRESIMS, IR, UV, ECD, 1D, 2D NMR spectra of compounds **1**–**2**, **8**, **13**–**16**, and **19**–**21**; and the experimental and calculated ECD spectra of compounds **17**–**18** and **22**.

## Data Availability

All data generated or analyzed during this study are included in this published article and its supplementary information files.
