# Supplementary material for: Aglodorols A–J, undescribed terpenoids with multidimensional neuroprotective activities from Aglaia odorata Lour
Source: Nat Prod Bioprospect. 2026 Jan 9;16(1):10. doi: 10.1007/s13659-025-00563-2 (PMC12783399; doi:10.1007/s13659-025-00563-2)
Supplement: Supplementary file 1 — Additional file 1. X-ray crystallographic data of compounds 1, 2, 8, 13, and 14; HRESIMS, IR, UV, ECD, 1D, 2D NMR spectra of compounds 1–2, 8, 13–16, and 19–21; and the experimental and calculated ECD spectra of compounds 17–18 and 22. [file 13659_2025_563_MOESM1_ESM.docx]

**Supporting Information**

Aglodorols A–J, undescribed terpenoids with multidimensional neuroprotective activities from *Aglaia odorata* Lour.

Meng Ding, Yuehan Wang, Chenhao Liu, Wangxiao Tan, Liming Hu, Kewu Zeng, Pengfei Tu, Yong Jiang*

*State Key Laboratory of Natural and Biomimetic Drugs, School of Pharmaceutical Sciences, Peking University, Beijing 100191, People’s Republic of China*

These authors have no conflict of interest to declare.

**[* Corresponding author]**

Tel/Fax: +86-10-82802719;

E-mail: [yongjiang@bjmu.edu.cn](mailto:yongjiang@bjmu.edu.cn) (JIANG Yong)

**List of supplementary contents**

The sample preparation of the crude extract of *A*. *odorata*. and LC/MS conditions for the determination of compound **1** and **2**

The sample preparation of the crude extract of *A*. *odorata*. and LC/MS conditions for the determination of compound **2**

**Table S1** X-ray crystallographic data of compounds **1**, **2**, **8**, **13**, and **14**

**Figure S1** HRESIMS of compound **1**

**Figure S2** IR spectrum of compound **1**

**Figure S3** UV spectrum of compound **1**

**Figure S4** ECD spectrum of compound **1**

**Figure S5** ^1^H-NMR spectrum of compound **1** (500 MHz, chloroform-*d*_1_)

**Figure S6** ^13^C-NMR spectrum of compound **1** (125 MHz, chloroform-*d*_1_)

**Figure S7** HSQC spectrum of compound **1**

**Figure S8** HMBC spectrum of compound **1**

**Figure S9** ^1^H-^1^H COSY spectrum of compound **1**

**Figure S10** NOESY spectrum of compound **1**

**Figure S11** LC/MS chromatograms of detection of **1** in the crude extract of *A*. *odorata*.

**Figure S12** HRESIMS of compound **2**

**Figure S13** IR spectrum of compound **2**

**Figure S14** UV spectrum of compound **2**

**Figure S15** ECD spectrum of compound **2**

**Figure S16** ^1^H-NMR spectrum of compound **2** (500 MHz, chloroform-*d*_1_)

**Figure S17** ^13^C-NMR spectrum of compound **2** (125 MHz, chloroform-*d*_1_)

**Figure S18** HSQC spectrum of compound **2**

**Figure S19** HMBC spectrum of compound **2**

**Figure S20** ^1^H-^1^H COSY spectrum of compound **2**

**Figure S21** NOESY spectrum of compound **2**

**Figure S22** LC/MS chromatograms of detection of **2** in the crude extract of *A*. *odorata*.

**Figure S23** HRESIMS of compound **8**

**Figure S24** IR spectrum of compound **8**

**Figure S25** UV spectrum of compound **8**

**Figure S26** ECD spectrum of compound **8**

**Figure S27** ^1^H-NMR spectrum of compound **8** (500 MHz, chloroform-*d*_1_)

**Figure S28** ^13^C-NMR spectrum of compound **8** (125 MHz, chloroform-*d*_1_)

**Figure S29** HSQC spectrum of compound **8**

**Figure S30** HMBC spectrum of compound **8**

**Figure S31** ^1^H-^1^H COSY spectrum of compound **8**

**Figure S32** NOESY spectrum of compound **8**

**Figure S33** HRESIMS of compound **13**

**Figure S34** IR spectrum of compound **13**

**Figure S35** UV spectrum of compound **13**

**Figure S36** ECD spectrum of compound **13**

**Figure S37** ^1^H-NMR spectrum of compound **13** (500 MHz, chloroform-*d*_1_)

**Figure S38** ^13^C-NMR spectrum of compound **13** (125 MHz, chloroform-*d*_1_)

**Figure S39** HSQC spectrum of compound **13**

**Figure S40** HMBC spectrum of compound **13**

**Figure S41** ^1^H-^1^H COSY spectrum of compound **13**

**Figure S42** NOESY spectrum of compound **13**

**Figure S43** HRESIMS of compound **14**

**Figure S44** IR spectrum of compound **14**

**Figure S45** UV spectrum of compound **14**

**Figure S46** ECD spectrum of compound **14**

**Figure S47** ^1^H-NMR spectrum of compound **14** (500 MHz, chloroform-*d*_1_)

**Figure S48** ^13^C-NMR spectrum of compound **14** (125 MHz, chloroform-*d*_1_)

**Figure S49** HSQC spectrum of compound **14**

**Figure S50** HSQC spectrum of compound **14**

**Figure S51** ^1^H-^1^H COSY spectrum of compound **14**

**Figure S52** NOESY spectrum of compound **14**

**Figure S53** HRESIMS of compound **15**

**Figure S54** IR spectrum of compound **15**

**Figure S55** UV spectrum of compound **15**

**Figure S56** ^1^H-NMR spectrum of compound **15** (500 MHz, chloroform-*d*_1_)

**Figure S57** ^13^C-NMR spectrum of compound **15** (125 MHz, chloroform-*d*_1_)

**Figure S58** HSQC spectrum of compound **15**

**Figure S59** HMBC spectrum of compound **15**

**Figure S60** ^1^H-^1^H COSY spectrum of compound **15**

**Figure S61** NOESY spectrum of compound **15**

**Figure S62** HRESIMS of compound **16**

**Figure S63** IR spectrum of compound **16**

**Figure S64** UV spectrum of compound **16**

**Figure S65** ^1^H-NMR spectrum of compound **16** (500 MHz, chloroform-*d*_1_)

**Figure S66** ^13^C-NMR spectrum of compound **16** (125 MHz, chloroform-*d*_1_)

**Figure S67** HSQC spectrum of compound **16**

**Figure S68** HMBC spectrum of compound **16**

**Figure S69** ^1^H-^1^H COSY spectrum of compound **16**

**Figure S70** NOESY spectrum of compound **16**

**Figure S71** HRESIMS of compound **19**

**Figure S72** IR spectrum of compound **19**

**Figure S73** UV spectrum of compound **19**

**Figure S74** ^1^H-NMR spectrum of compound **19** (500 MHz, chloroform-*d*_1_)

**Figure S75** ^13^C-NMR spectrum of compound **19** (125 MHz, chloroform-*d*_1_)

**Figure S76** HSQC spectrum of compound **19**

**Figure S77** HMBC spectrum of compound **19**

**Figure S78** ^1^H-^1^H COSY spectrum of compound **19**

**Figure S79** NOESY spectrum of compound **19**

**Figure S80** HRESIMS of compound **20**

**Figure S81** IR spectrum of compound **20**

**Figure S82** UV spectrum of compound **20**

**Figure S83** ^1^H-NMR spectrum of compound **20** (500 MHz, chloroform-*d*_1_)

**Figure S84** ^13^C-NMR spectrum of compound **20** (125 MHz, chloroform-*d*_1_)

**Figure S85** HSQC spectrum of compound **20**

**Figure S86** HMBC spectrum of compound **20**

**Figure S87** ^1^H-^1^H COSY spectrum of compound **20**

**Figure S88** NOESY spectrum of compound **20**

**Figure S89** HRESIMS of compound **21**

**Figure S90** IR spectrum of compound **21**

**Figure S91** UV spectrum of compound **21**

**Figure S92** ^1^H-NMR spectrum of compound **21** (500 MHz, chloroform-*d*_1_)

**Figure S93** ^13^C-NMR spectrum of compound **21** (125 MHz, chloroform-*d*_1_)

**Figure S94** HSQC spectrum of compound **21**

**Figure S95** HMBC spectrum of compound **21**

**Figure S96** ^1^H-^1^H COSY spectrum of compound **21**

**Figure S97** NOESY spectrum of compound **21**

**Figure S98** The experimental and calculated ECD spectra of compounds **17**–**18**, and **22**.

The sample preparation of the crude extract of *A*. *odorata*. and LC/MS conditions for the determination of compound **1**.

An aliquot of *A*. *odorata* powder (0.6 g) was accurately weighed and subjected to ultrasonic extraction with 95% ethanol (30 mL) for 30 min. The extract was centrifuged at 12,000 rpm for 20 min to afford a supernatant, which was collected and used as the sample solution. A standard solution of compound **1** was prepared in methanol at a concentration of 1 mg/mL. Chromatographic separation and detection were achieved using an ACQUITY H–Class UPLC system (Waters, Milford, MA, USA) coupled with a QTRAP 4500 triple quadrupole mass spectrometer (Sciex, Framingham, MA, USA). The mobile phaseconsisted of water (A) and acetonitrile (B), delivered according to the following gradient program: 0–10 min, 30% to 100% B; 10–17 min, 100% B. The flow rate was 0.6 mL/min and the column temperature was maintained at 30 °C. Mass spectrometric detection was performed in multiple reaction monitoring (MRM) mode under negative electrospray ionization with the ion transition set at *m*/*z* 523/255. The optimized MS parameters were as follows: ion spray voltage: negative ion −5500 V; curtain gas: 35 psi; ion source temperature: 550°C; nebulizer gas (GS 1): 55 psi, and heater gas (GS 2): 55 psi. Data acquisition and processing were carried out using Analyst 1.7.2 software (Sciex, Framingham, MA, USA).

The sample preparation of the crude extract of *A*. *odorata*. and LC/MS conditions for the determination of compound **2**

An aliquot of *A*. *odorata* powder (0.6 g) was accurately weighed and subjected to ultrasonic extraction with methanol (30 mL) for 30 min. The extract was centrifuged at 12,000 rpm for 20 min to afford a supernatant, which was collected and used as the sample solution. A standard solution of compound **2** was prepared in methanol at a concentration of 9.78 μg/mL. Chromatographic separation and detection were achieved using an ACQUITY H–Class UPLC system (Waters, Milford, MA, USA) coupled with a QTRAP 4500 triple quadrupole mass spectrometer (Sciex, Framingham, MA, USA). The mobile phase consisted of 10 mM ammonium acetate in water (A) and 10 mM ammonium acetate in acetonitrile-water (90:10, *v*/*v*) (B), delivered according to the following gradient program: 0–1 min, 40% B; 1–8 min, 40%–100% B; 8–14 min, 100% B. The flow rate was 0.25 mL/min and the column temperature was maintained at 40 °C. Mass spectrometric detection was performed in multiple reaction monitoring (MRM) mode under positive electrospray ionization with the ion transition set at *m*/*z* 506.5/425.2, 506.5/443.4, 506.5/191.1. The optimized MS parameters were as follows: ion spray voltage: positive ion 5500 V; curtain gas: 40 psi; ion source temperature: 550°C; nebulizer gas (GS 1): 55 psi, and heater gas (GS 2): 55 psi. Data acquisition and processing were carried out using Analyst 1.7.2 software (Sciex, Framingham, MA, USA).

**Table S1**. X-ray crystallographic data of compounds **1**, **2**, **8**, **13**, and **14**

| Parameters | **1** ^a^ | **2** ^a^ | **8** ^a^ | **13** ^a^ | **14** ^b^ |
| --- | --- | --- | --- | --- | --- |
| Empirical formula | C_30_H_55_O_3_Cl | C_33_H_60_O_4_ | C_32_H_51.33_O_7.67_ | C_20_H_34_O_2_ | C_20_H_33_O_2.5_ |
| Formula weight | 511.20 | 520.81 | 558.74 | 306.47 | 313.46 |
| Temperature | 100 K | 100 K | 100 K | 100 K | 100 K |
| Crystal system | orthorhombic | orthorhombic | orthorhombic | orthorhombic | monoclinic |
| Space group | P2_1_2_1_2_1_ | P2_1_2_1_2_1_ | P2_1_2_1_2 | P2_1_2_1_2_1_ | I2 |
| Unit cell dimensions | *a=* 8.38650(10) Å, α = 90° | *a* = 6.85295(19) Å, α = 90° | *a=* 23.9006(9) Å, α = 90° | *a* = 11.7030(2) Å, α = 90° | *a* = 16.3683(4) Å, α = 90° |
|  | *b=* 11.21260(10) Å, β = 90° | *b* = 13.1455(5) Å, β = 90° | *b=* 17.2190(6) Å, β = 90° | *b* = 17.6423(3) Å, β = 90° | *b* = 6.04530(10) Å, β = 110.758(3)° |
|  | *c=* 31.0510(3)Å, γ = 90° | *c* = 34.4861(10) Å, γ = 90° | *c=* 7.1835(3) Å, γ = 90° | *c* = 17.7951(3) Å, γ = 90° | *c* = 19.3431(4) Å, γ = 90° |
| Volume | 2919.86(5)Å^3^ | 3106.71(17) Å^3^ | 2956.3(2) Å^3^ | 3674.11(11) Å^3^ | 1789.78(7) Å^3^ |
| Z | 4 | 4 | 4 | 8 | 4 |
| Calculated density | 1.163 g/cm^3^ | 1.113 g/cm^3^ | 1.255 g/cm^3^ | 1.108 g/cm^3^ | 1.163 g/cm^3^ |
| Absorption coefficient | 1.366 μ/mm^‑1^ | 0.544 μ/mm^‑1^ | 0.709μ/mm^-1^ | 0.529 μ/mm^‑1^ | 0.577 μ/mm^‑1^ |
| F (000) | 1128.0 | 1160.0 | 1219.0 | 1360 | 692 |
| Crystal size | 0.35 × 0.2 × 0.15 mm^3^ | 0.35 × 0.03 × 0.02 mm^3^ | 0.08 × 0.03 × 0.02 mm^3^ | 0.35 × 0.21 × 0.12 mm^3^ | 0.48 × 0.08 × 0.02 mm^3^ |
| Theta range for data collection | 8.384 to 150.164° | 5.124 to 155.808° | 7.398 to 155.754° | 7.056 to 150.444° | 8.792 to 140.148° |
| Index ranges | −10 ≤ h ≤ 10, −14 ≤ k ≤ 12, −36 ≤ l ≤ 38 | −8 ≤ h ≤ 6, −15 ≤ k ≤ 16, −43 ≤ l ≤ 42 | −30 ≤ h ≤ 26, −20 ≤ k ≤ 21, −8 ≤ l ≤ 8 | −14 ≤ h ≤ 12, −20 ≤ k ≤ 22, −21 ≤ l ≤ 22 | −17 ≤ h ≤ 19, −7 ≤ k ≤ 7, −23 ≤ l ≤ 19 |
| Reflections collected | 17659 | 38284 | 27096 | 30613 | 13693 |
| Independent reflections | 5814 [R_int_ = 0.0500, R_sigma_ = 0.0376] | 6126 [R_int_ = 0.0822, R_sigma_ = 0.0423] | 6043 [R_int_ = 0.0505, R_sigma_ = 0.0343] | 7306 [R_int_ = 0.1267, R_sigma_ = 0.0759] | 3327 [R_int_ = 0.0655, R_sigma_ = 0.0382] |
| Data / restraints / parameters | 5814/0/327 | 6126/0/346 | 6043/114/478 | 7306/0/ 411 | 3327/1/213 |
| Goodness−of−fit on F^2^ | 1.022 | 1.075 | 1.078 | 1.120 | 1.064 |
| Final R indices [I˃2*σ*(I)] | R_1_ = 0.0376, wR_2_ = 0.0994 | R_1_ = 0.0570, wR_2_ = 0.1552 | R_1_ = 0.0685, wR_2_ = 0.1727 | R_1_ = 0.0707, wR_2_ = 0.1847 | R_1_ = 0.0456, wR_2_ = 0.1233 |
| R indices (all data) | R_1_ = 0.0384, wR_2_ = 0.1000 | R_1_ = 0.0707, wR_2_ = 0.1643 | R_1_ = 0.0826, wR_2_ = 0.1812 | R_1_ = 0.0800, wR_2_ = 0.2014 | R_1_ = 0.0477, wR_2_ = 0.1294 |
| Largest diff. peak and hole | 0.22/−0.20 e. Å^3^ | 0.44/−0.23 e. Å^3^ | 0.24/−0.23 e. Å^3^ | 0.42/−0.34 e. Å^3^ | 0.60/-0.26 e. Å^3^ |
| Flack parameter | 0.003(9) | −0.09(10) | 0.13(15) | 0.0(2) | 0.03(16) |
| NOs. CCDC | 2441755 | 2441756 | 2441759 | 2092476 | 2092472 |

^a^ Colorless crystals of compounds **1**, **2**, **8**, and **13** were obtained in CHCl_3_

^b^ Colorless crystals of compound **14** were obtained in methanol/H_2_O (1:1, *v*/*v*)


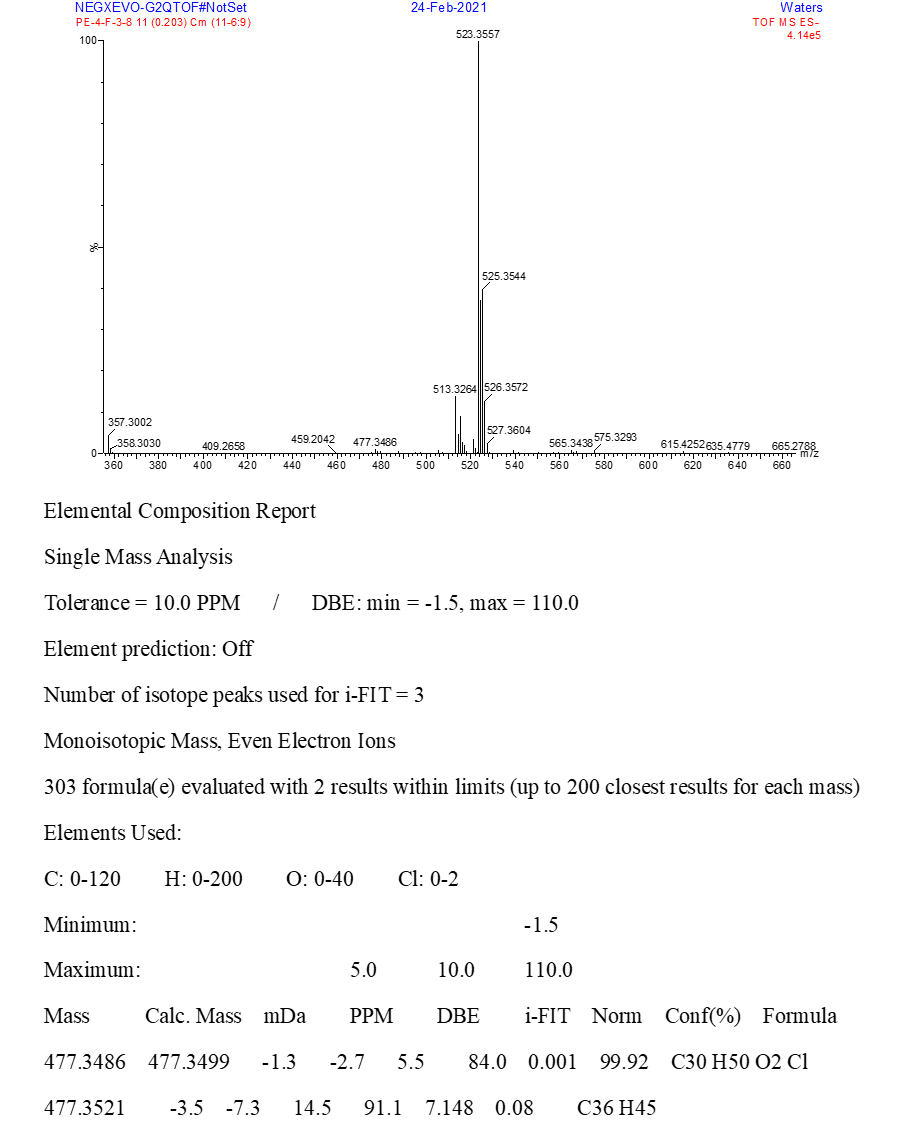


**Figure S1** HRESIMS of compound **1**


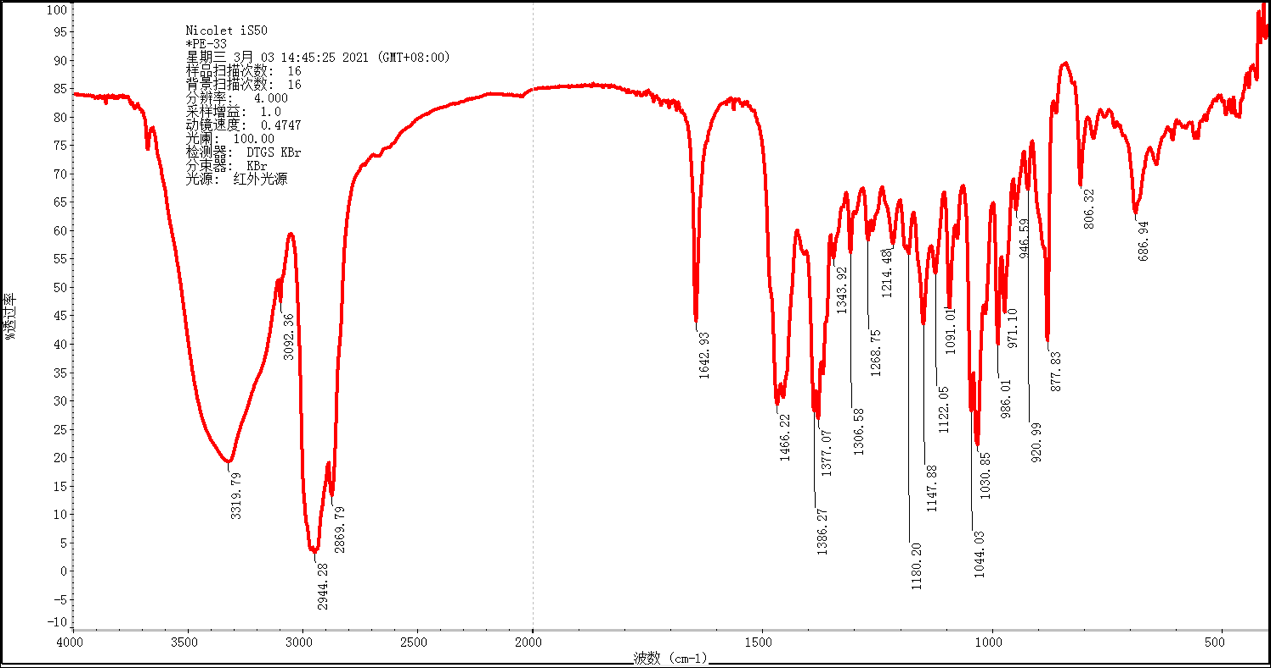


**Figure S2** IR spectrum of compound **1**

**
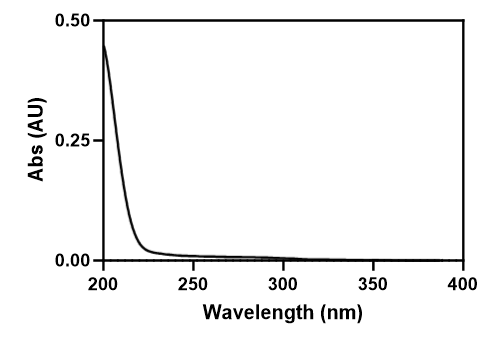
**

**Figure S3** UV spectrum of compound **1**


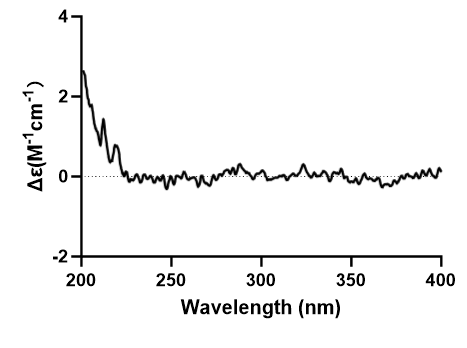


**Figure S4** ECD spectrum of compound **1**


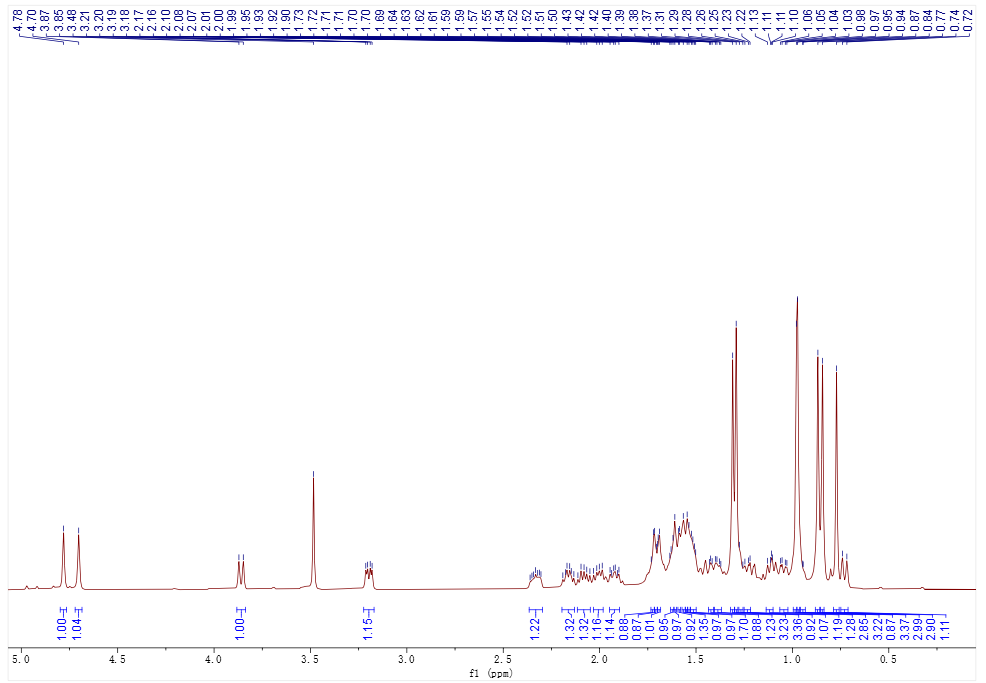


**Figure S5** ^1^H-NMR spectrum of compound **1** (500 MHz, chloroform-*d*_1_)


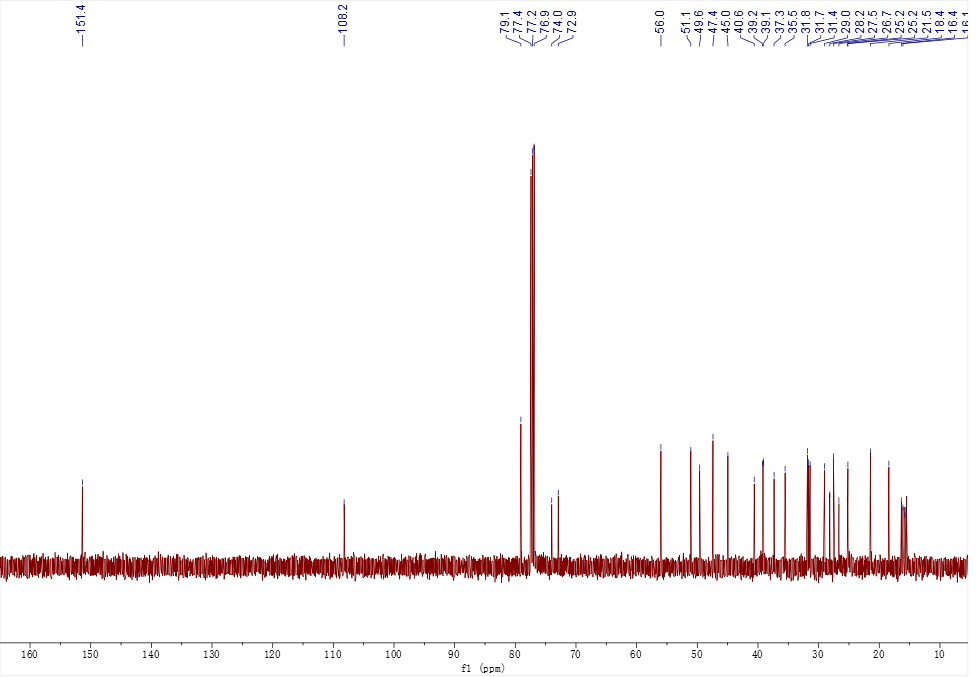


**Figure S6** ^13^C-NMR spectrum of compound **1** (125 MHz, chloroform-*d*_1_)

**
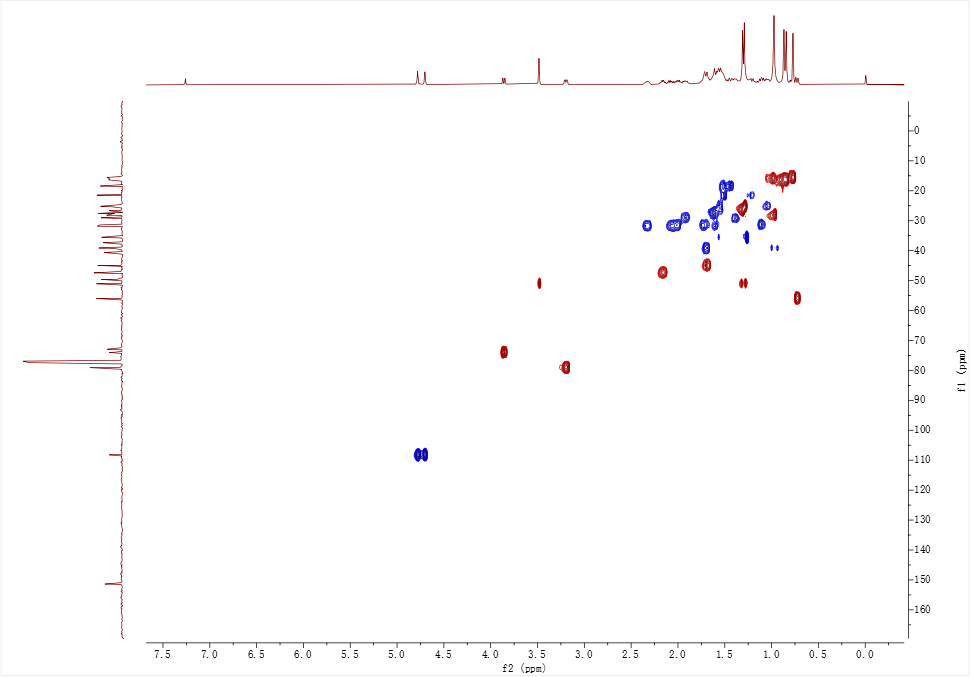
**

**Figure S7** HSQC spectrum of compound **1**

**
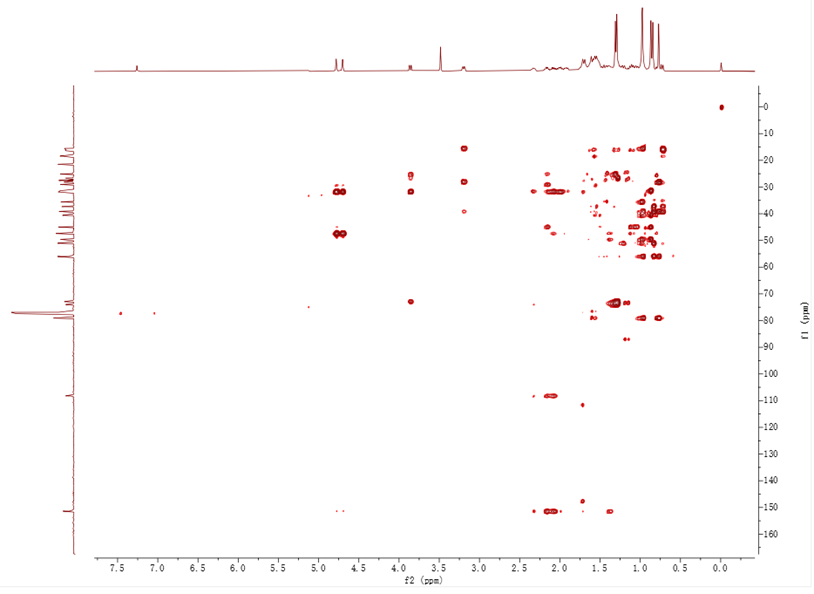
**

**Figure S8** HMBC spectrum of compound **1**


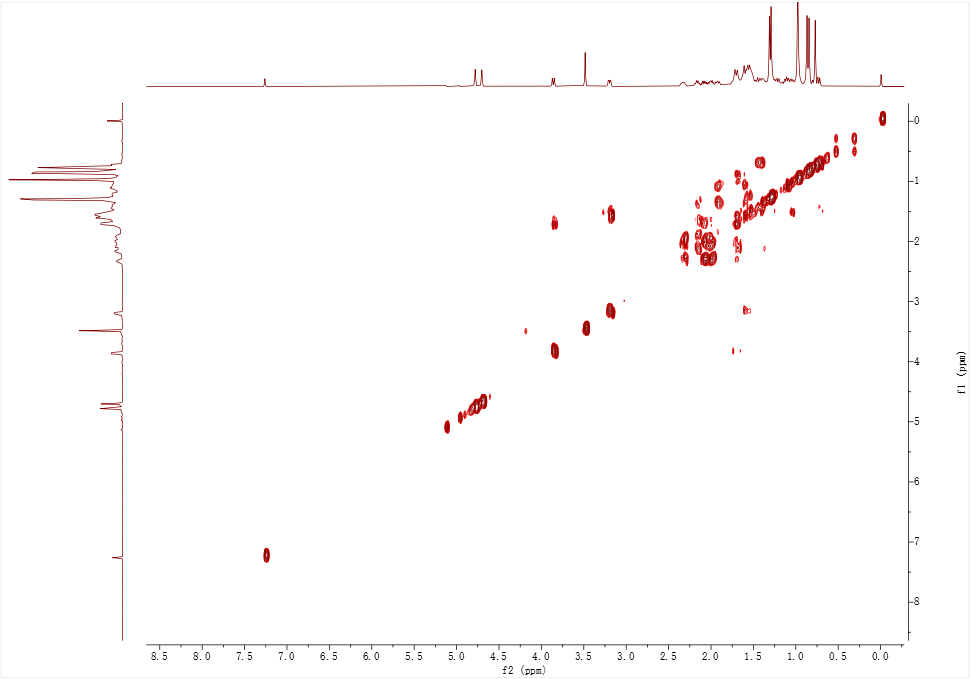


**Figure S9** ^1^H-^1^H COSY spectrum of compound **1**

**
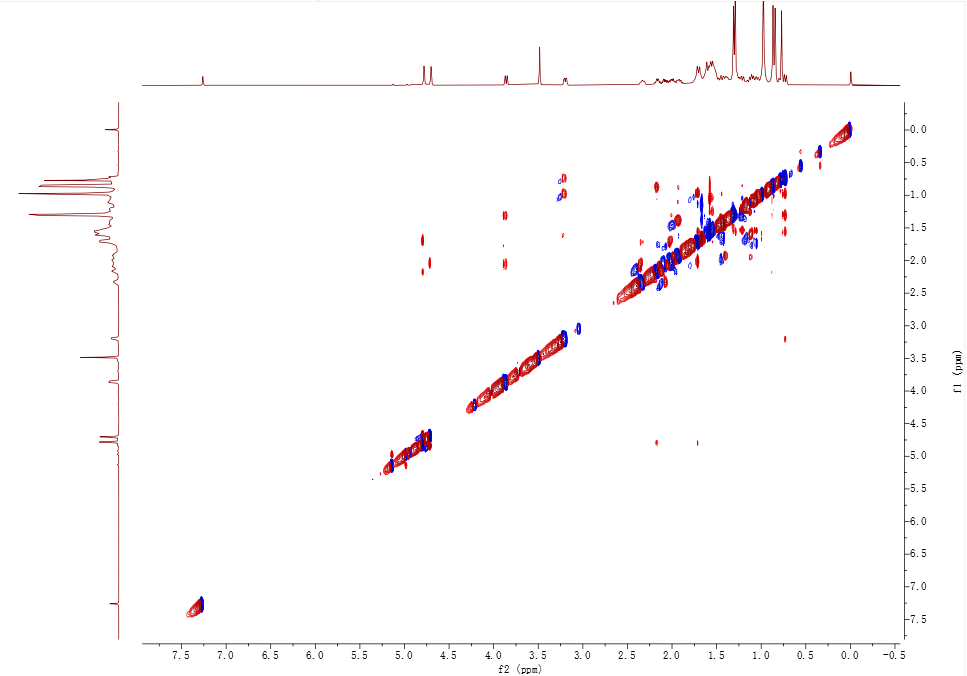
**

**Figure S10** NOESY spectrum of compound **1**


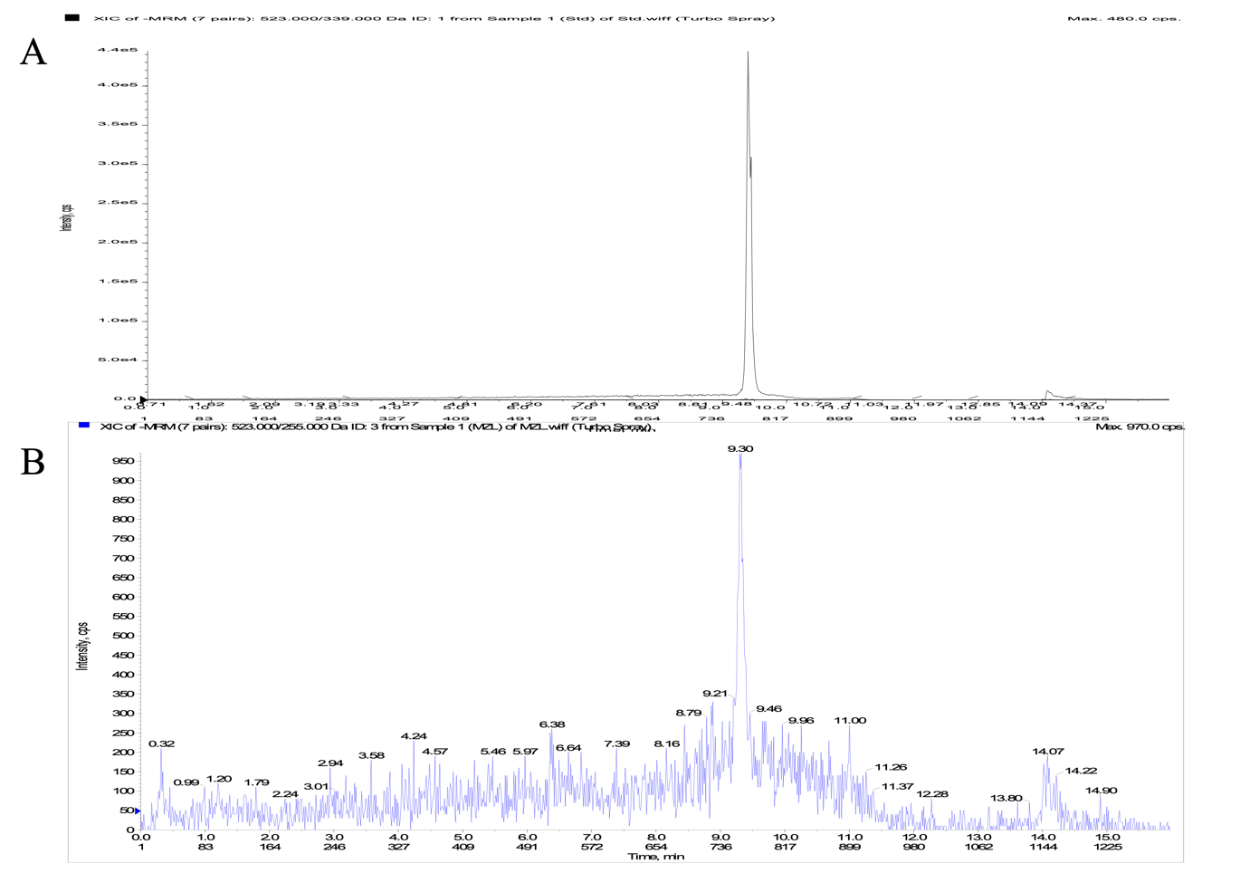


**Figure S11** LC/MS chromatograms of detection of **1** in the crude extract of *A*. *odorata*. (A: EIC of **1**; B: XIC of **1** (*m*/*z*: 523/255) from the crude extract of *A*. *odorata*. under the MRM mode)

**Figure S12** HRESIMS of compound **2**


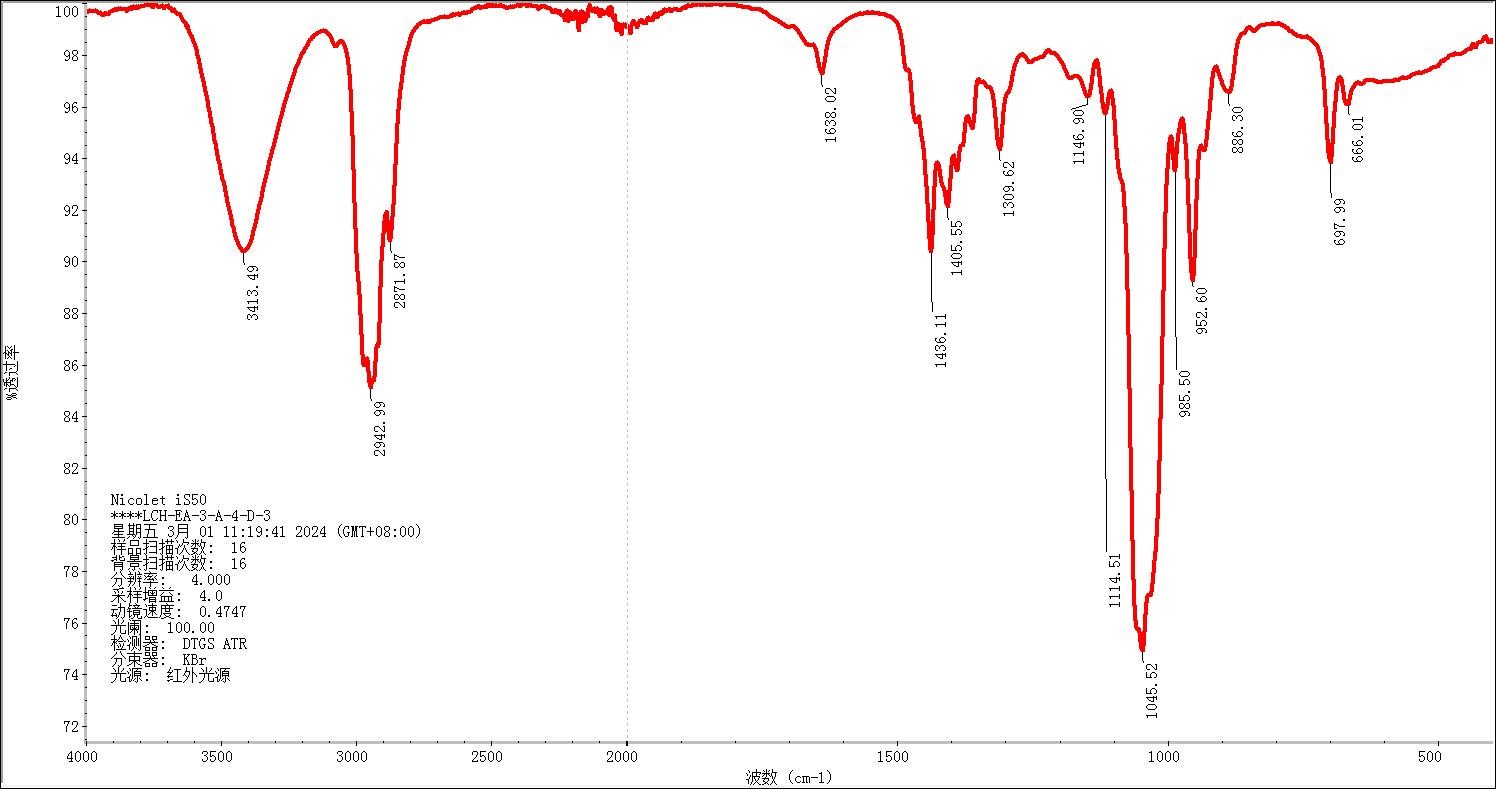


**Figure S13** IR spectrum of compound **2**

**
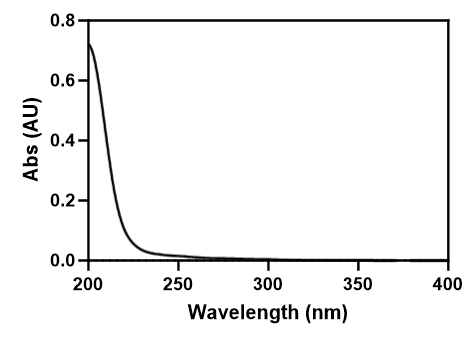
**

**Figure S14** UV spectrum of compound **2**

**
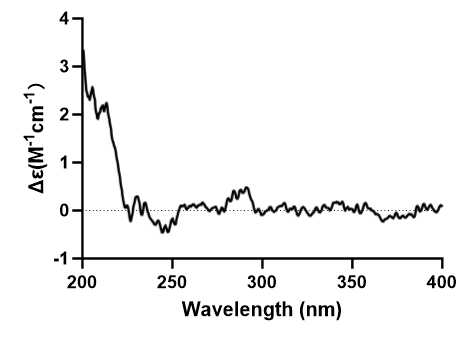
**

**Figure S15** ECD spectrum of compound **2**


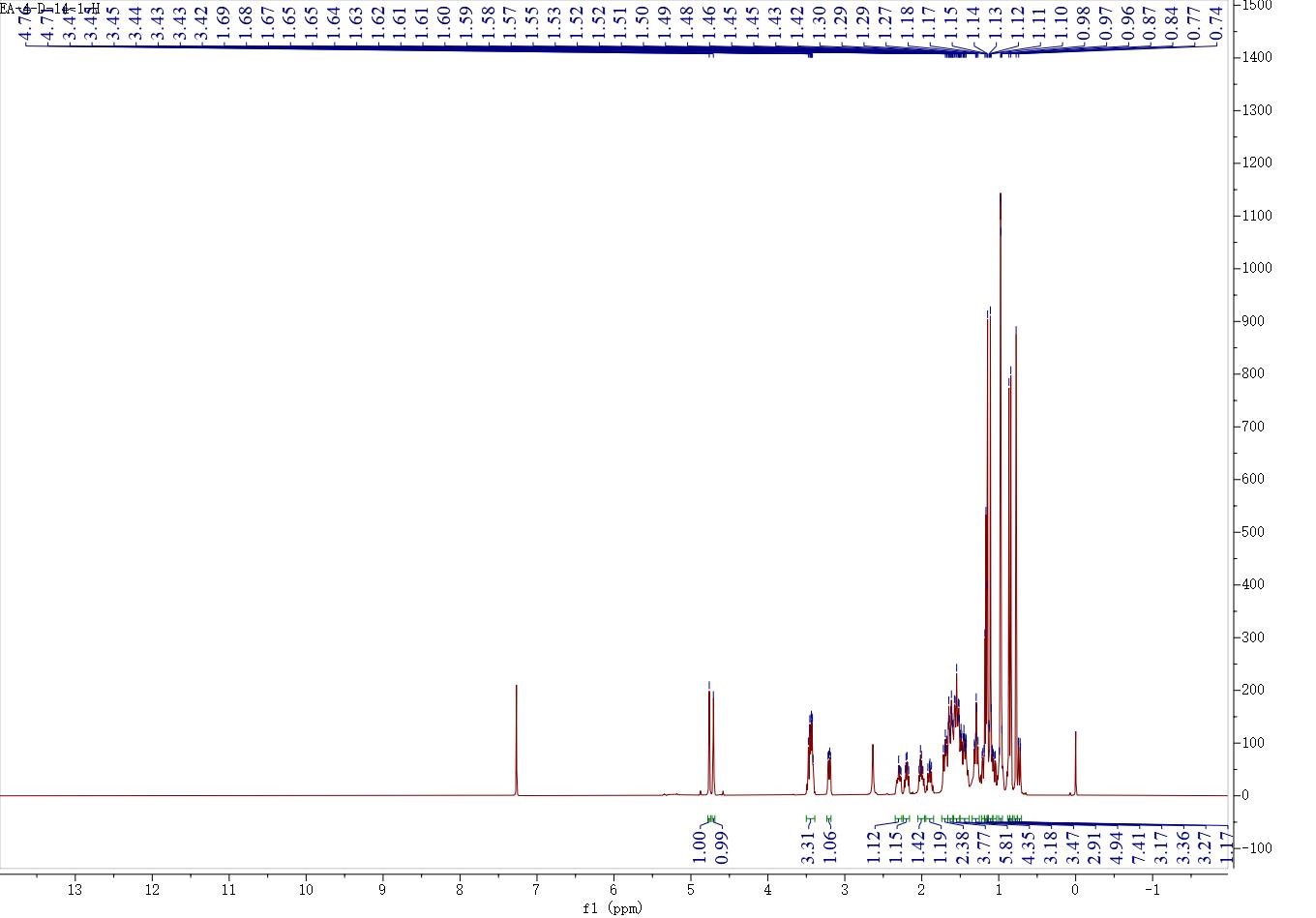


**Figure S16** ^1^H-NMR spectrum of compound **2** (500 MHz, chloroform-*d*_1_)


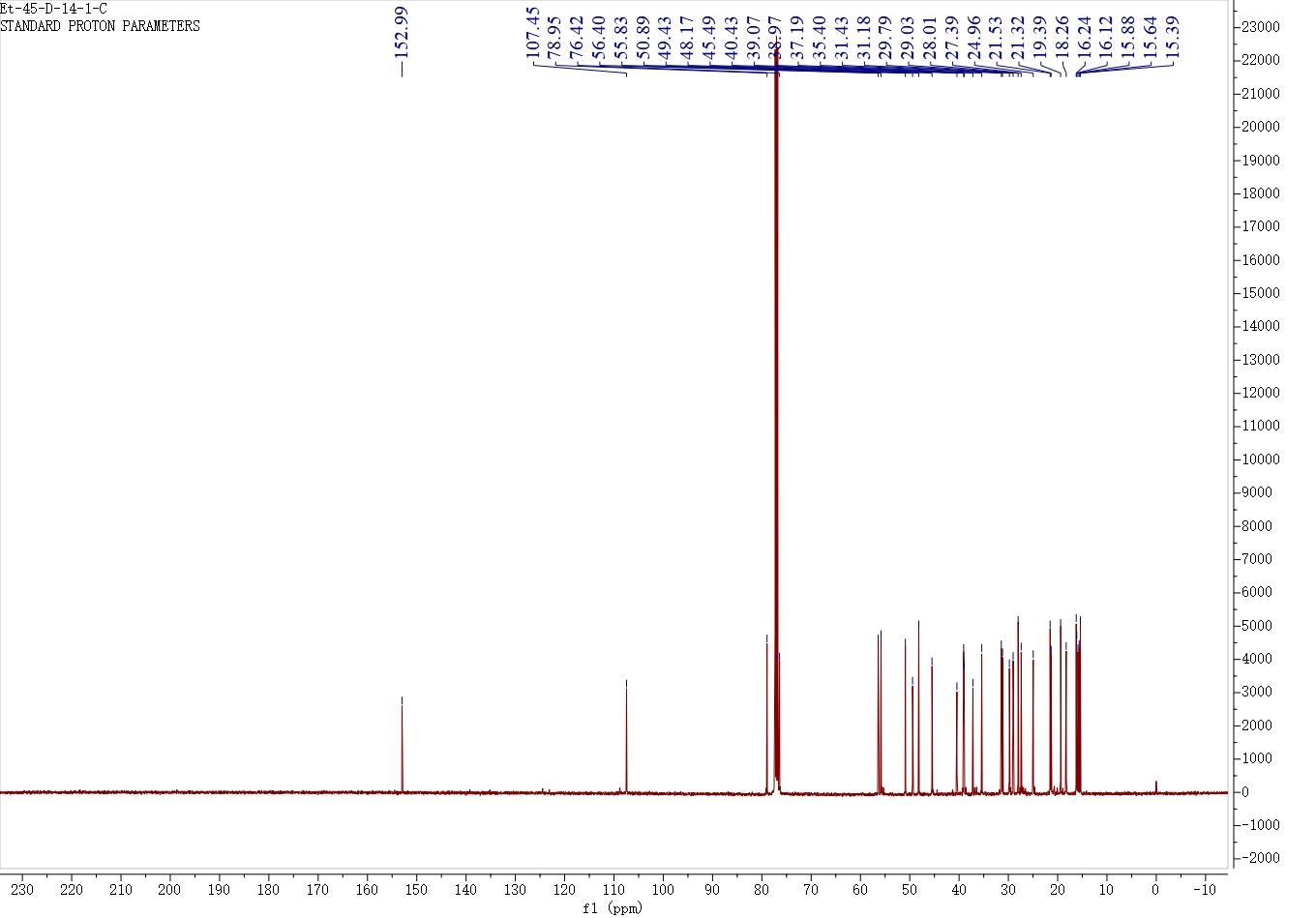


**Figure S17** ^13^C-NMR spectrum of compound **2** (125 MHz, chloroform-*d*_1_)


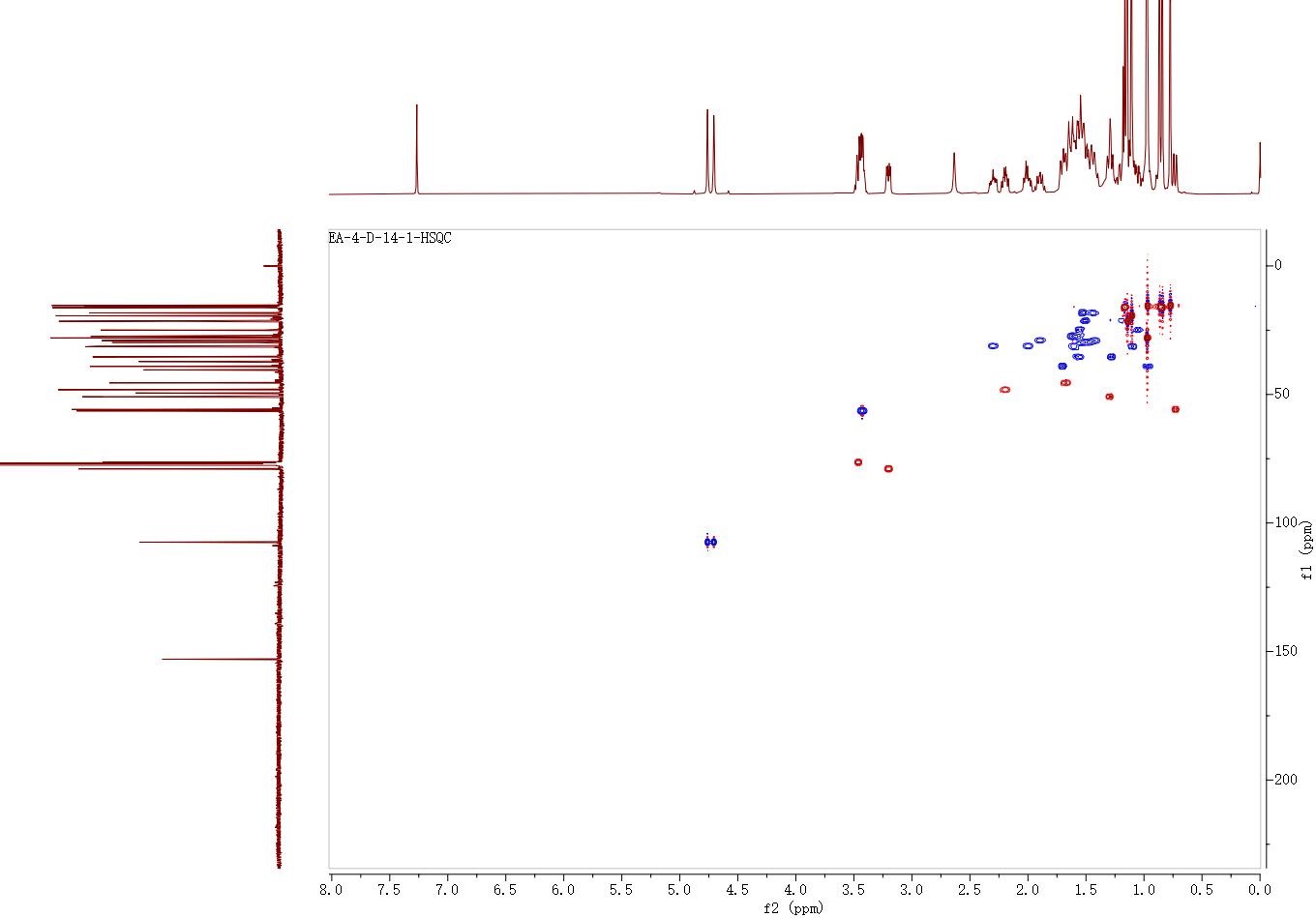


**Figure S18** HSQC spectrum of compound **2**


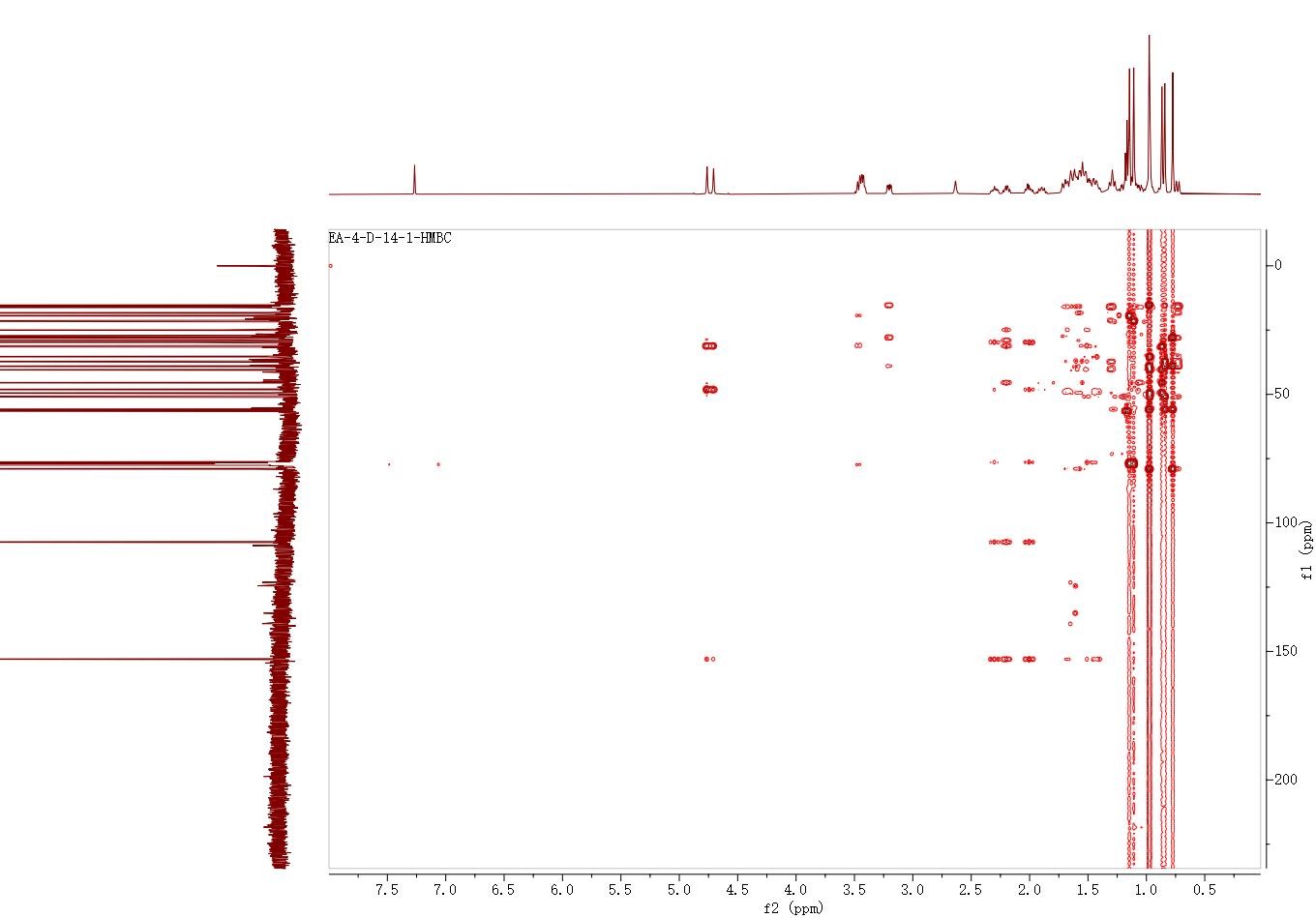


**Figure S19** HMBC spectrum of compound **2**


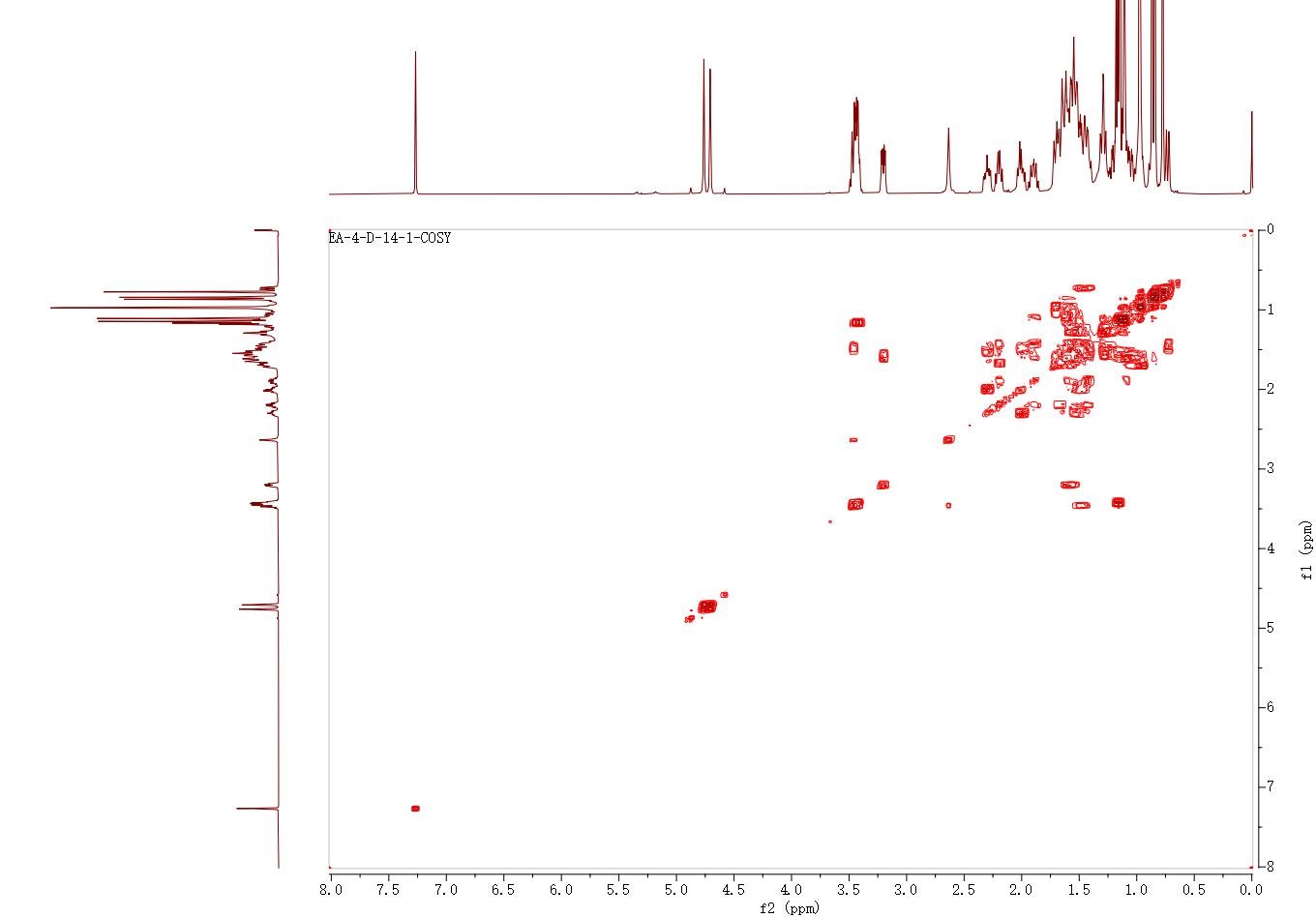


**Figure S20** ^1^H-^1^H COSY spectrum of compound **2**


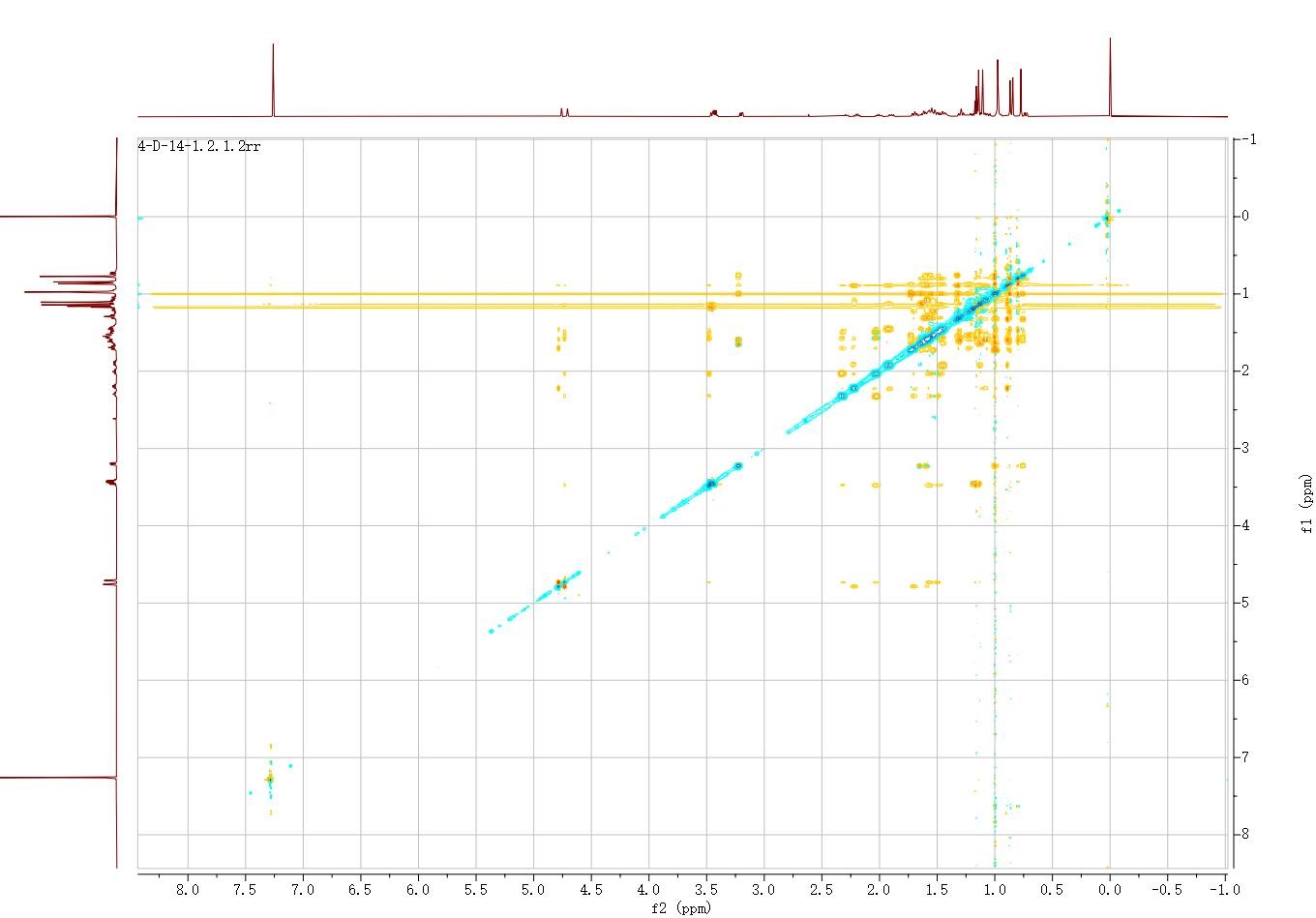


**Figure S21** NOESY spectrum of compound **2**

**Figure S22** LC/MS chromatograms of detection of **2** in the crude extract of *A*. *odorata*.

**Figure S23** HRESIMS of compound **8**


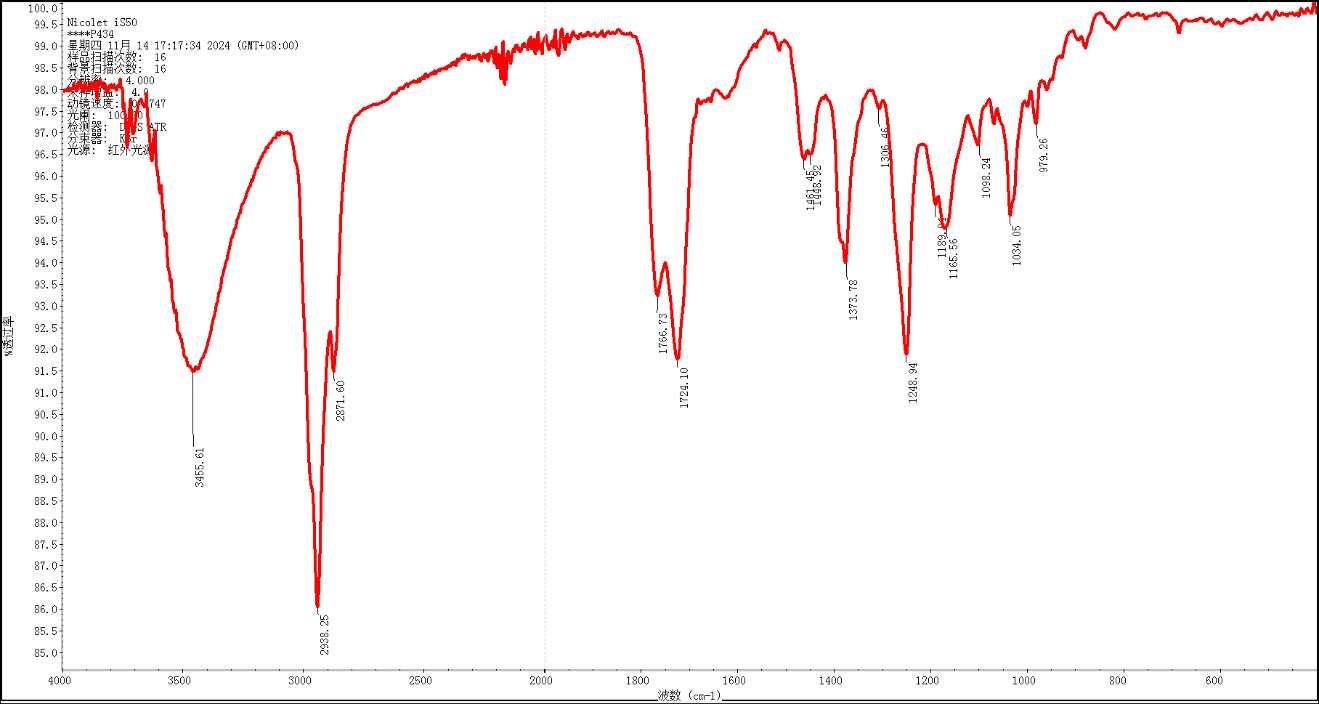


**Figure S24** IR spectrum of compound **8**


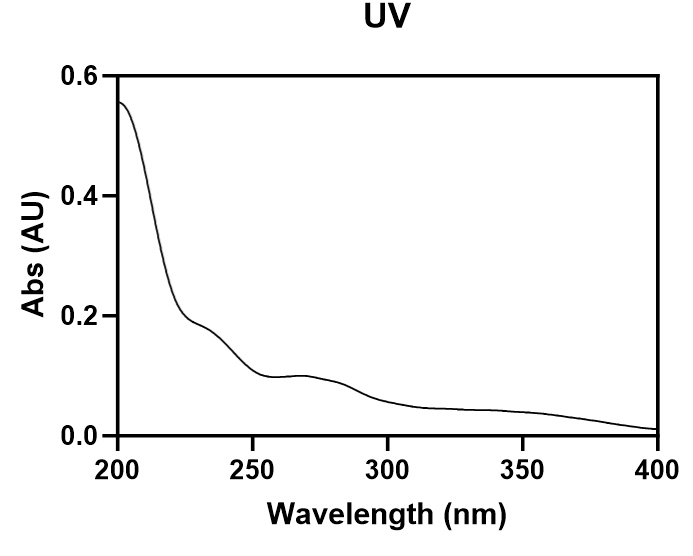
**
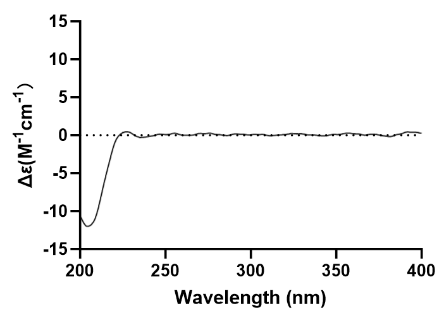
Figure S25** UV spectrum of compound **8**

**Figure S26** ECD spectrum of compound **8**

**
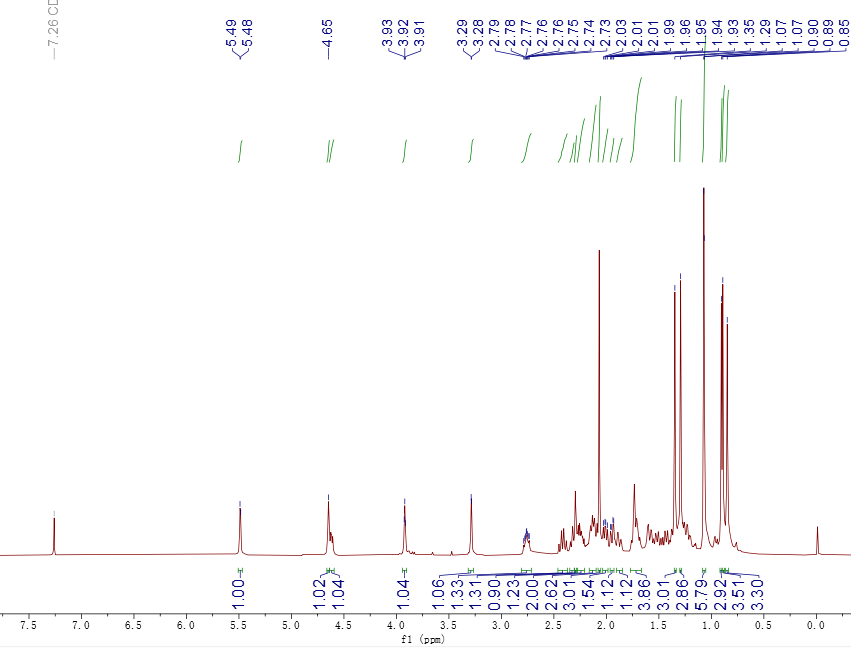
**

**Figure S27** ^1^H-NMR spectrum of compound **8** (500 MHz, chloroform-*d*_1_)


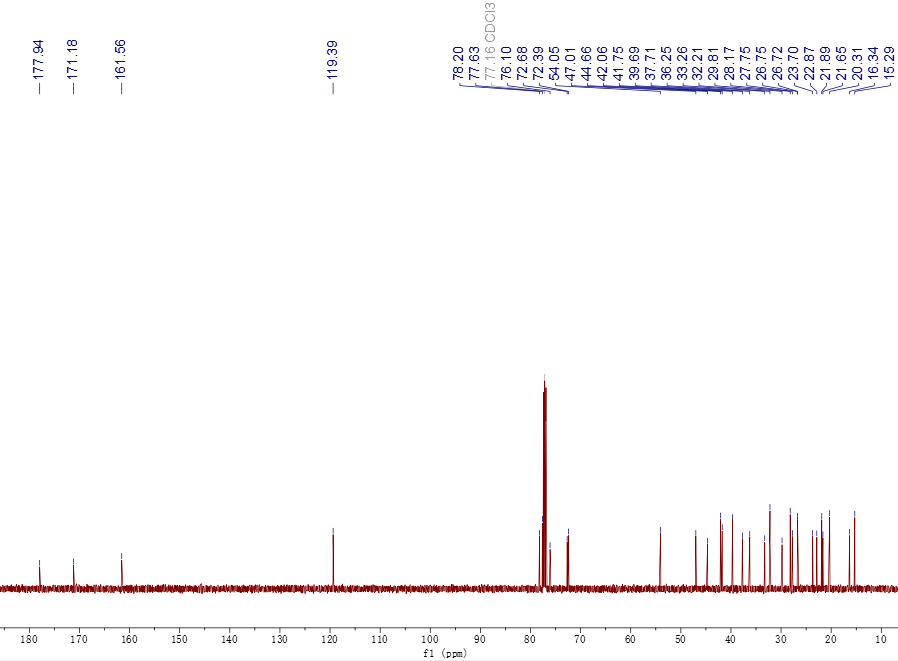


**Figure S28** ^13^C-NMR spectrum of compound **8** (125 MHz, chloroform-*d*_1_)

**
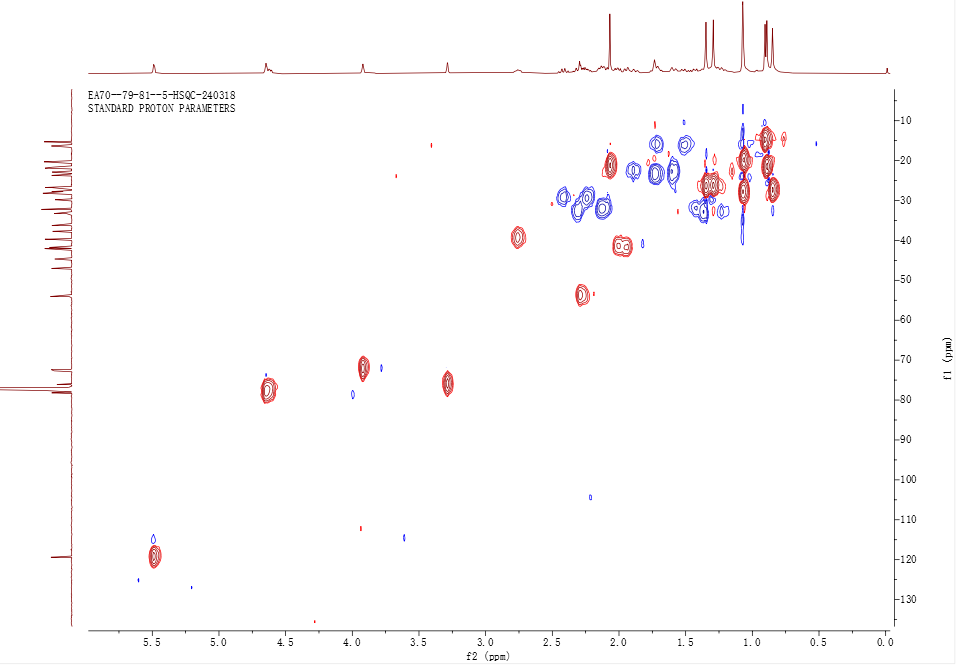
**

**Figure S29** HSQC spectrum of compound **8**

**
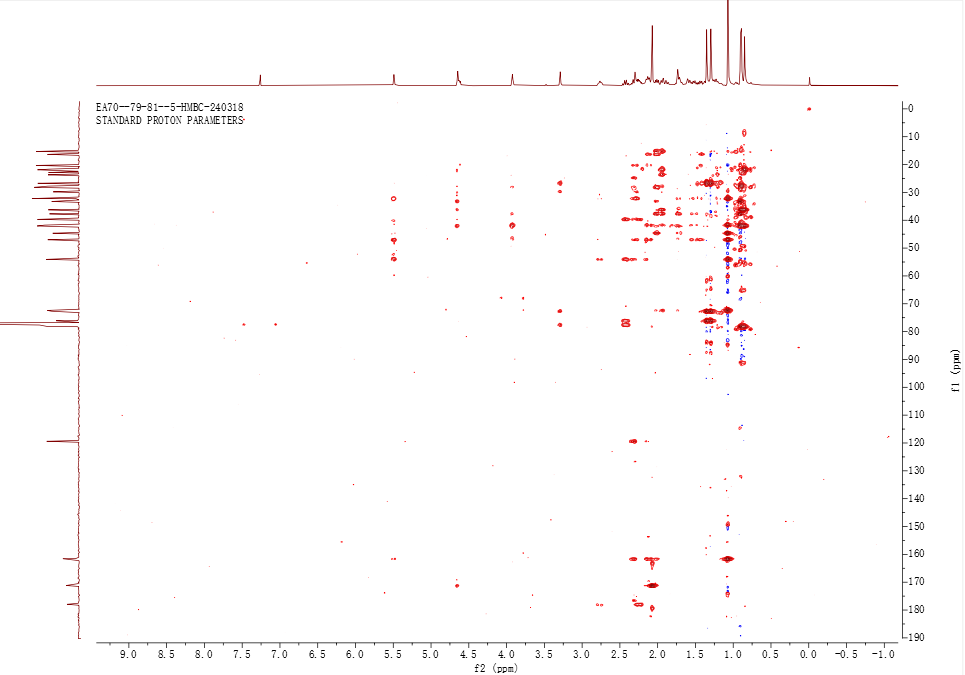
**

**Figure S30** HMBC spectrum of compound **8**


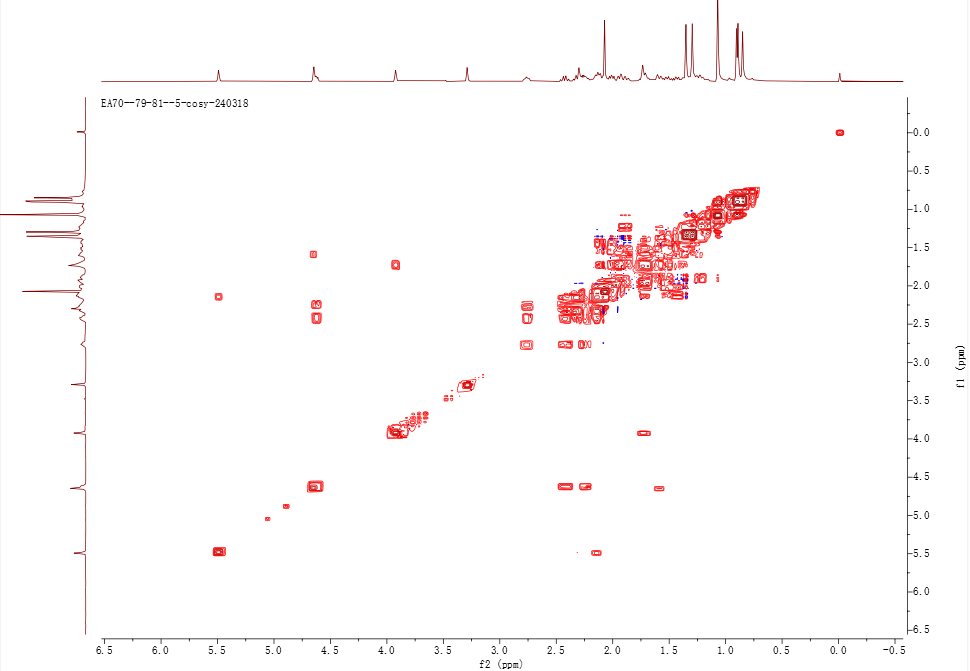


**Figure S31** ^1^H-^1^H COSY spectrum of compound **8**

**
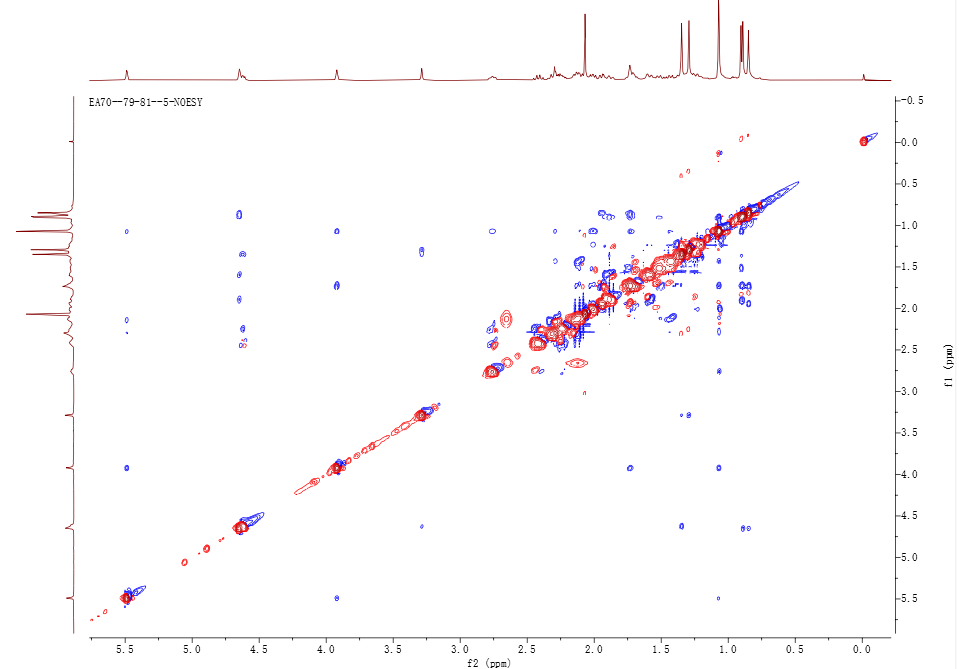
**

**Figure S32** NOESY spectrum of compound **8**


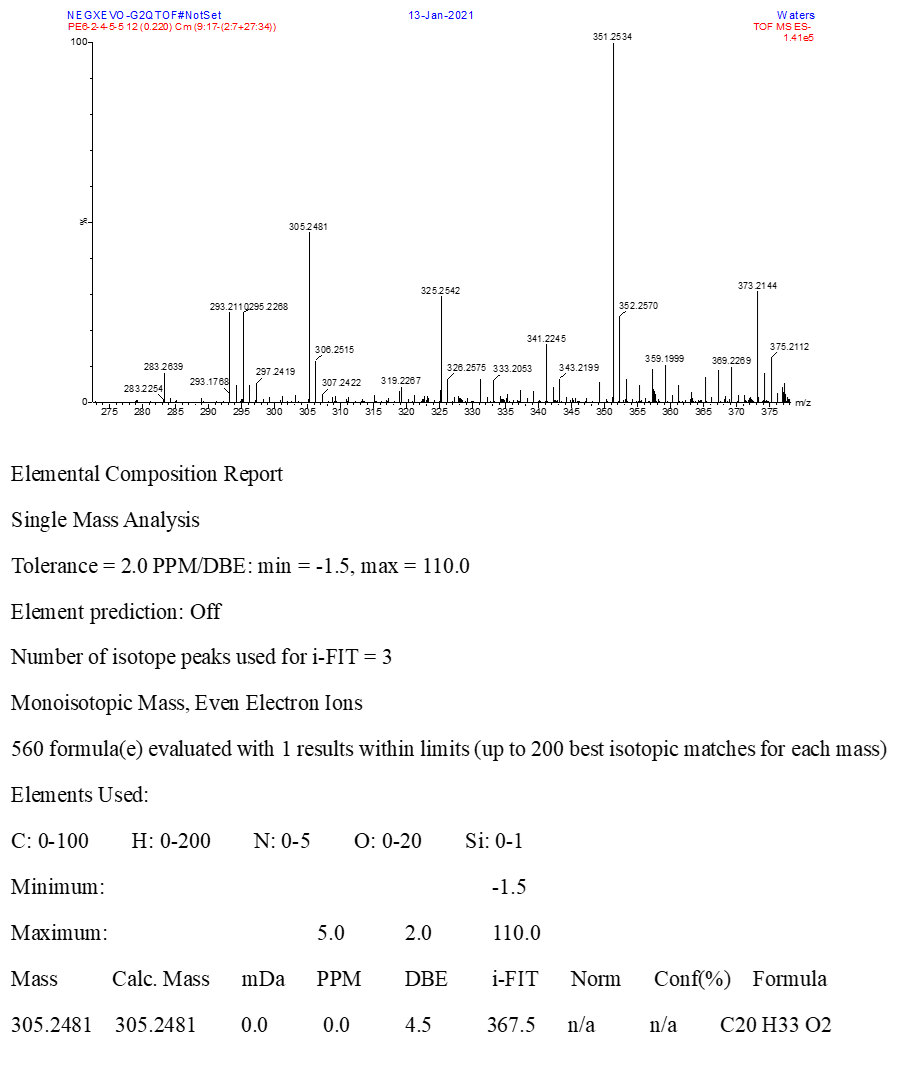


**Figure S33** HRESIMS of compound **13**


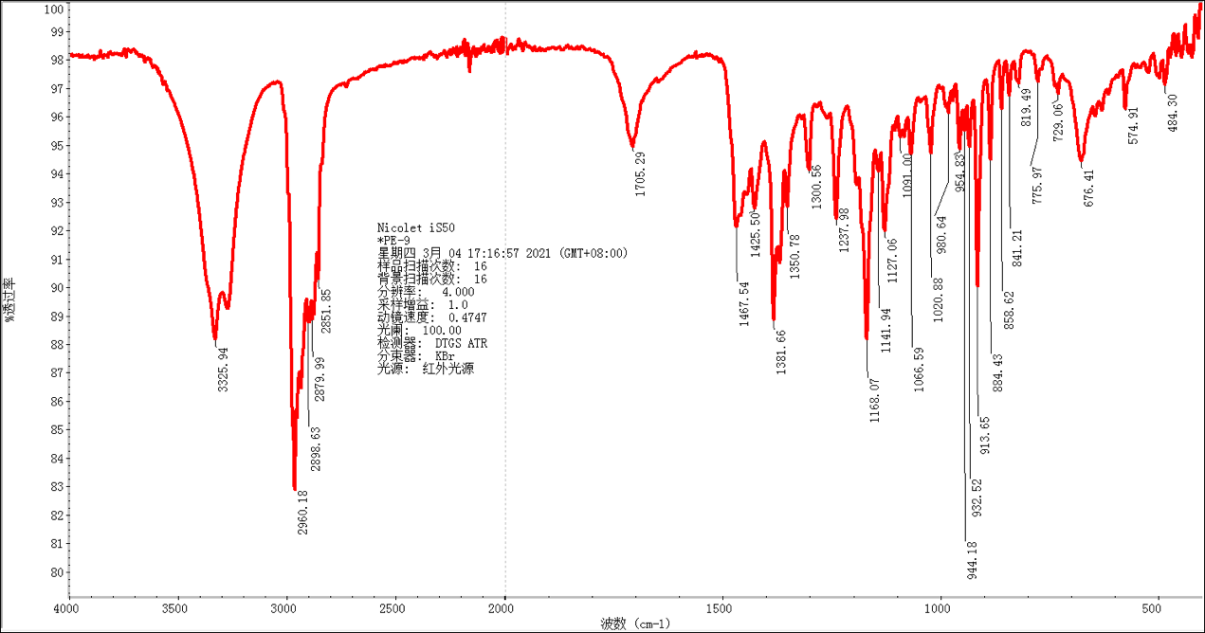


**
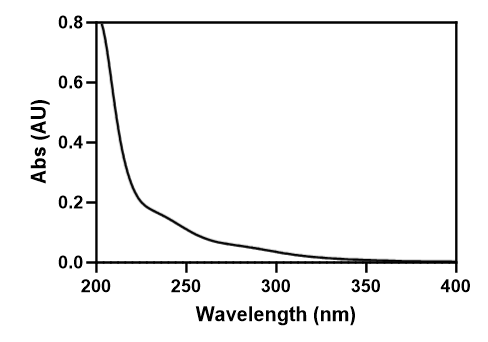
Figure S34** IR spectrum of compound **13**

**Figure S35** UV spectrum of compound **13**


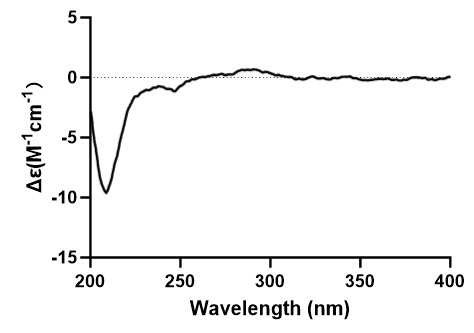


**Figure S36** ECD spectrum of compound **13**


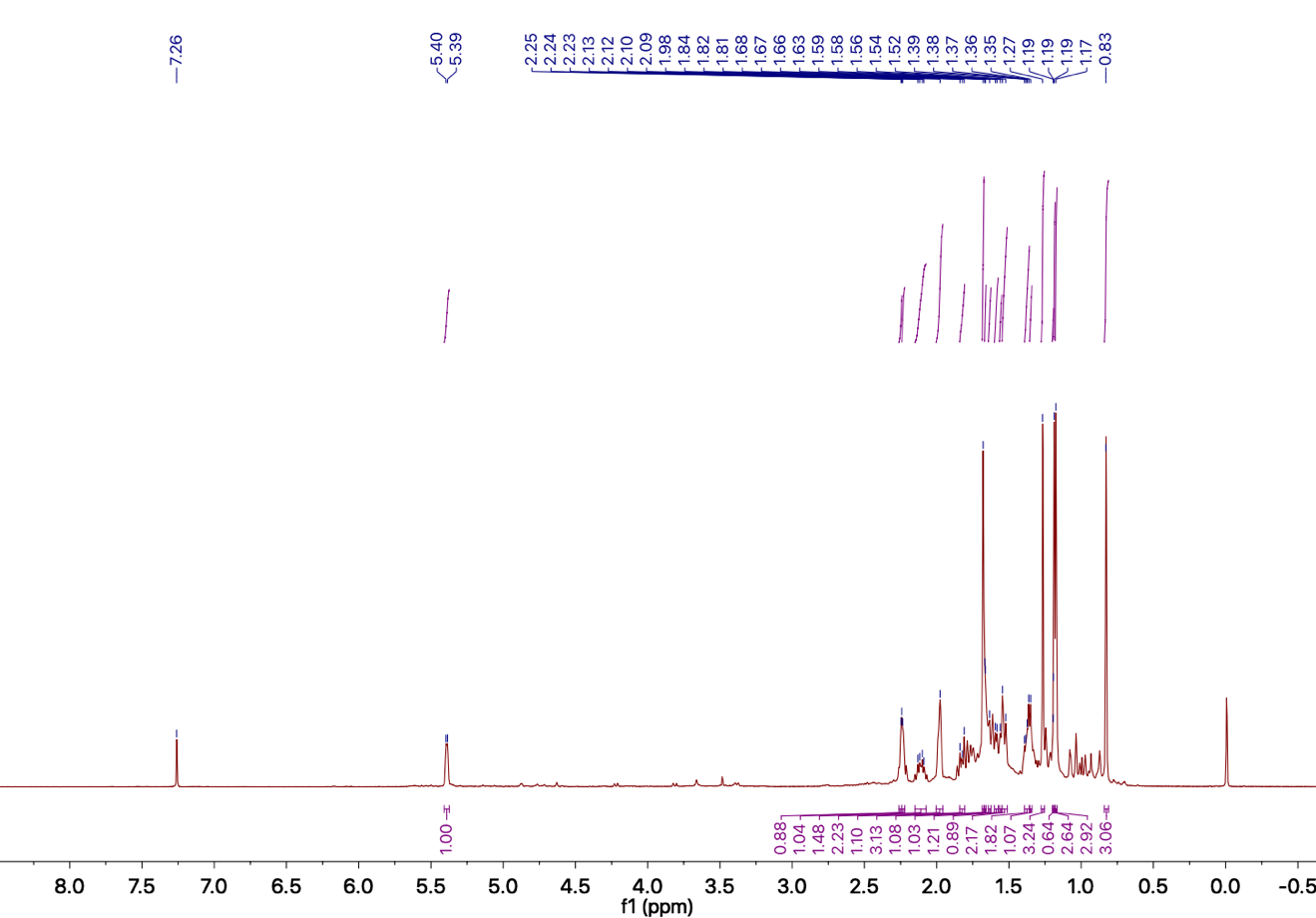


**Figure S37** ^1^H-NMR spectrum of compound **13** (500 MHz, chloroform-*d*_1_)


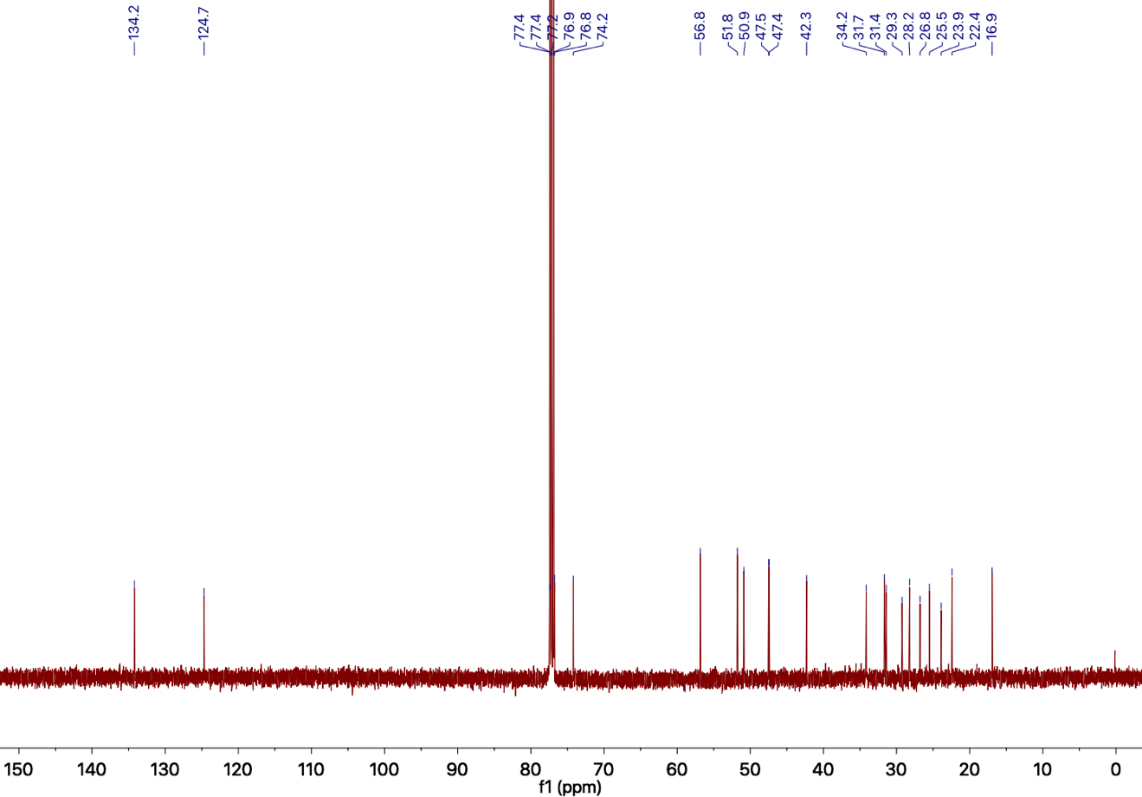


**Figure S38** ^13^C-NMR spectrum of compound **13** (125 MHz, chloroform-*d*_1_)

**
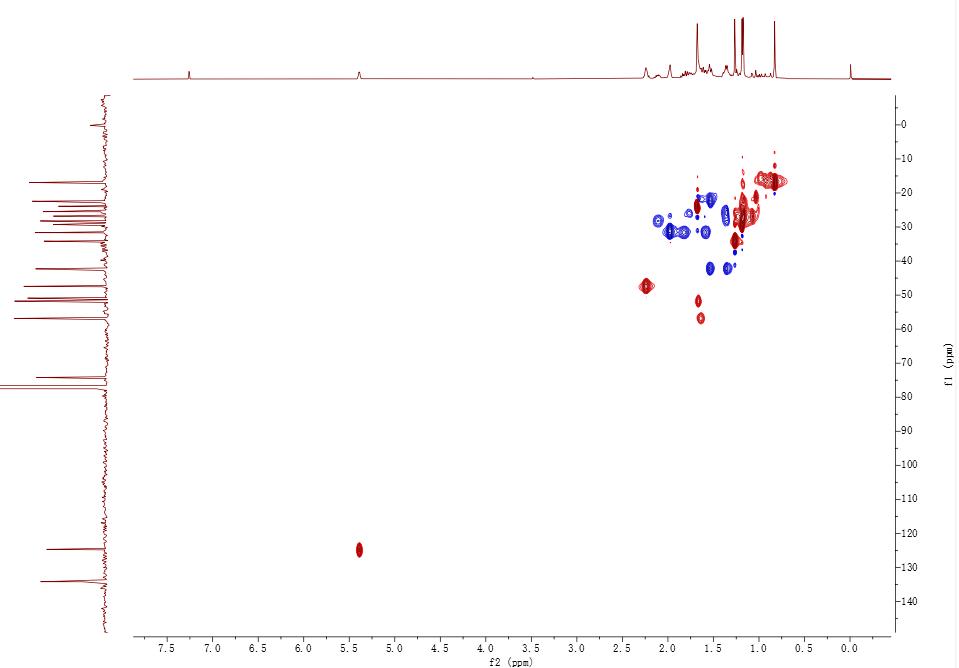
**

**Figure S39** HSQC spectrum of compound **13**


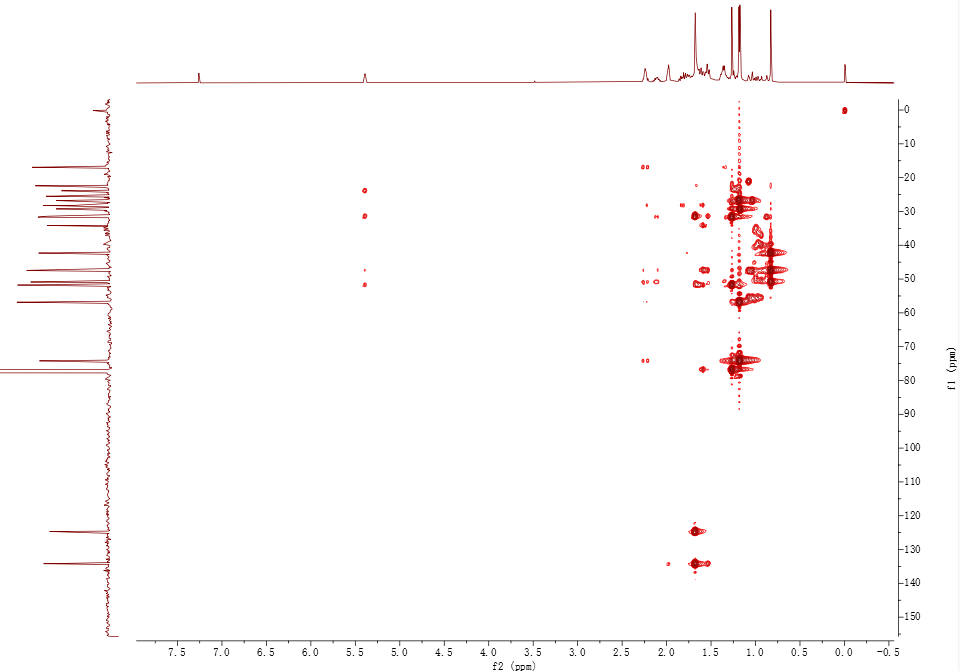


**Figure S40** HMBC spectrum of compound **13**


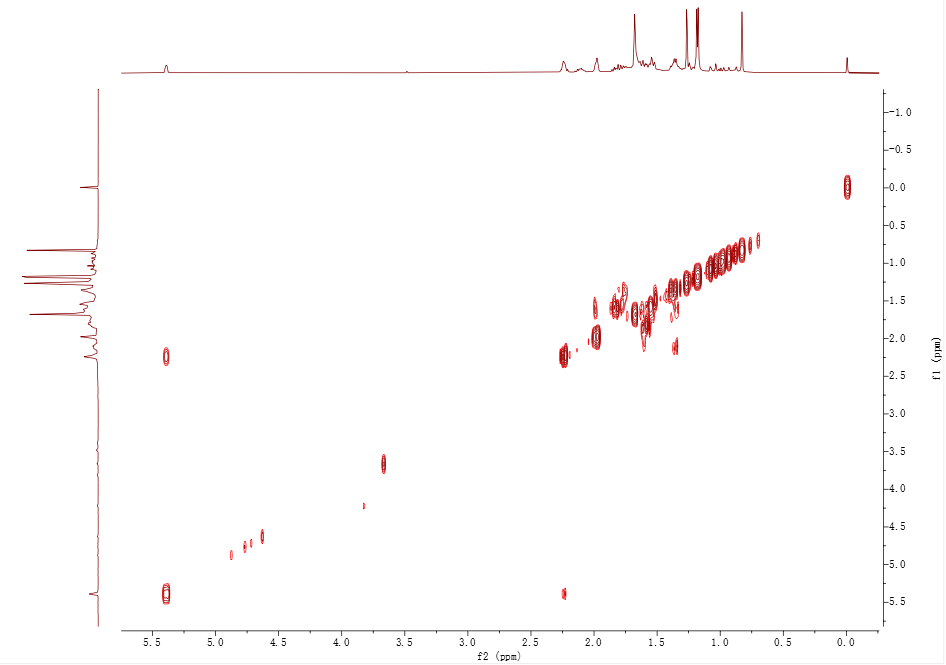


**Figure S41** ^1^H-^1^H COSY spectrum of compound **13**


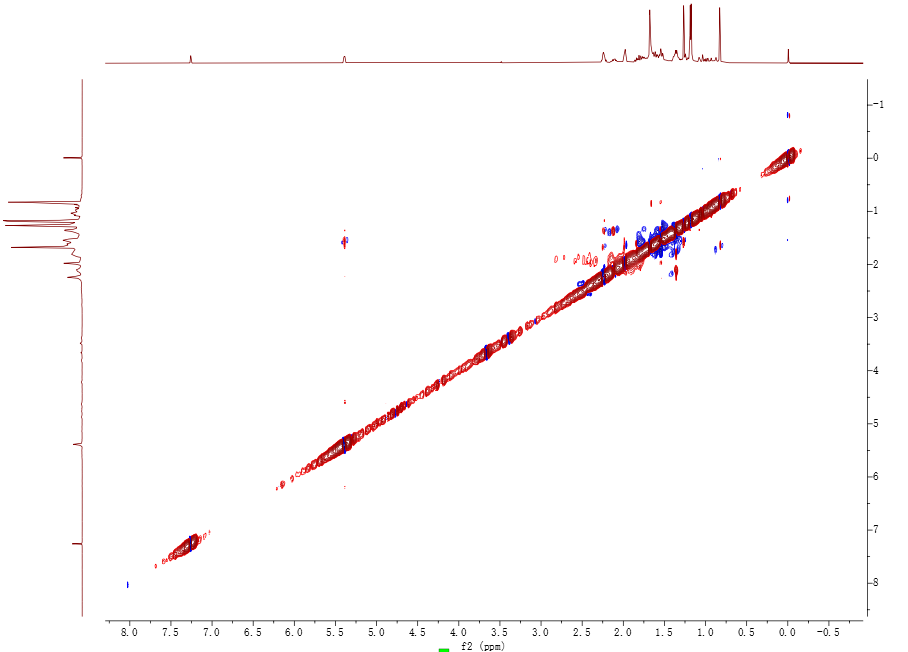


**Figure S42** NOESY spectrum of compound **13**

**Figure S43** HRESIMS of compound **14**


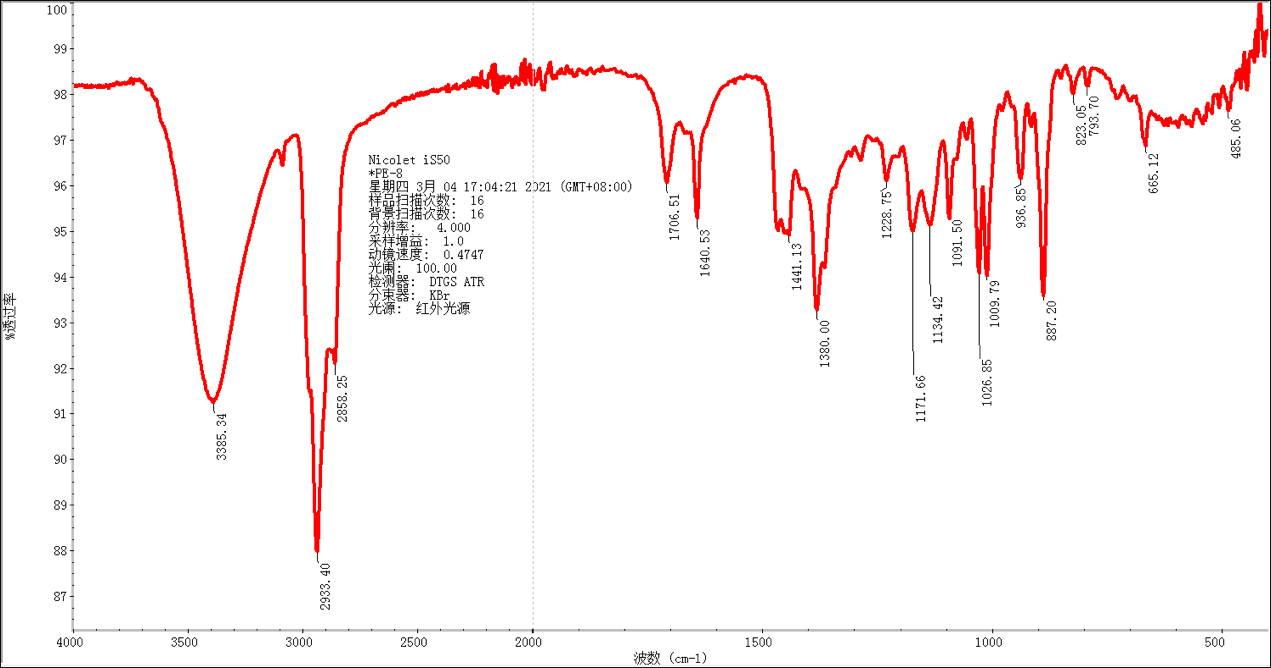


**Figure S44** IR spectrum of compound **14**

**
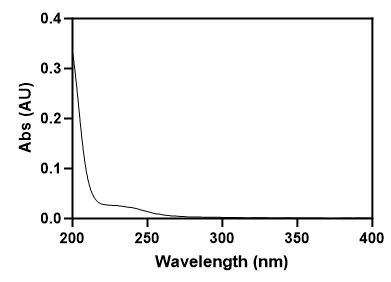
**

**Figure S45** UV spectrum of compound **14**


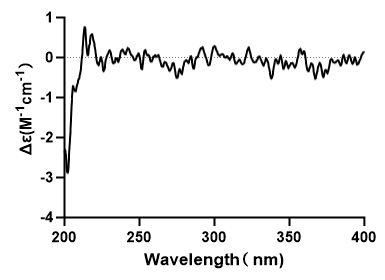


**Figure S46** ECD spectrum of compound **14**


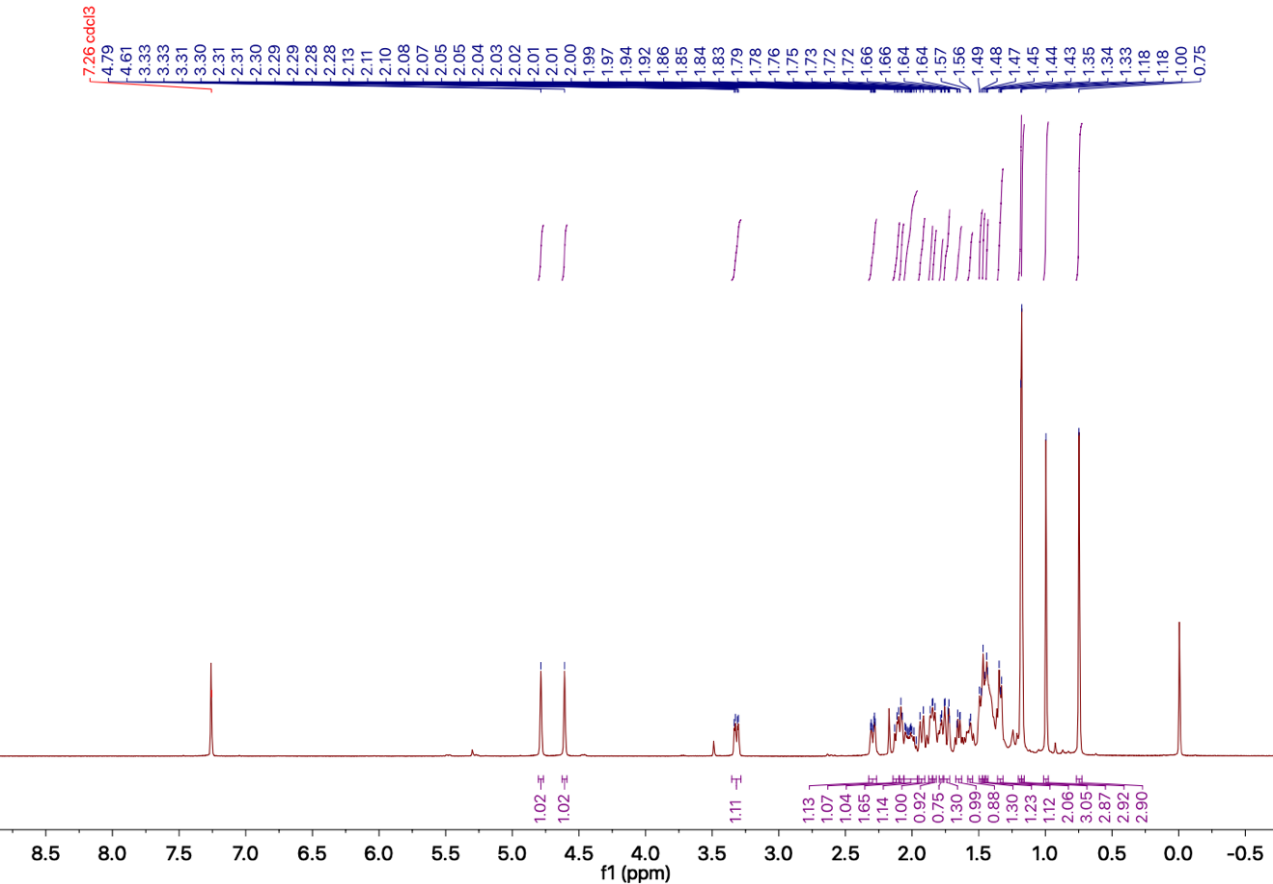


**Figure S47** ^1^H-NMR spectrum of compound **14** (500 MHz, chloroform-*d*_1_)


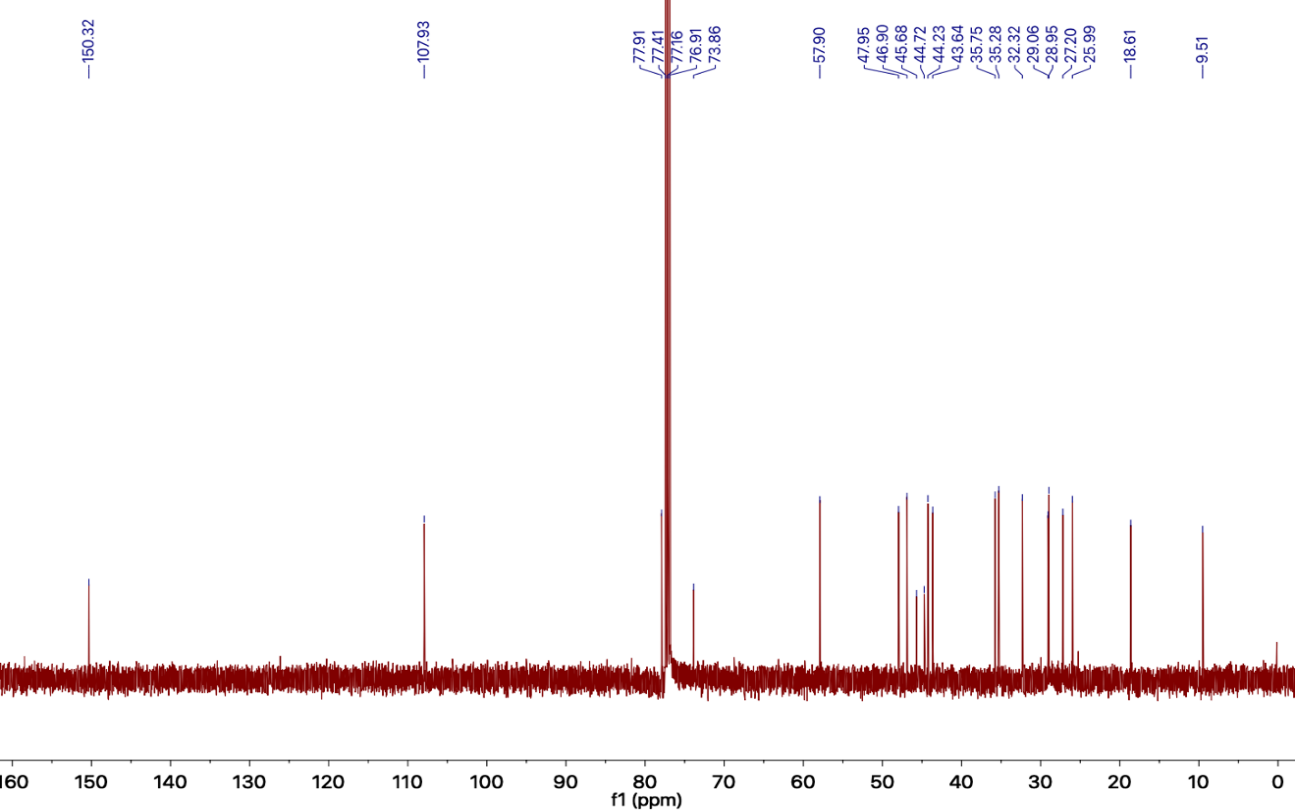


**Figure S48** ^13^C-NMR spectrum of compound **14** (125 MHz, chloroform-*d*_1_)

**
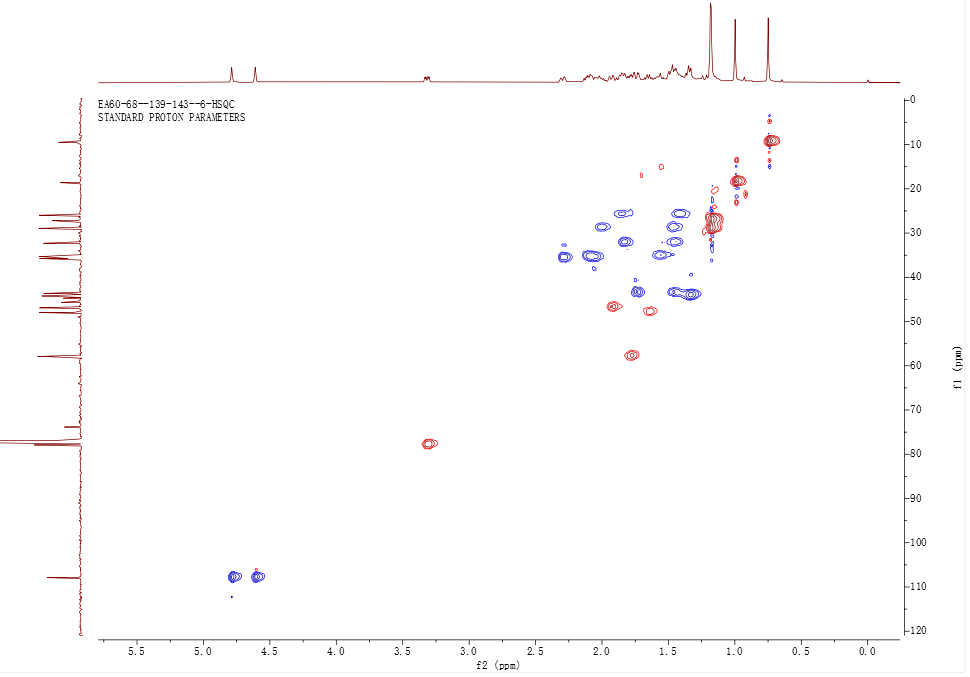
**

**Figure S49** HSQC spectrum of compound **14**

**
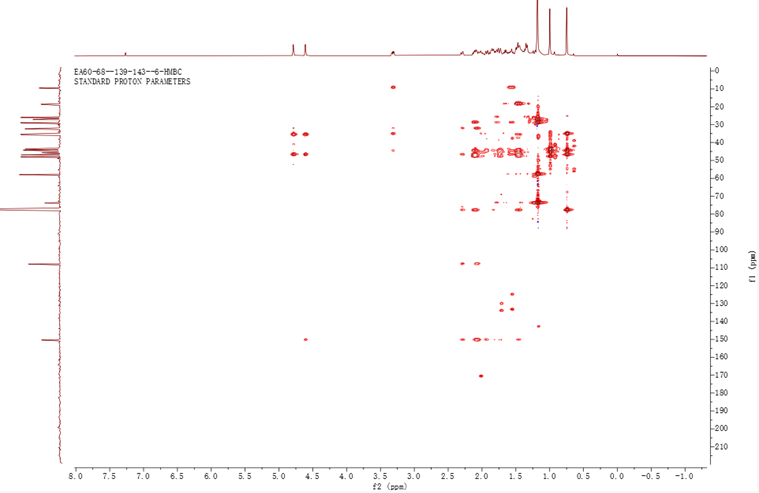
**

**Figure S50** HSQC spectrum of compound **14**


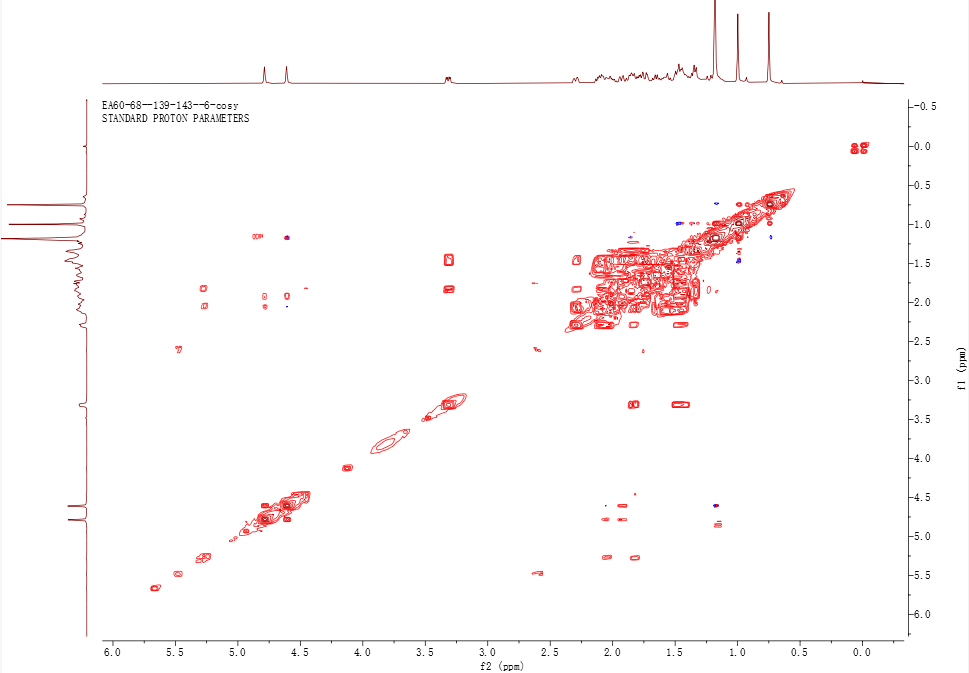


**Figure S51** ^1^H-^1^H COSY spectrum of compound **14**

**
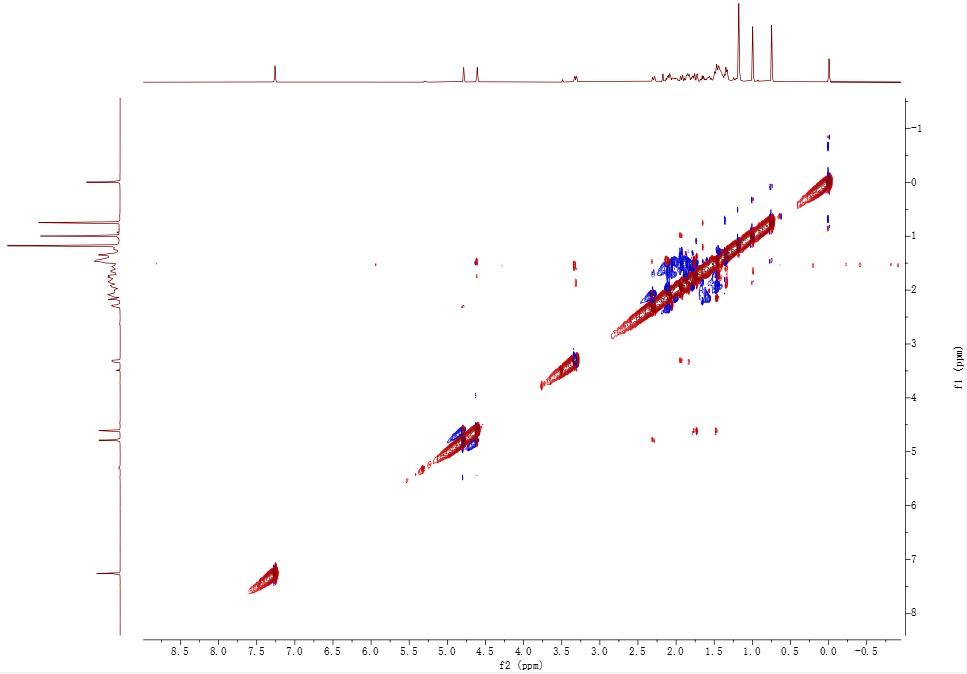
**

**Figure S52** NOESY spectrum of compound **14**

**Figure S53** HRESIMS of compound **15**


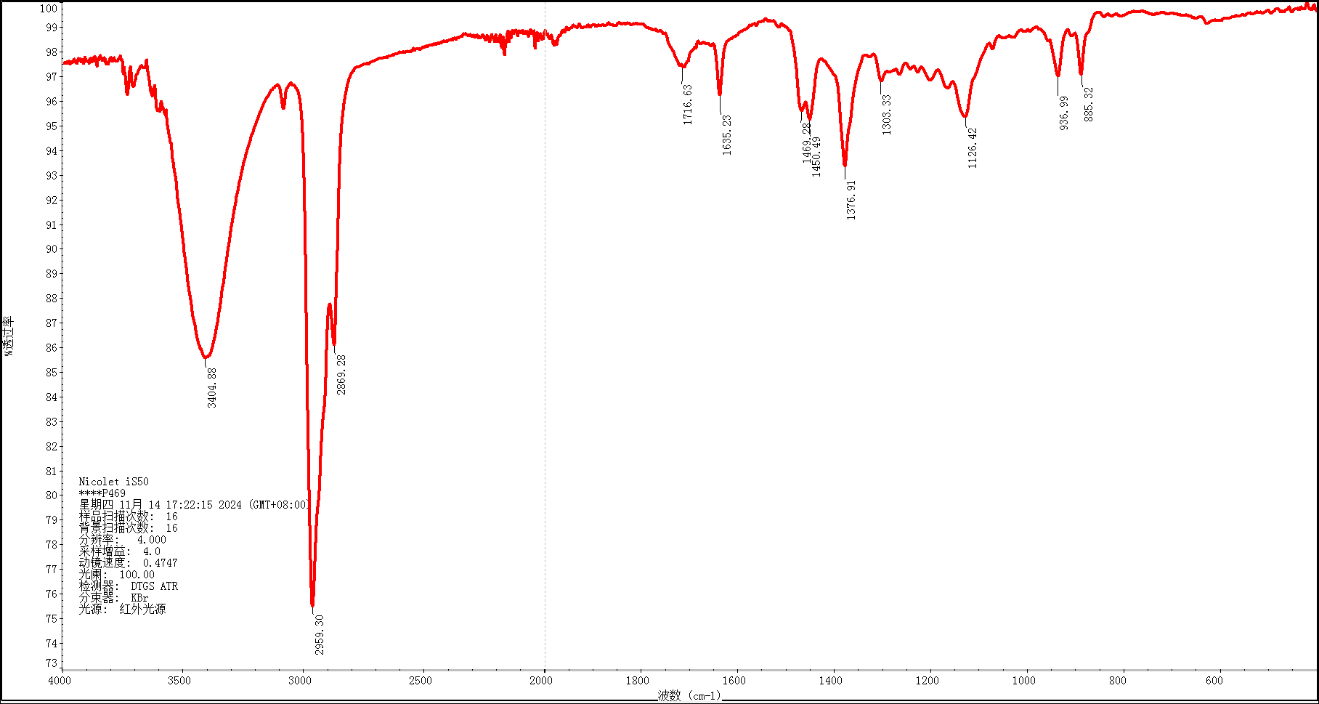


**Figure S54** IR spectrum of compound **15**

**Figure S55** UV spectrum of compound **15**


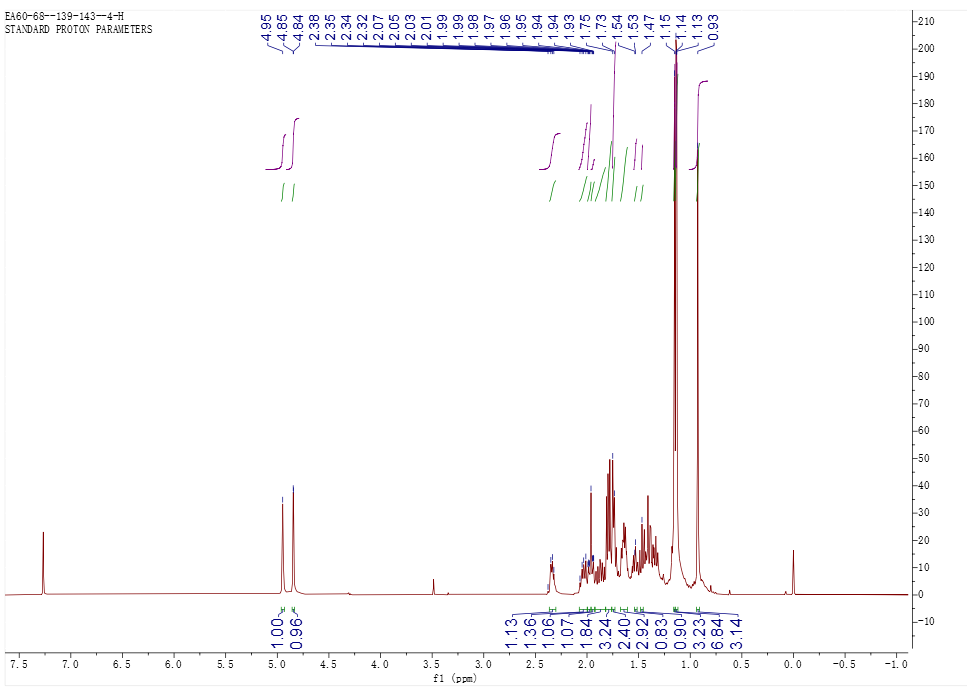


**Figure S56** ^1^H-NMR spectrum of compound **15** (500 MHz, chloroform-*d*_1_)


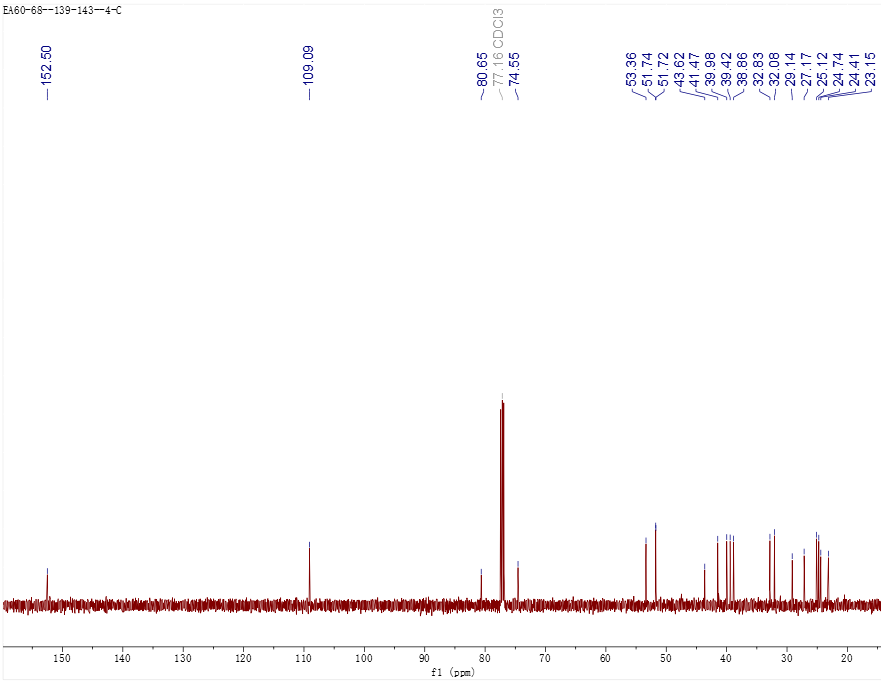


**Figure S57** ^13^C-NMR spectrum of compound **15** (125 MHz, chloroform-*d*_1_)

**
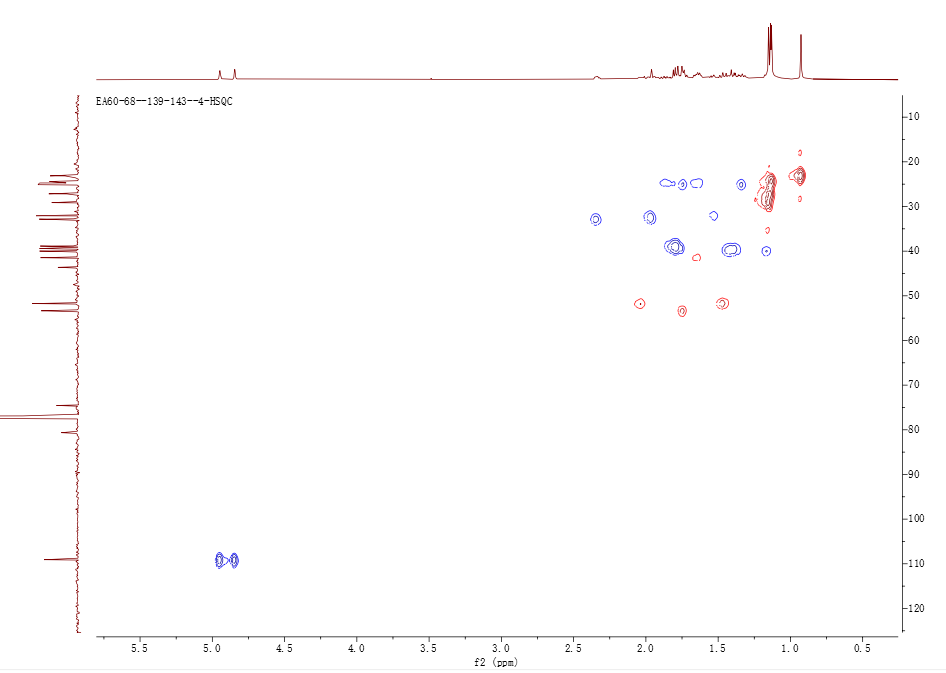
**

**Figure S58** HSQC spectrum of compound **15**

**
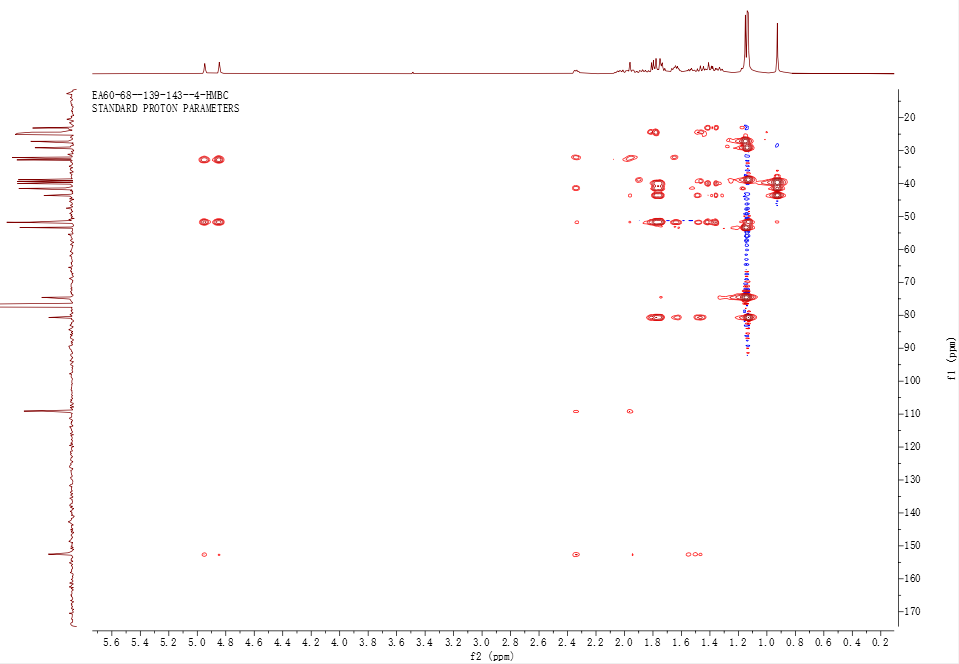
**

**Figure S59** HMBC spectrum of compound **15**


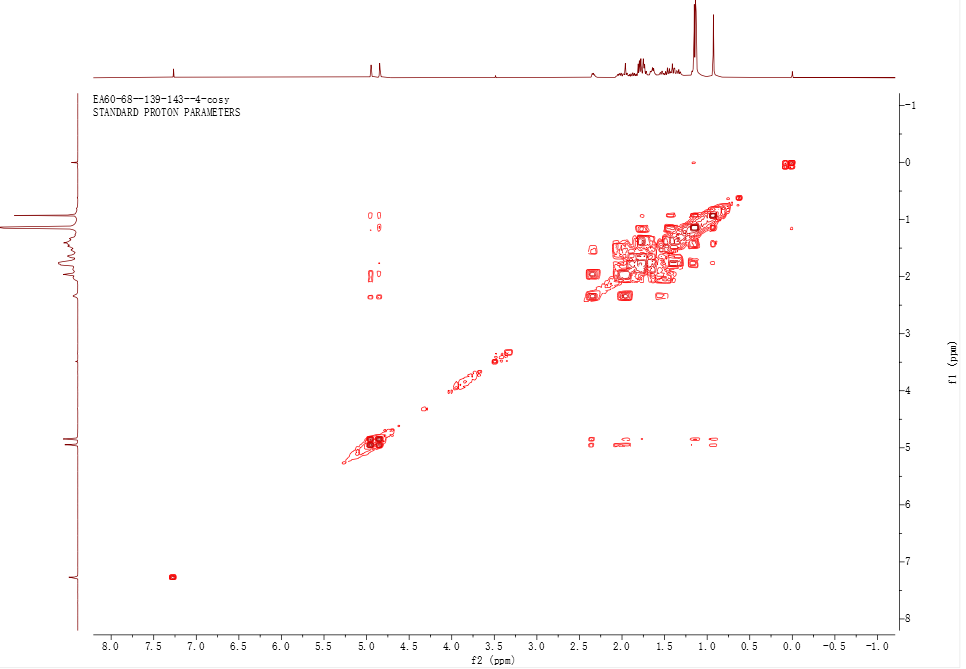


**Figure S60** ^1^H-^1^H COSY spectrum of compound **15**

**
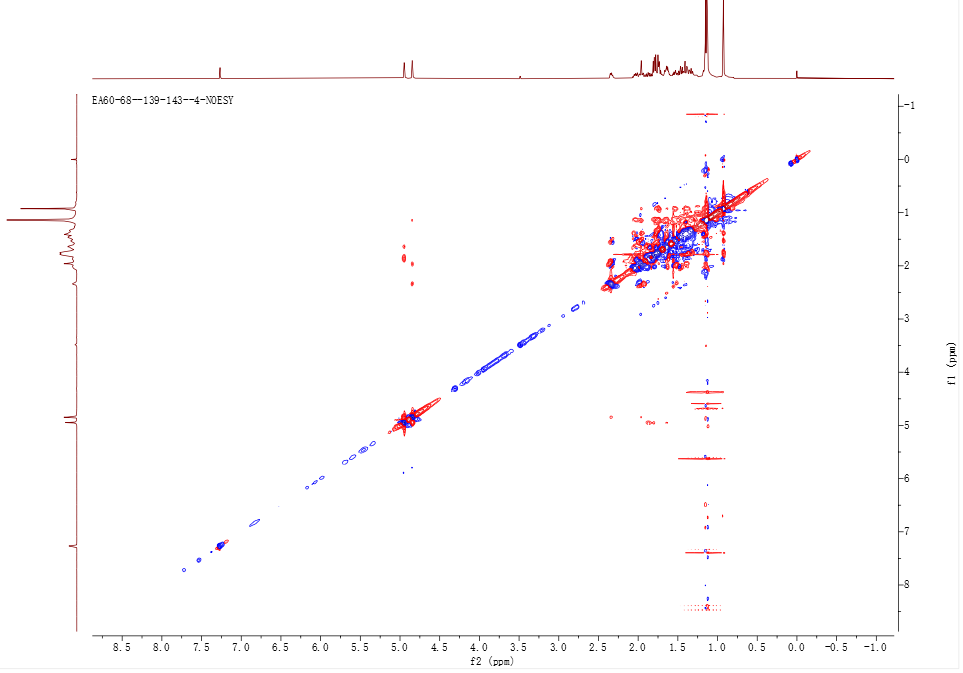
**

**Figure S61** NOESY spectrum of compound **15**

**Figure S62** HRESIMS of compound **16**


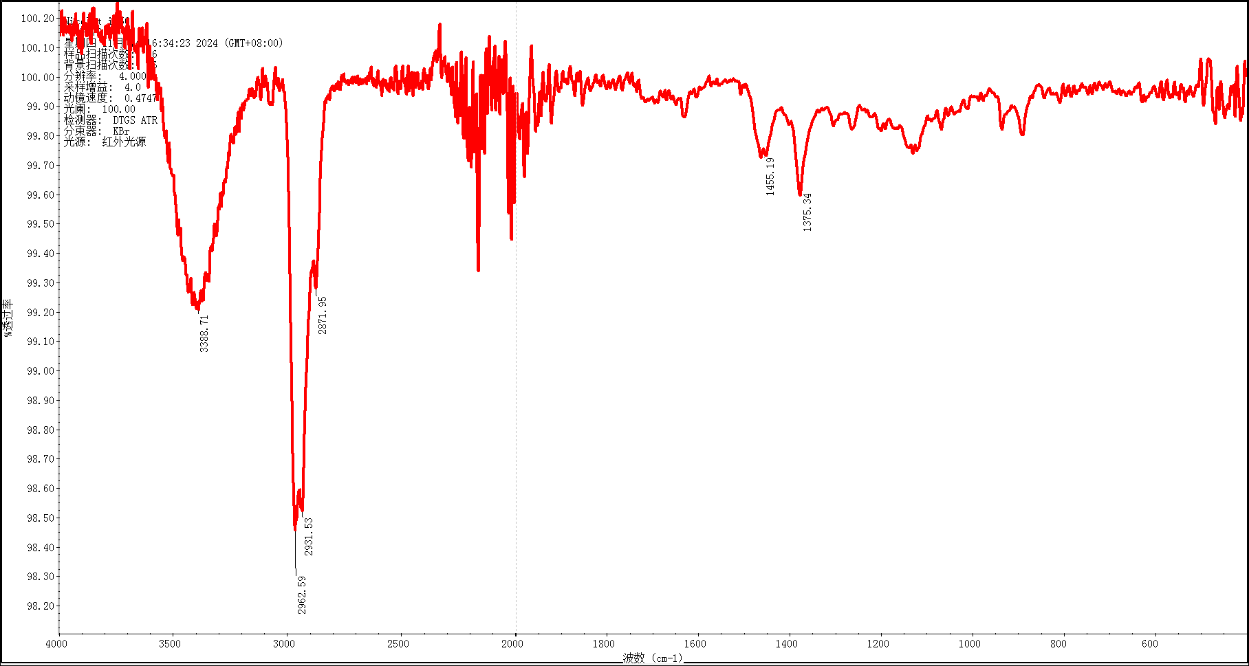


**Figure S63** IR spectrum of compound **16**

**
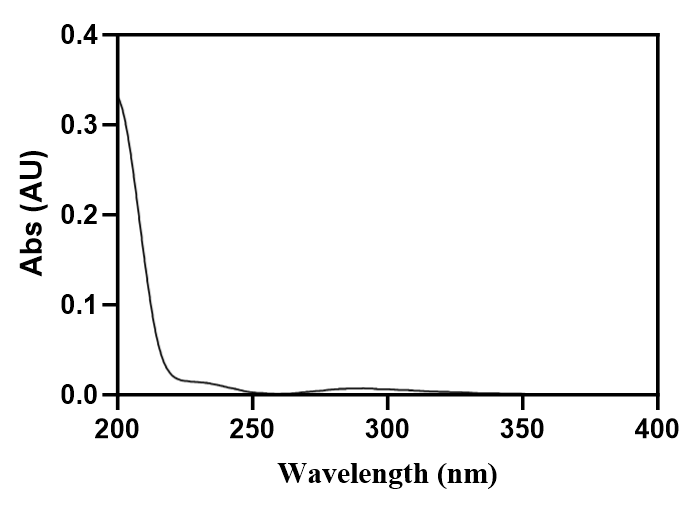
Figure S64** UV spectrum of compound **16**


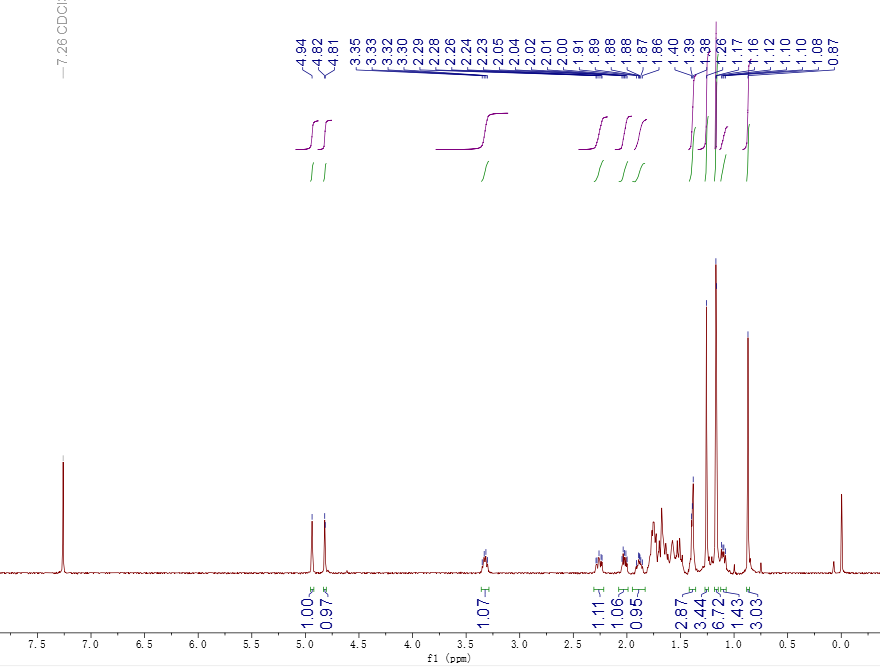


**Figure S65** ^1^H-NMR spectrum of compound **16** (500 MHz, chloroform-*d*_1_)


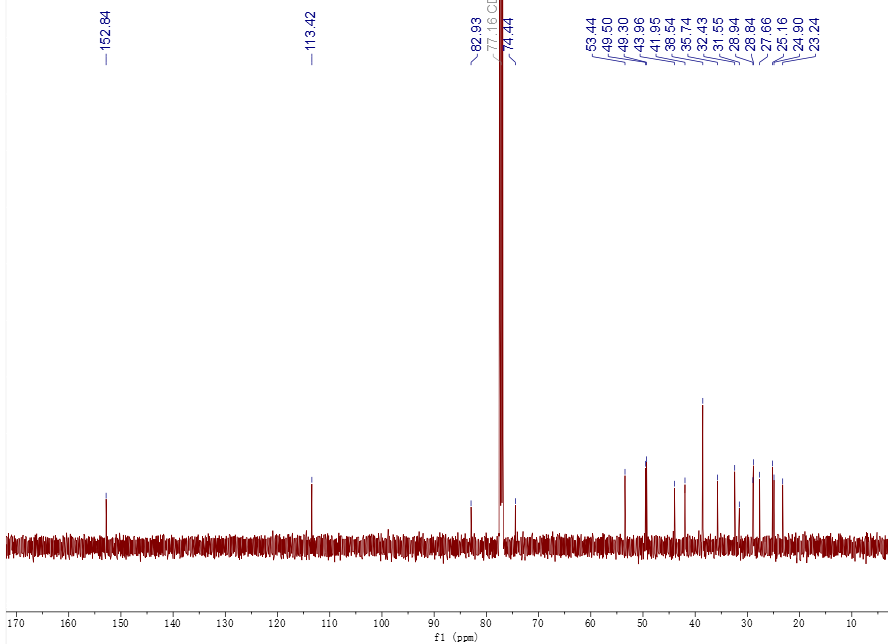


**Figure S66** ^13^C-NMR spectrum of compound **16** (125 MHz, chloroform-*d*_1_)

**
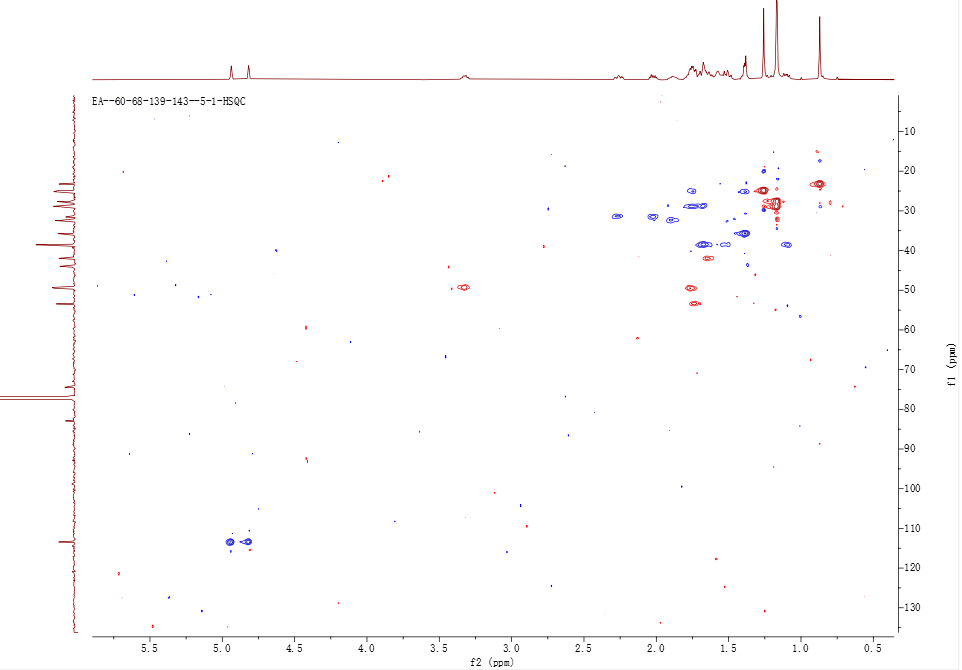
**

**Figure S67** HSQC spectrum of compound **16**

**
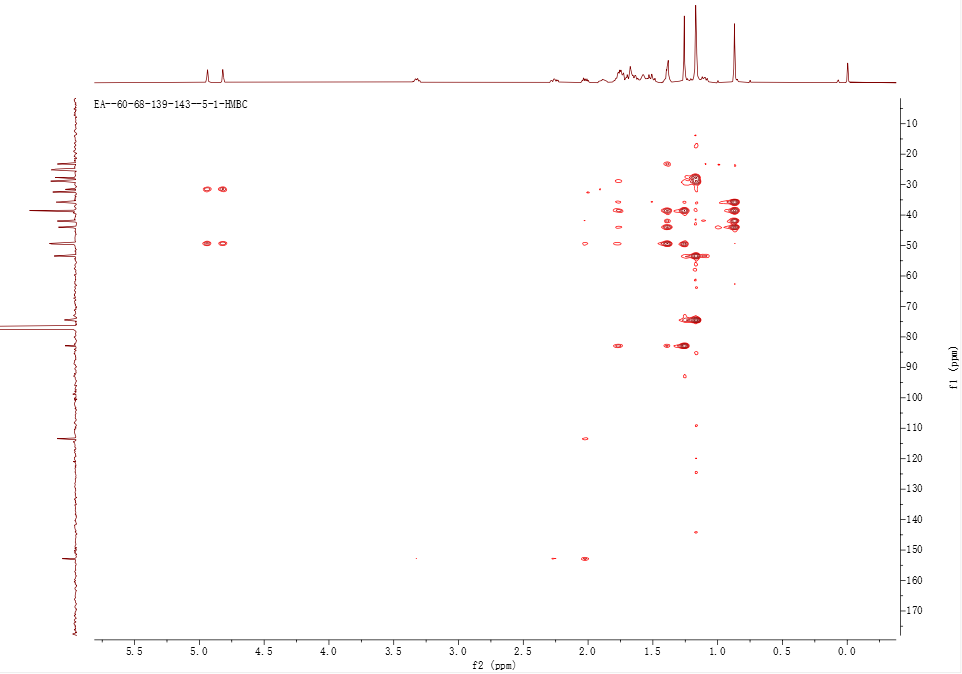
**

**Figure S68** HMBC spectrum of compound **16**


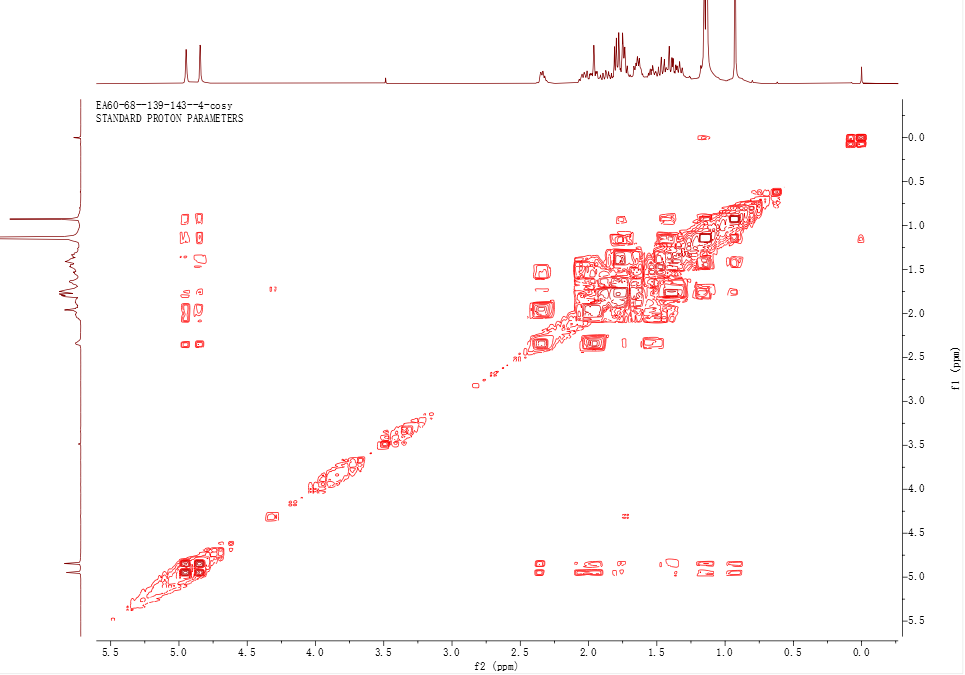


**Figure S69** ^1^H-^1^H COSY spectrum of compound **16**

**
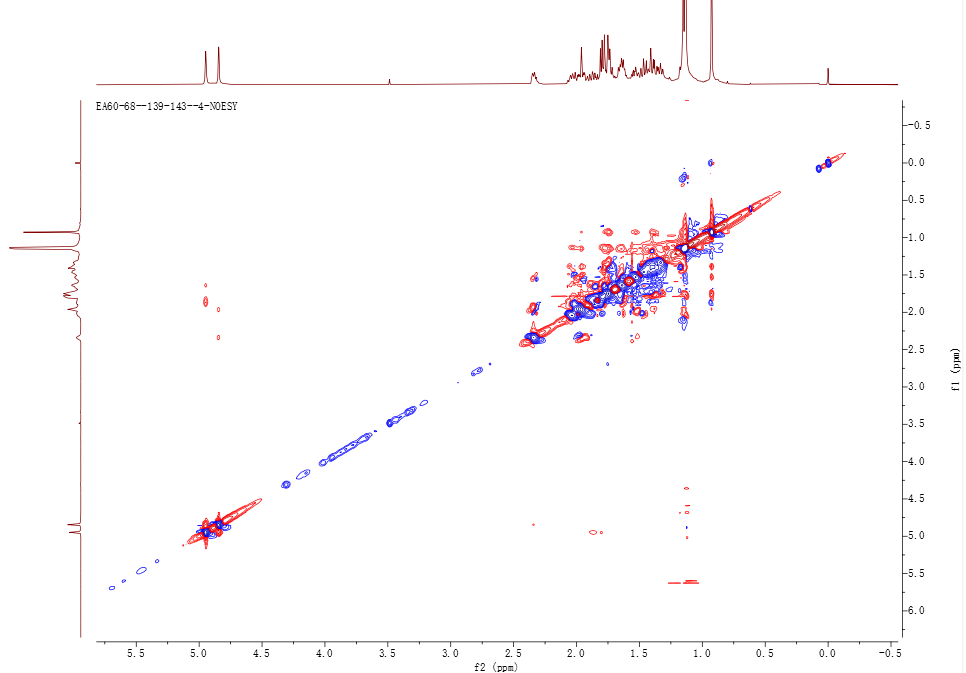
**

**Figure S70** NOESY spectrum of compound **16**


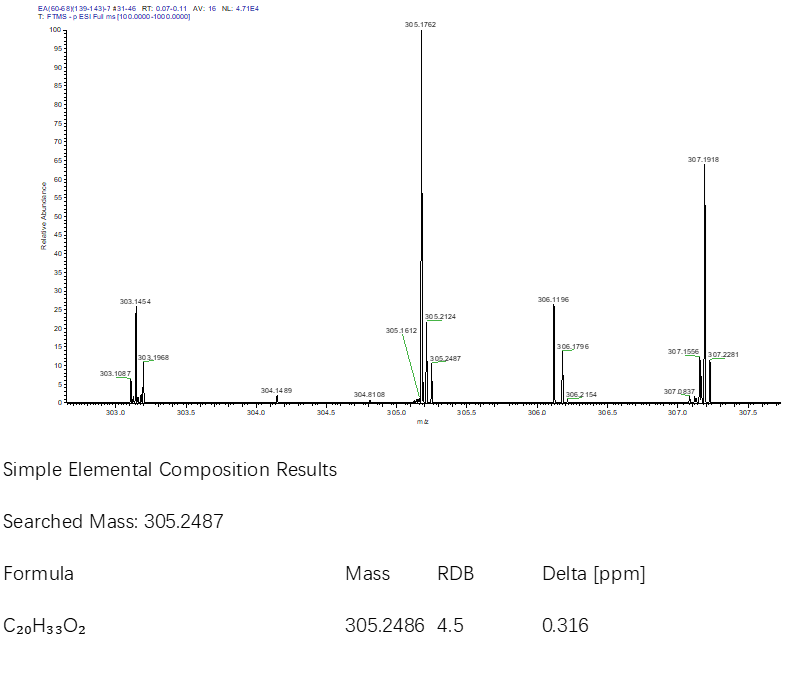


**Figure S71** HRESIMS of compound **19**


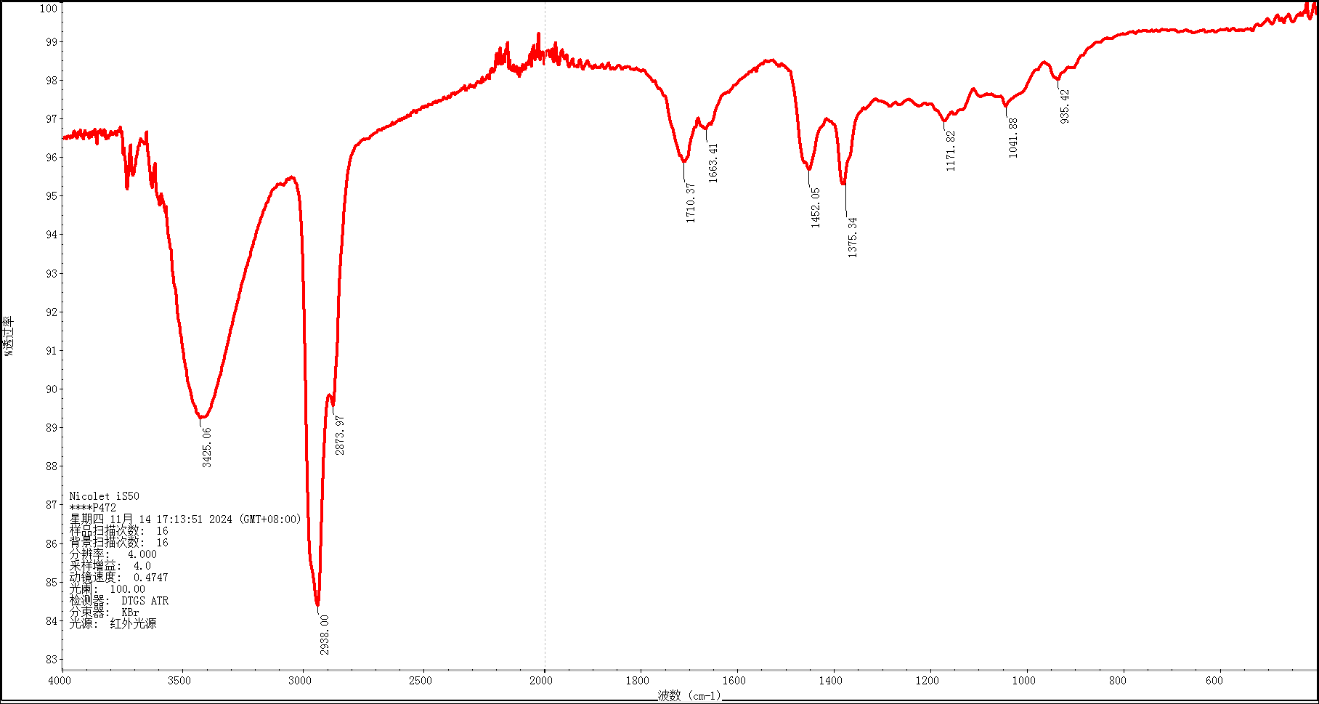


**Figure S72** IR spectrum of compound **19**

**
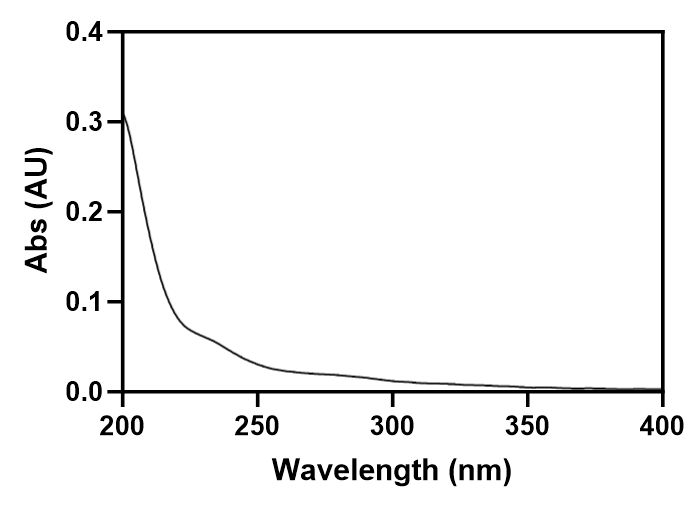
Figure S73** UV spectrum of compound **19**


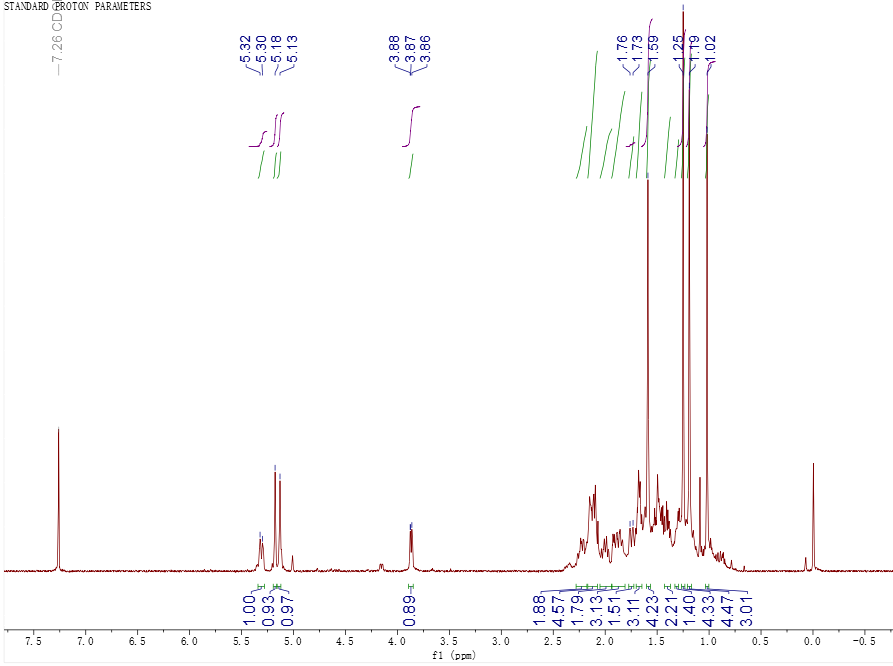


**Figure S74** ^1^H-NMR spectrum of compound **19** (500 MHz, chloroform-*d*_1_)


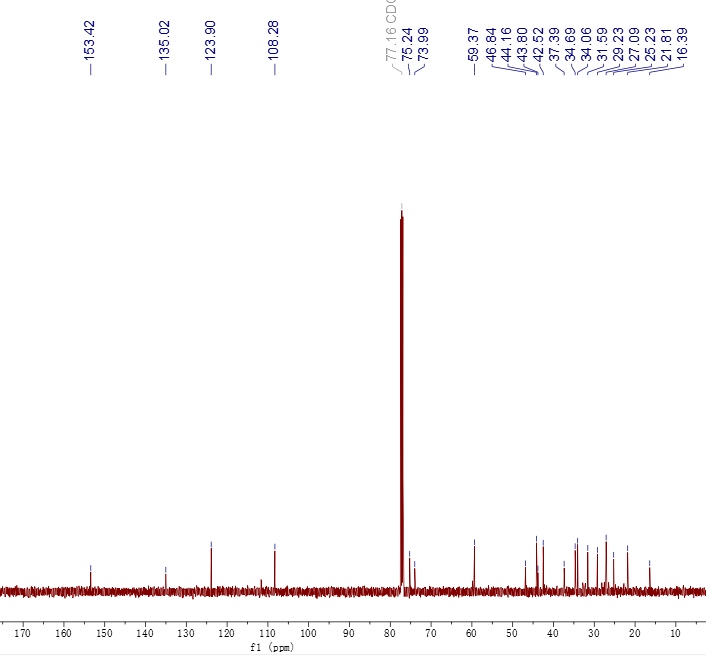


**Figure S75** ^13^C-NMR spectrum of compound **19** (125 MHz, chloroform-*d*_1_)

**
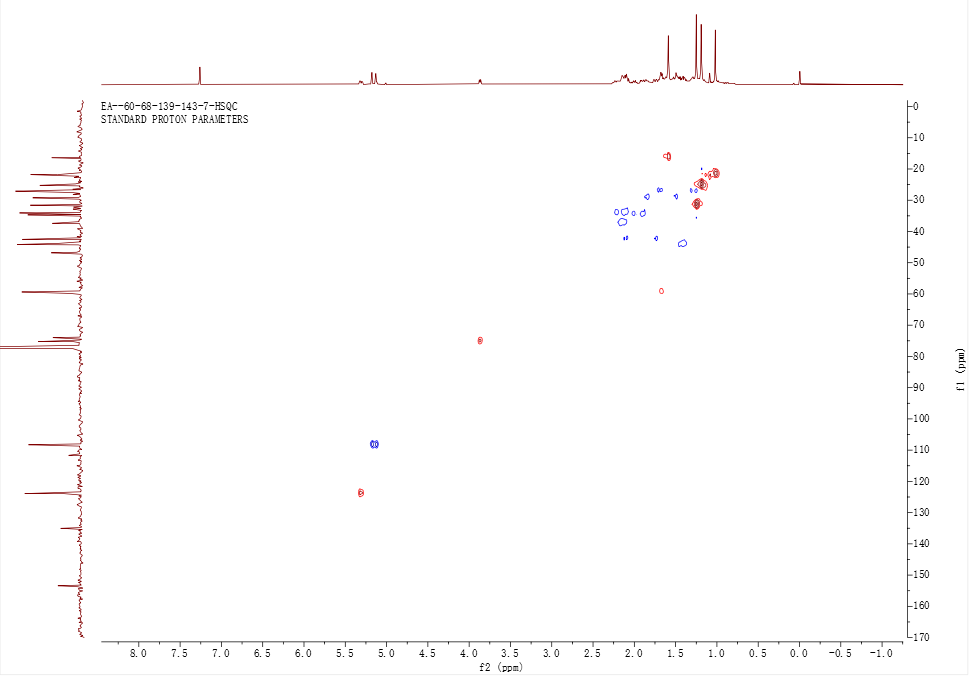
**

**Figure S76** HSQC spectrum of compound **19**

**
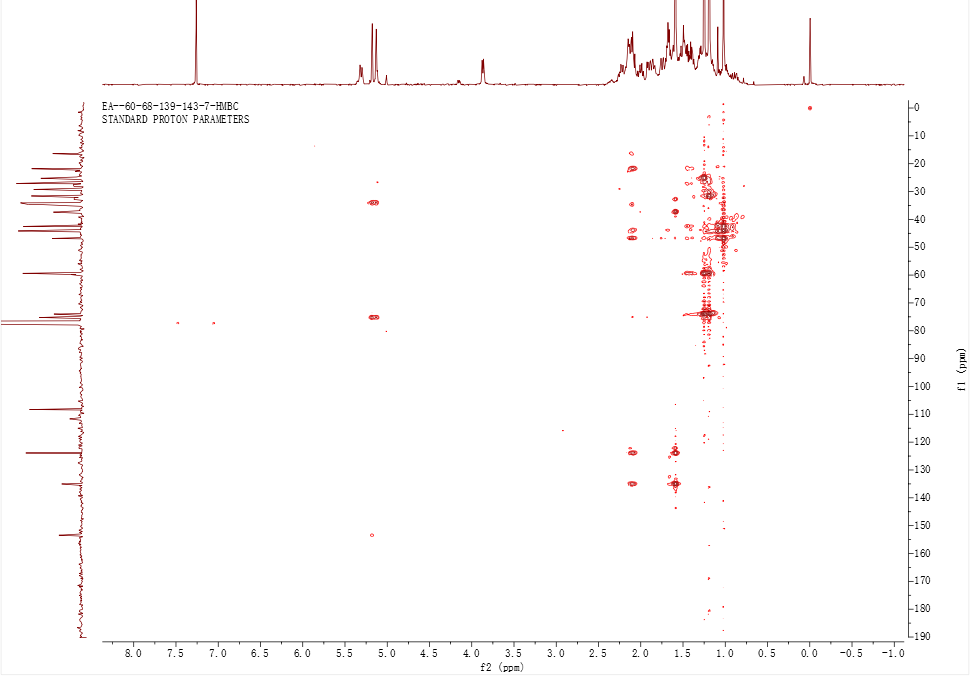
**

**Figure S77** HMBC spectrum of compound **19**


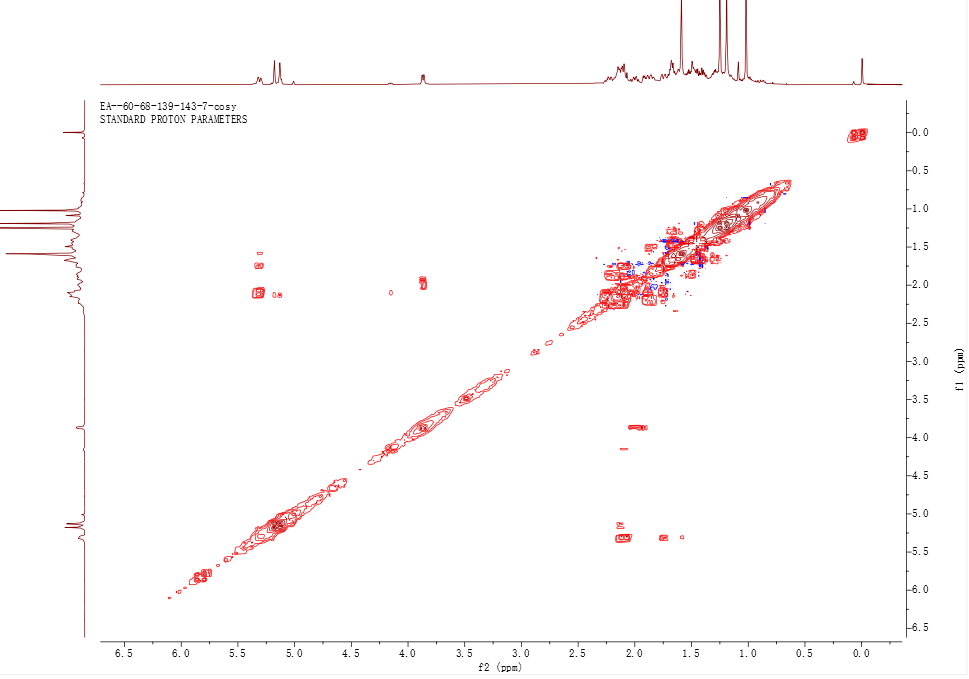


**Figure S78** ^1^H-^1^H COSY spectrum of compound **19**

**
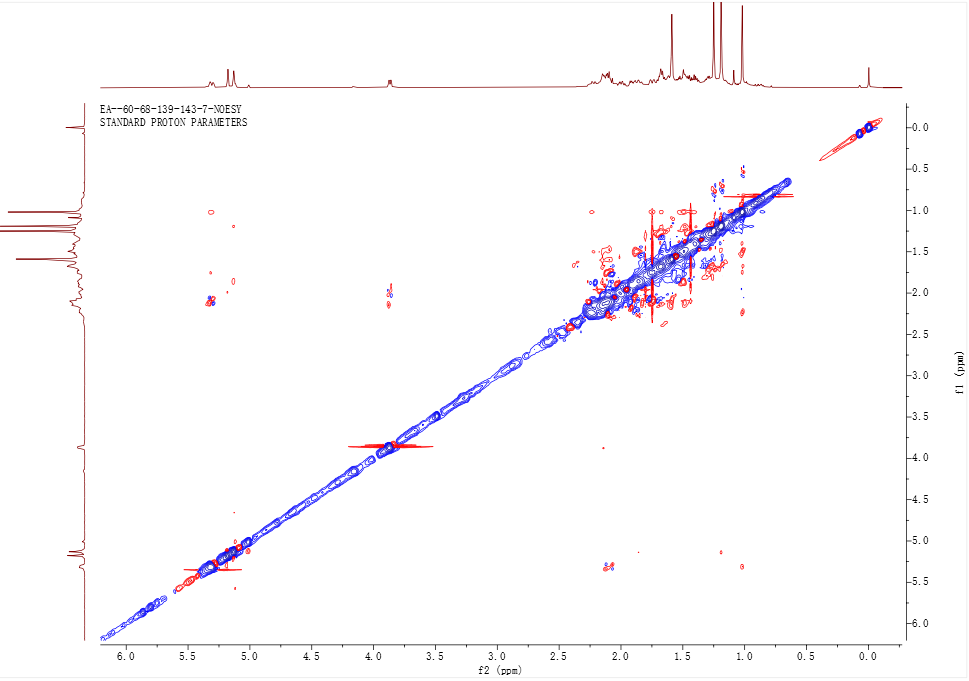
**

**Figure S79** NOESY spectrum of compound **19**

**Figure S80** HRESIMS of compound **20**


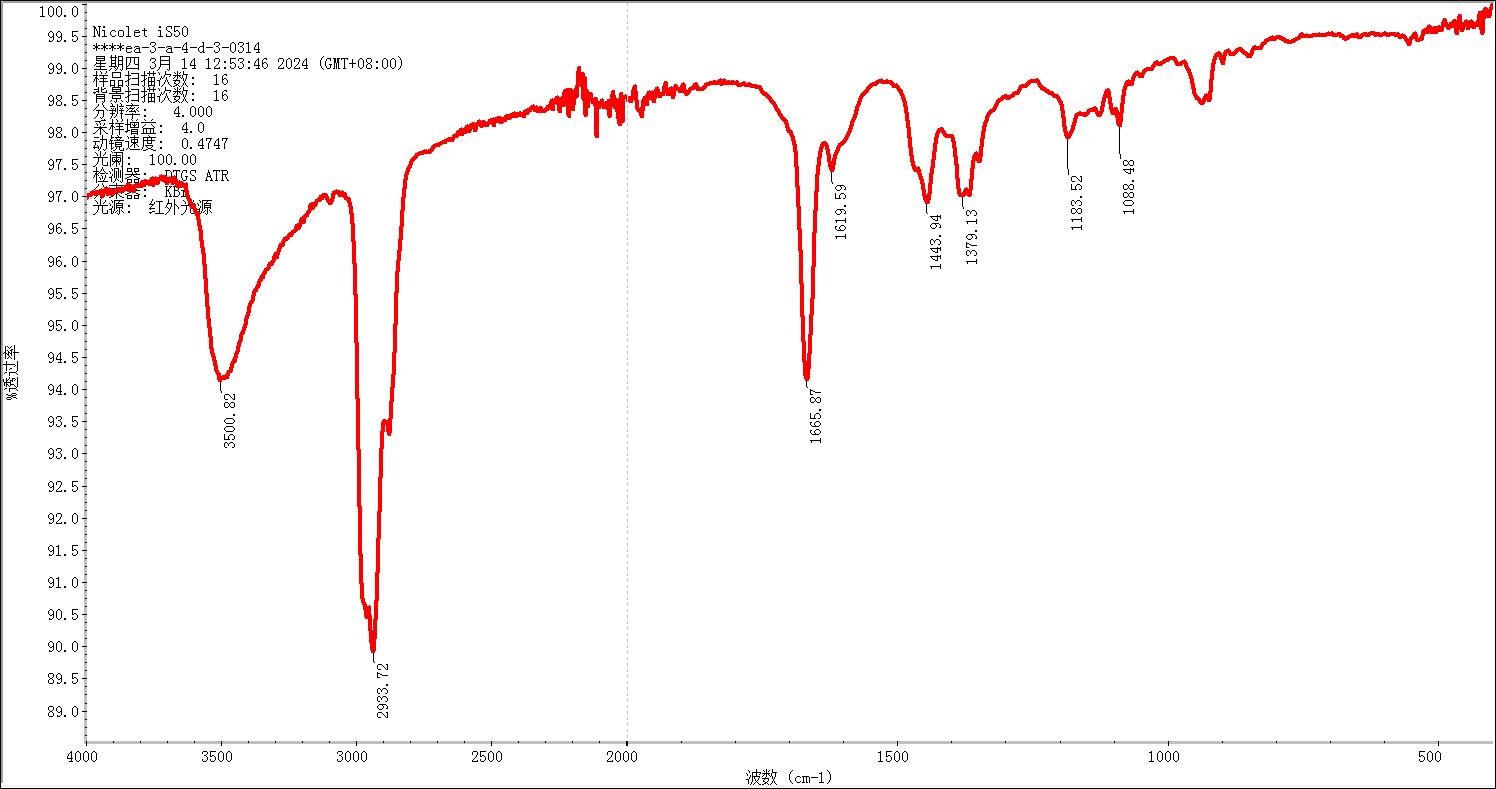


**Figure S81** IR spectrum of compound **20**


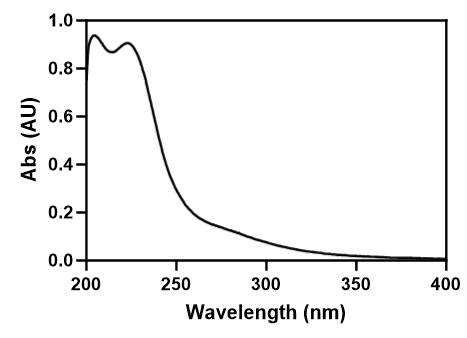
**Figure S82** UV spectrum of compound **20**


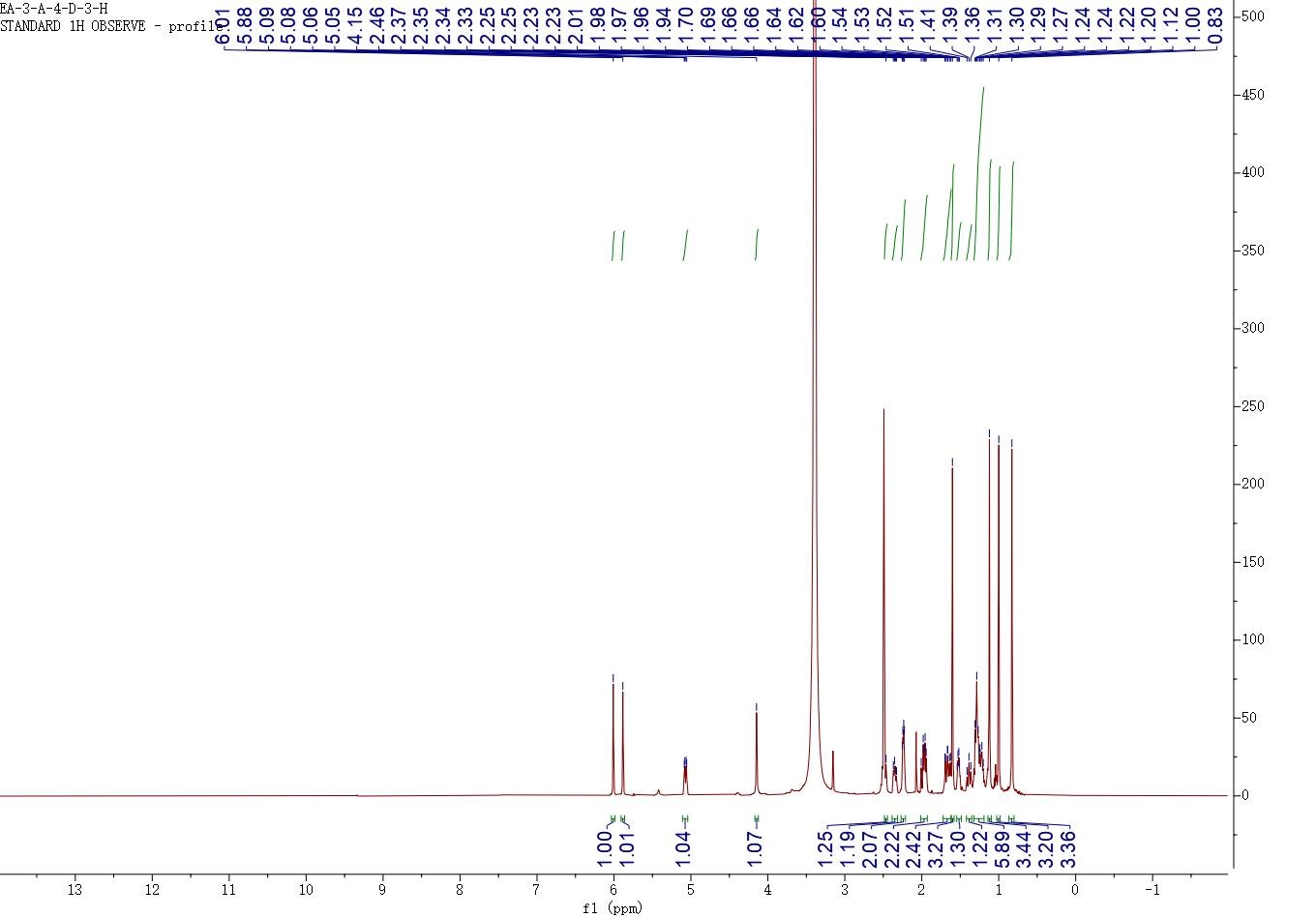


**Figure S83** ^1^H-NMR spectrum of compound **20** (500 MHz, chloroform-*d*_1_)


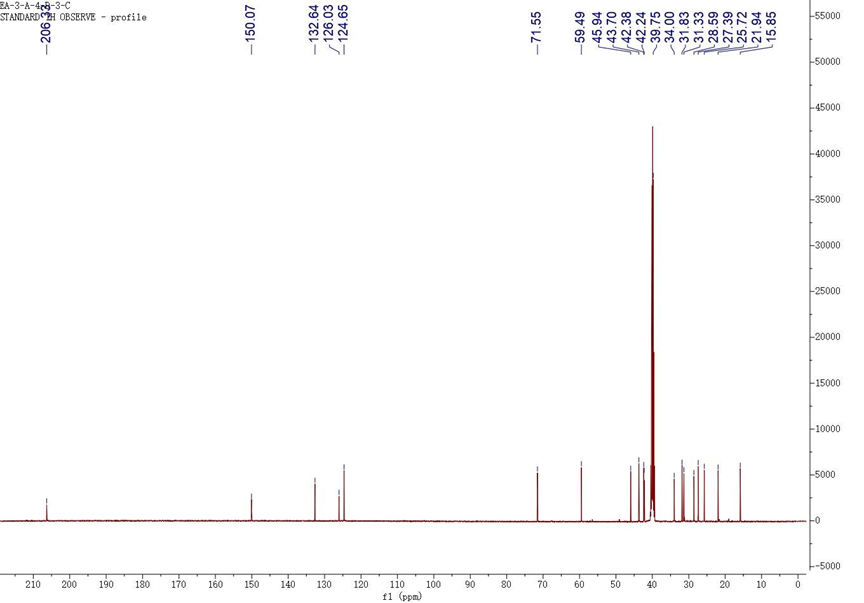


**Figure S84** ^13^C-NMR spectrum of compound **20** (125 MHz, chloroform-*d*_1_)


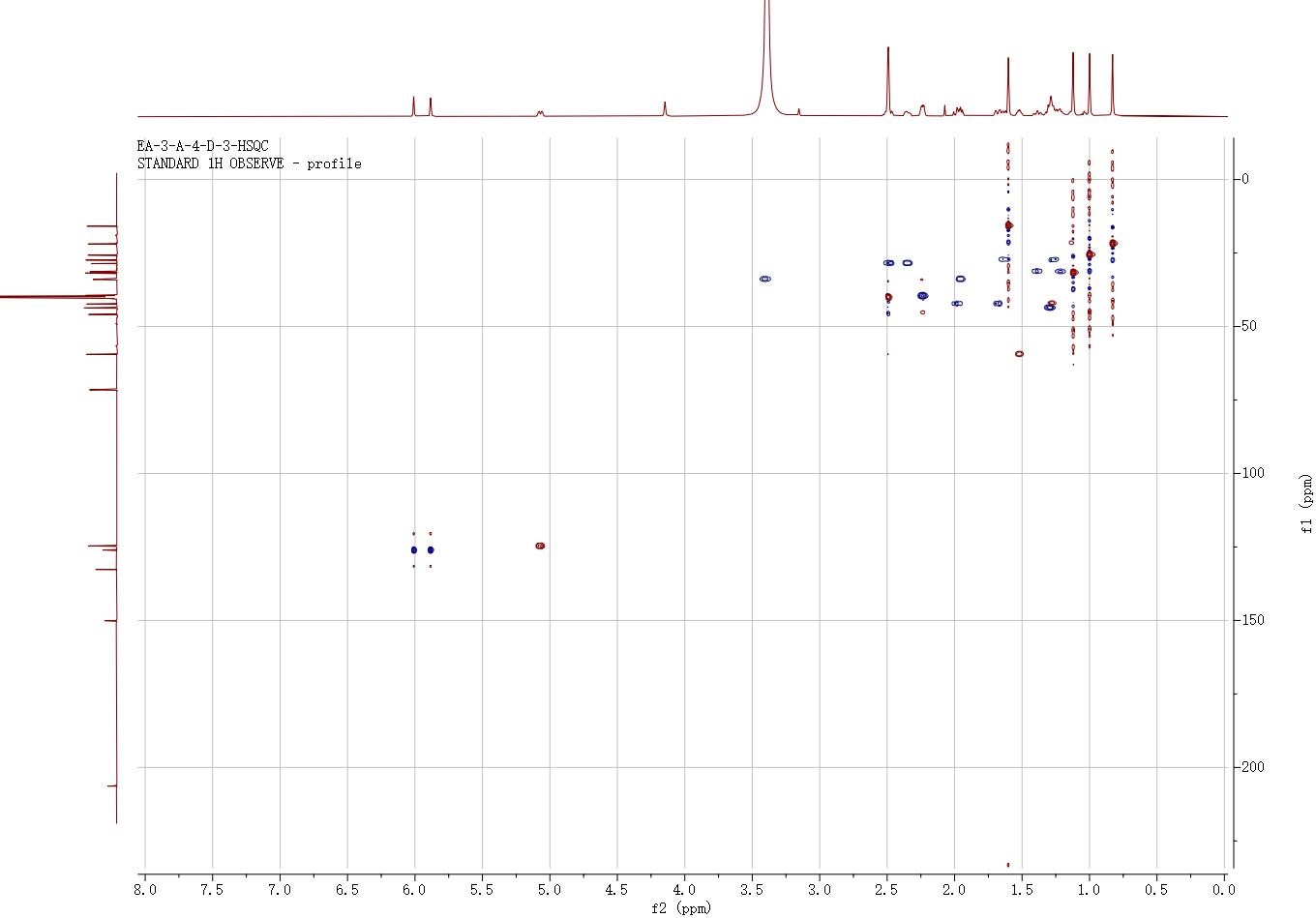


**Figure S85** HSQC spectrum of compound **20**


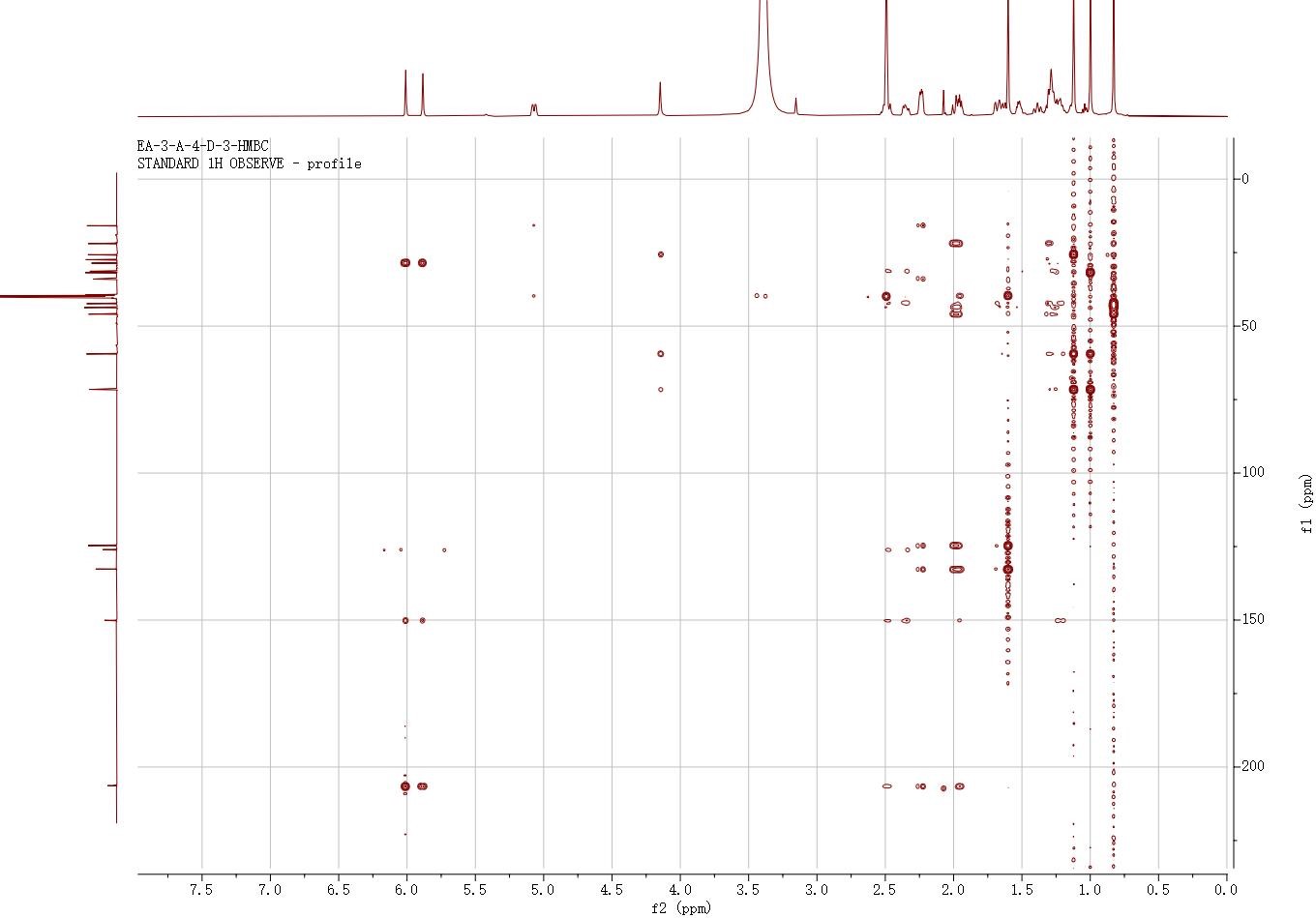


**Figure S86** HMBC spectrum of compound **20**


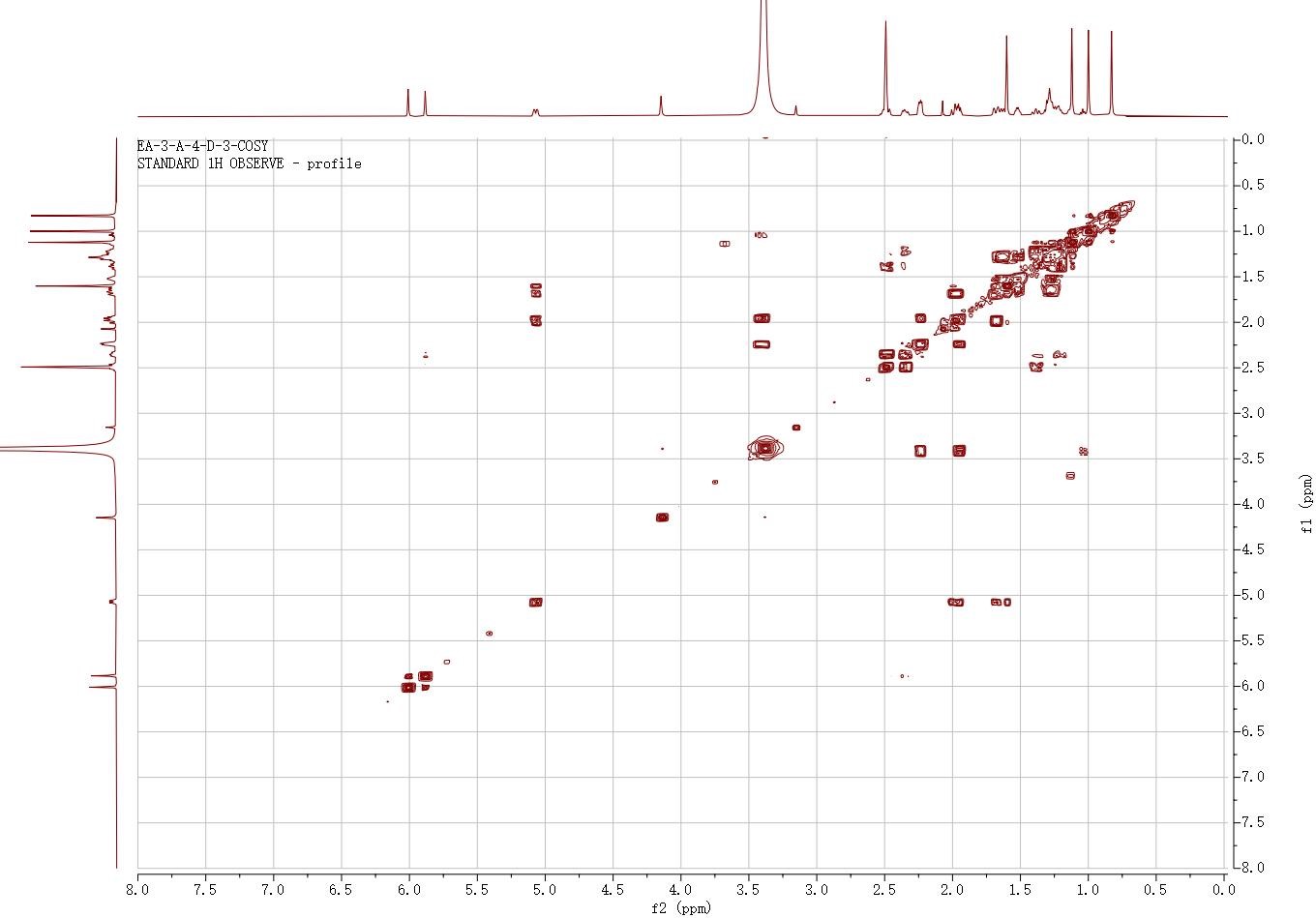


**Figure S87** ^1^H-^1^H COSY spectrum of compound **20**


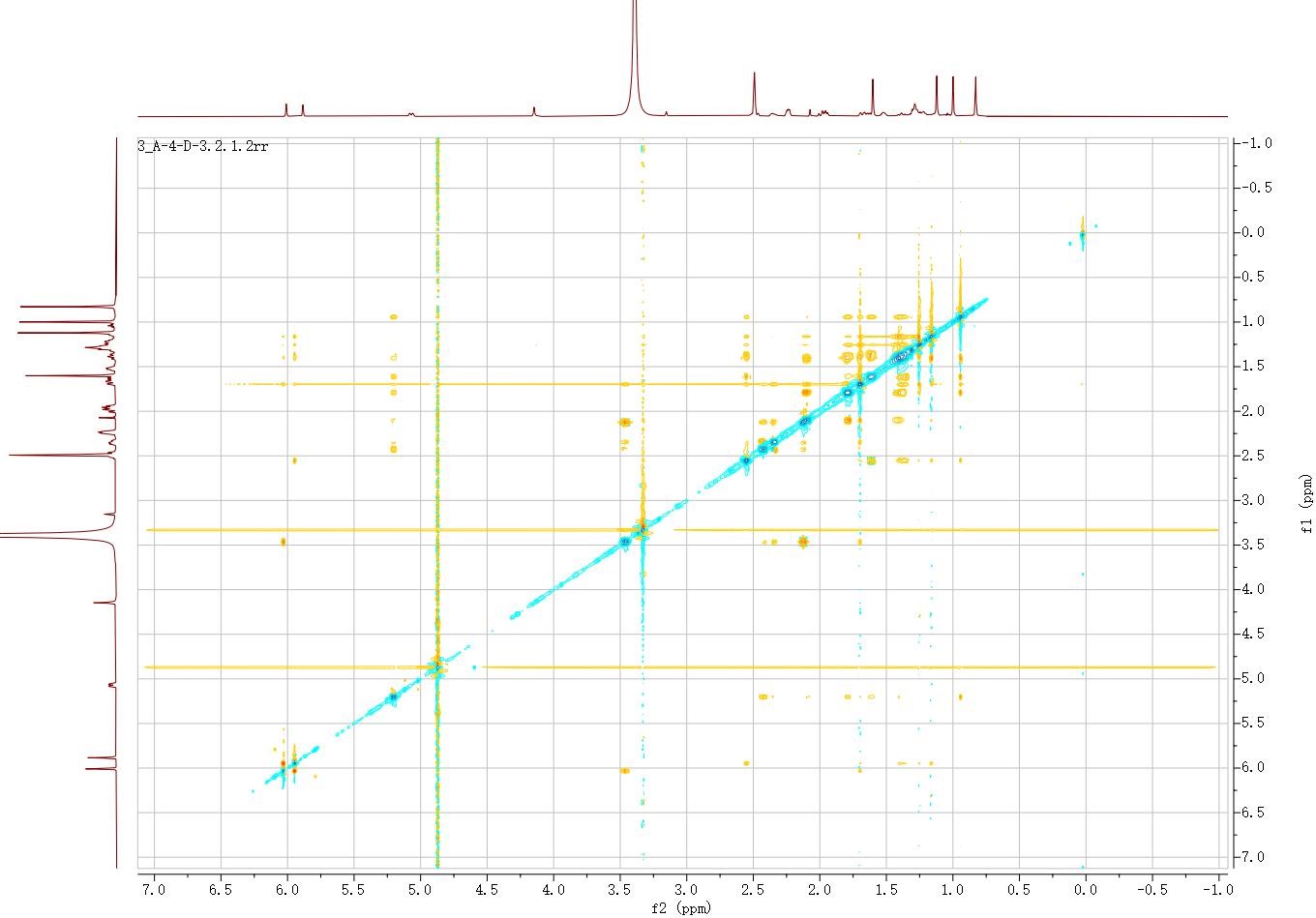


**Figure S88** NOESY spectrum of compound **20**

**Figure S89** HRESIMS of compound **21**


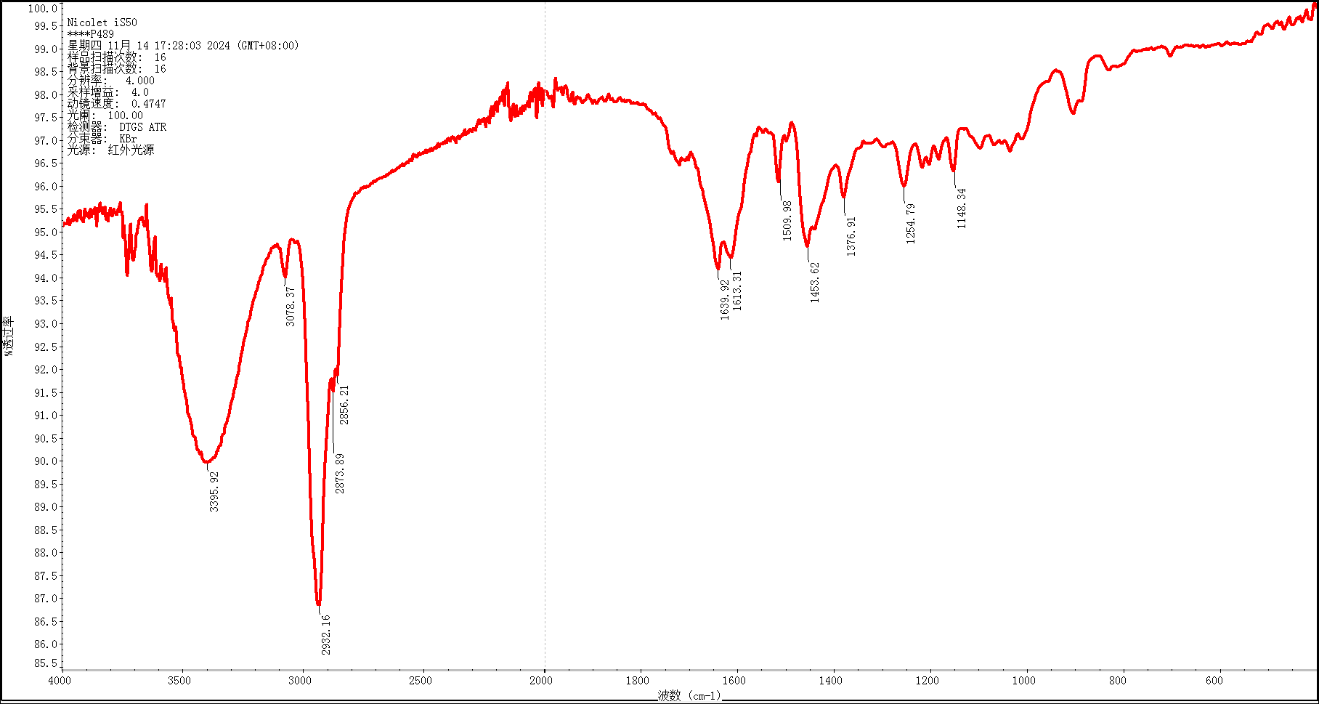


**Figure S90** IR spectrum of compound **21**

**
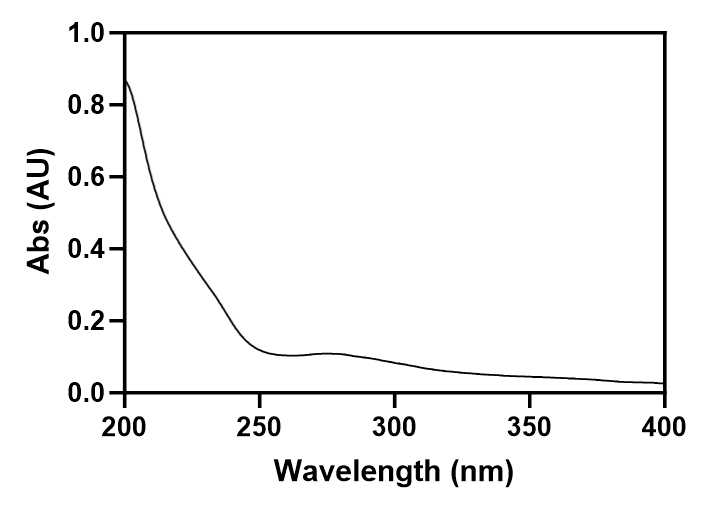
Figure S91** UV spectrum of compound **21**


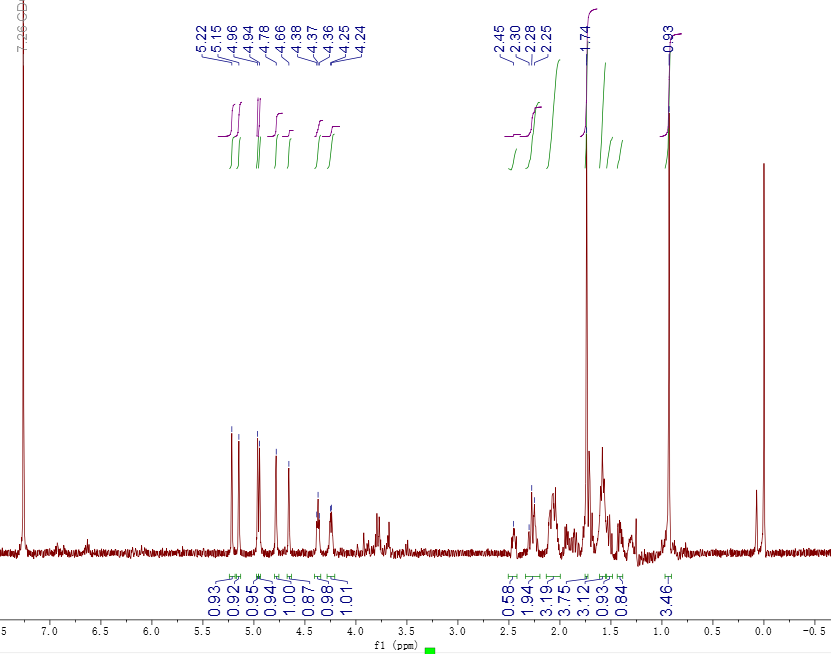


**Figure S92** ^1^H-NMR spectrum of compound **21** (500 MHz, chloroform-*d*_1_)


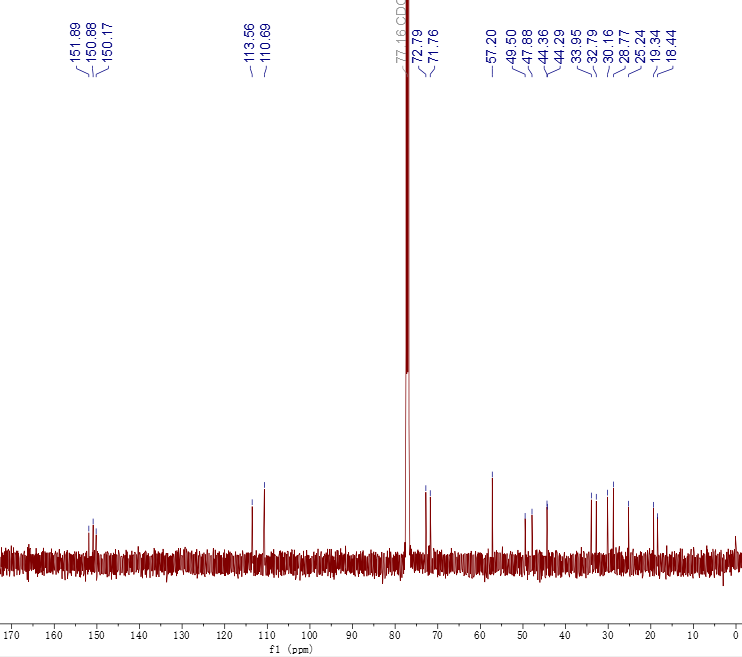


**Figure S93** ^13^C-NMR spectrum of compound **21** (125 MHz, chloroform-*d*_1_)

**
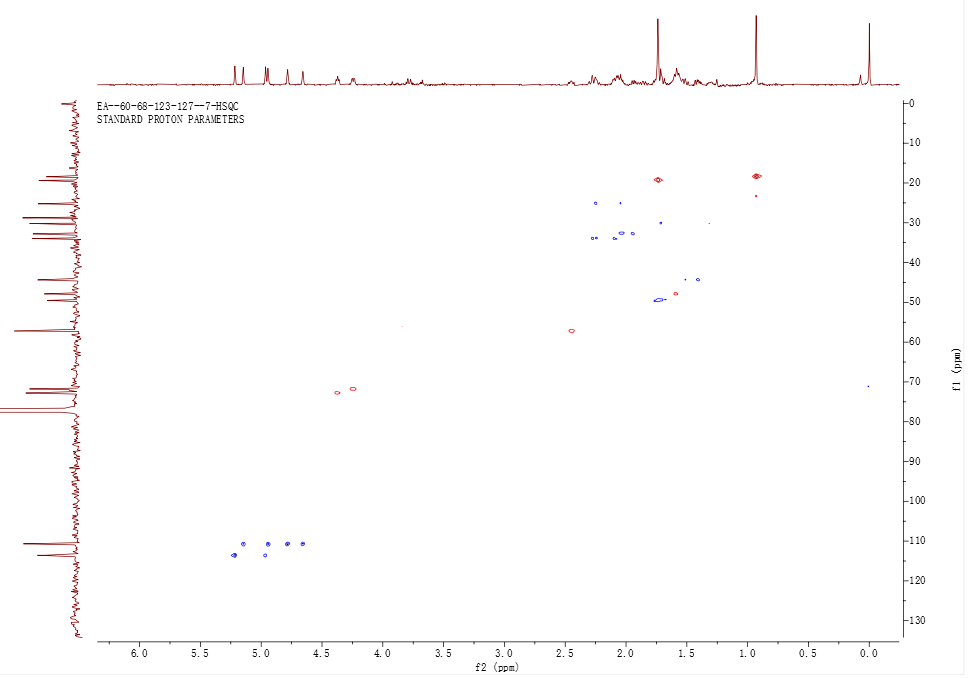
**

**Figure S94** HSQC spectrum of compound **21**


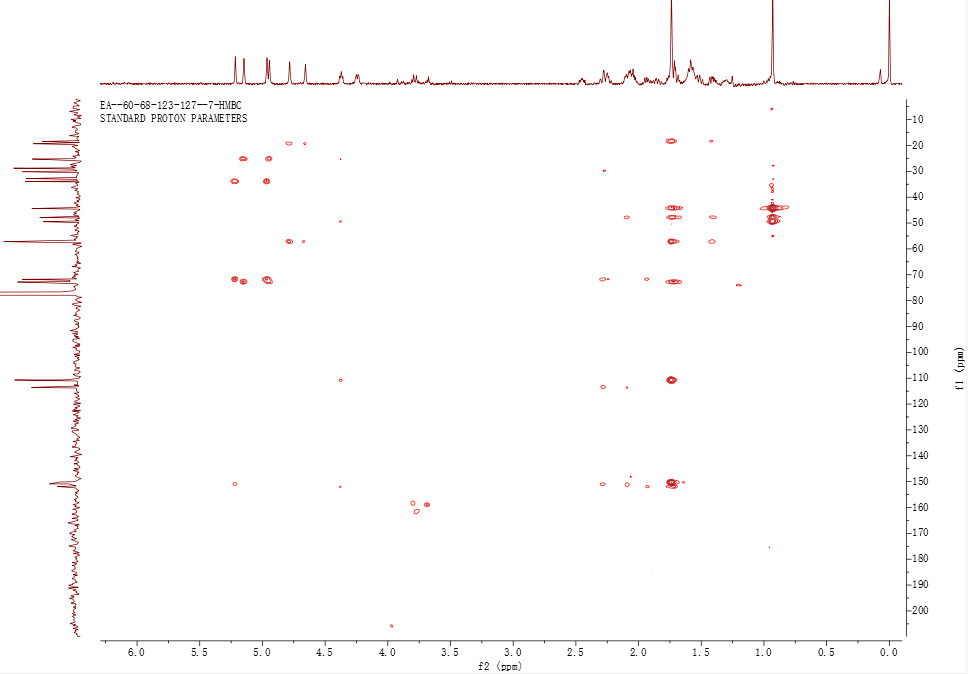


**Figure S95** HMBC spectrum of compound **21**

**
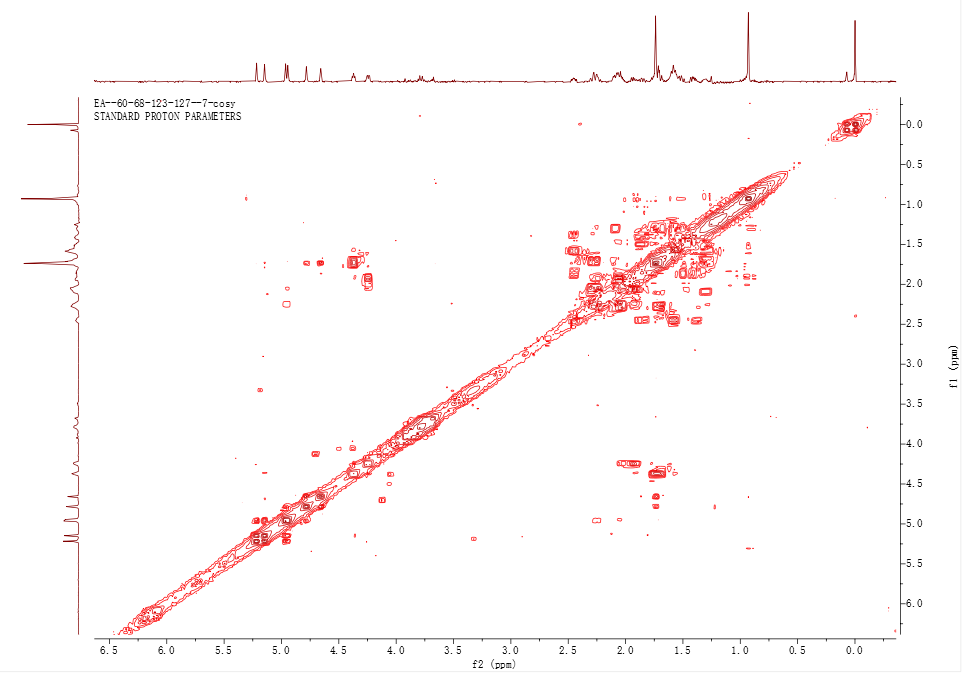
**

**Figure S96** ^1^H-^1^H COSY spectrum of compound **21**


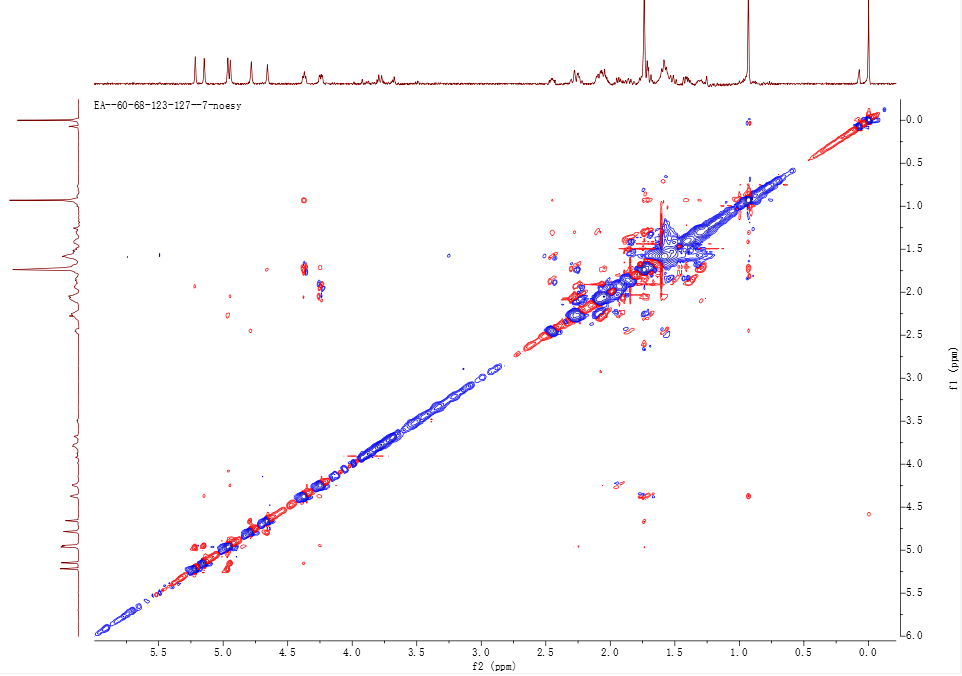


**Figure S97** NOESY spectrum of compound **21**

| **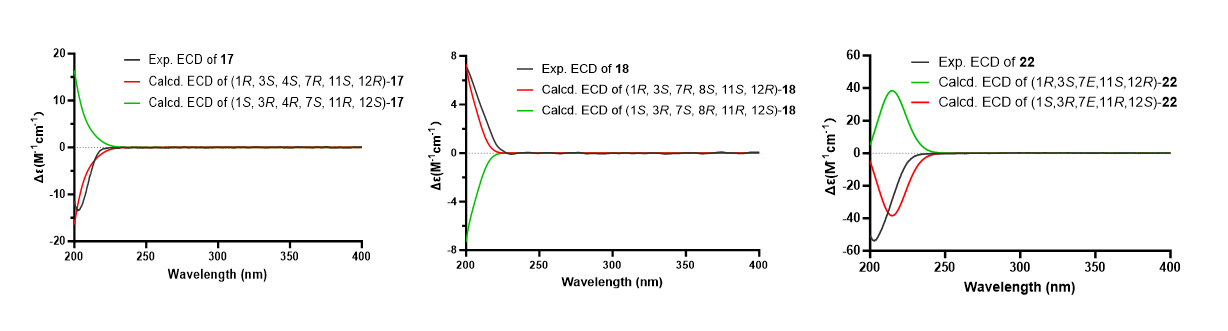** |
| --- |

**Figure S98** The experimental and calculated ECD spectra of compounds **17**–**18**, and **22**.
